# Brigatinib causes tumor shrinkage in both NF2-deficient meningioma and schwannoma through inhibition of multiple tyrosine kinases but not ALK

**DOI:** 10.1371/journal.pone.0252048

**Published:** 2021-07-15

**Authors:** Long-Sheng Chang, Janet L. Oblinger, Abbi E. Smith, Marc Ferrer, Steven P. Angus, Eric Hawley, Alejandra M. Petrilli, Roberta L. Beauchamp, Lars Björn Riecken, Serkan Erdin, Ming Poi, Jie Huang, Waylan K. Bessler, Xiaohu Zhang, Rajarshi Guha, Craig Thomas, Sarah S. Burns, Thomas S. K. Gilbert, Li Jiang, Xiaohong Li, Qingbo Lu, Jin Yuan, Yongzheng He, Shelley A. H. Dixon, Andrea Masters, David R. Jones, Charles W. Yates, Stephen J. Haggarty, Salvatore La Rosa, D. Bradley Welling, Anat O. Stemmer-Rachamimov, Scott R. Plotkin, James F. Gusella, Justin Guinney, Helen Morrison, Vijaya Ramesh, Cristina Fernandez-Valle, Gary L. Johnson, Jaishri O. Blakeley, D. Wade Clapp, on behalf of the Synodos for NF2 Consortium

**Affiliations:** 1 The Research Institute at Nationwide Children’s Hospital and Department of Pediatrics, Center for Childhood Cancer and Blood Diseases, The Ohio State University College of Medicine, Columbus, Ohio, United States of America; 2 Department of Pediatrics, Indiana University, School of Medicine, Indianapolis, Indiana, United States of America; 3 Division of Preclinical Innovation, National Center for Advancing Translational Sciences, National Institutes of Health, Bethesda, Maryland, United States of America; 4 University of North Carolina School of Medicine, Chapel Hill, North Carolina, United States of America; 5 Burnett School of Biomedical Sciences, College of Medicine, University of Central Florida, Lake Nona-Orlando, Florida, United States of America; 6 Massachusetts General Hospital and Department of Neurology, Center for Genomic Medicine, Harvard Medical School, Boston, Massachusetts, United States of America; 7 Leibniz Institute on Aging–Fritz-Lipmann Institute (FLI), Jena, Germany; 8 Division of Pharmacy Practice and Science, The Ohio State University College of Pharmacy, Columbus, Ohio, United States of America; 9 Department of Otolaryngology and Head/Neck Surgery, Indiana University School of Medicine, Indianapolis, Indiana, United States of America; 10 Children’s Tumor Foundation, New York, New York, United States of America; 11 Department of Otolaryngology, Massachusetts Eye and Ear Infirmary, Massachusetts General Hospital and Harvard University, Boston, Massachusetts, United States of America; 12 Department of Pathology, Massachusetts General Hospital and Harvard Medical School, Boston, Massachusetts, United States of America; 13 Center for Genomic Medicine, Massachusetts General Hospital and Department of Genetics, Blavatnik Institute, Harvard Medical School, Boston, Massachusetts, United States of America; 14 Sage Bionetworks, Seattle, Washington, United States of America; 15 Departments of Neurology, Neurosurgery and Oncology, Johns Hopkins University School of Medicine, Baltimore, Maryland, United States of America; Marshall University, Joan C. Edwards School of Medicine, UNITED STATES

## Abstract

Neurofibromatosis Type 2 (NF2) is an autosomal dominant genetic syndrome caused by mutations in the *NF2* tumor suppressor gene resulting in multiple schwannomas and meningiomas. There are no FDA approved therapies for these tumors and their relentless progression results in high rates of morbidity and mortality. Through a combination of high throughput screens, preclinical *in vivo* modeling, and evaluation of the kinome *en masse*, we identified actionable drug targets and efficacious experimental therapeutics for the treatment of NF2 related schwannomas and meningiomas. These efforts identified brigatinib (ALUNBRIG^®^), an FDA-approved inhibitor of multiple tyrosine kinases including ALK, to be a potent inhibitor of tumor growth in established *NF2* deficient xenograft meningiomas and a genetically engineered murine model of spontaneous NF2 schwannomas. Surprisingly, neither meningioma nor schwannoma cells express ALK. Instead, we demonstrate that brigatinib inhibited multiple tyrosine kinases, including EphA2, Fer and focal adhesion kinase 1 (FAK1). These data demonstrate the power of the *de novo* unbiased approach for drug discovery and represents a major step forward in the advancement of therapeutics for the treatment of NF2 related malignancies.

## Introduction

Neurofibromatosis Type 2 (NF2) is a rare disease in which biallelic loss of the *NF2* gene leads to the development of tumors of neural crest derived origin [1–3]. *NF2* encodes the tumor suppressor Merlin, whose biochemical function is incompletely understood [4]. Bilateral vestibular schwannomas are pathognomonic for NF2 and the condition is also commonly associated with the development of multiple meningiomas. Further, approximately 90% of sporadic schwannomas and 60% of sporadic meningiomas, the most common intracranial tumor in humans, have inactivation of *NF2* [5,6]. There are no approved drug therapies for these common and relentlessly progressive tumors.

In this study, we applied a systems biology approach to: (1) utilize high throughput drug screens to identify FDA approved or late stage development compounds active against Merlin deficient schwannoma and meningioma cells, (2) integrate data from kinome profiling and transcriptome analysis to prioritize targets for future development and (3) utilize a genetically engineered mouse model and tumor xenograft model to establish preliminary preclinical efficacy data for a subset of compounds identified via the *in vitro* drug screening approaches with the goal of identifying at least one drug to move forward to a clinical trial. Using this approach, we have identified for the first time, single and combinatorial agents that positively impact the treatment of both schwannomas and meningiomas associated with *NF2*. Importantly, we established the FDA approved, multi-tyrosine kinase inhibitor (TKI), brigatinib, as a potent inhibitor of Merlin-deficient schwannoma and meningioma growth, both *in vitro* and in preclinical animal models. Interestingly, neither Merlin-deficient schwannoma nor meningioma cells express the primary published target of brigatinib, anaplastic lymphoma kinase (ALK). Rather, we demonstrate that mechanistically, this compound inhibits multiple kinases, including focal adhesion kinase (FAK) previously recognized as an oncoprotein in *NF2* related tumors [7–9]. These data demonstrate an effective systems biology approach for preclinical therapeutic discovery applicable to rare tumors and support the advancement of brigatinib, as a multi-TKI into clinical trials for the treatment of *NF2* associated meningiomas and schwannomas.

## Results

### Discovery of novel drug combinations for NF2-associated meningiomas and schwannomas

**Fig 1:**
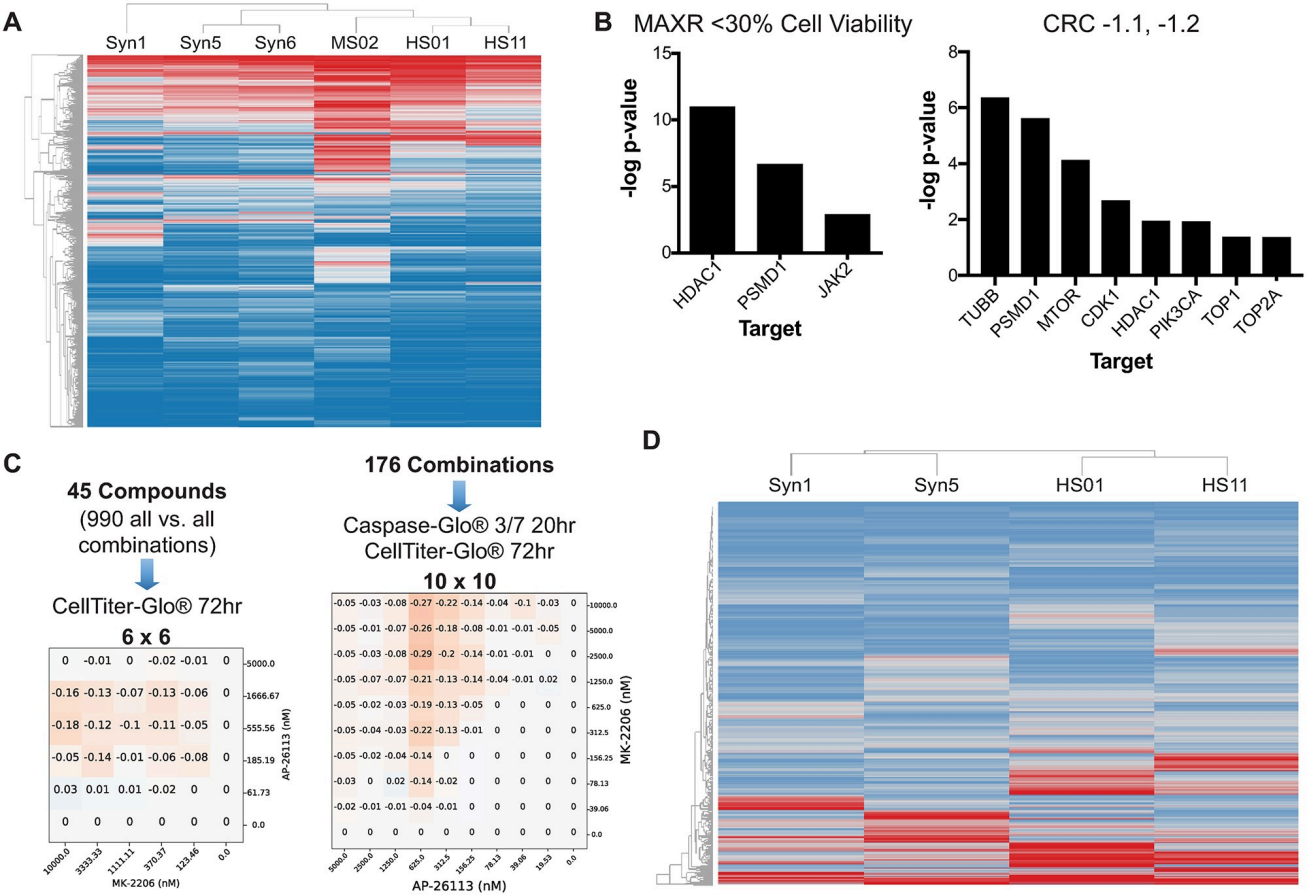
Single-agent and matrix HTS of the oncology MIPE 4.0 collection in meningioma and schwannoma cells. (A) Heat map of pharmacological responses (as % cell viability at the maximum dose tested, 46 μM, also called maximum response) of the MIPE 4.0 library in *NF2*-expressing Syn1 and *NF2*-null Syn5 human arachnoidal cells, *NF2*-deficient Ben-Men-1 (Syn6) meningioma cells, *NF2*-expressing HS11 and *NF2*-knockdown HS01 human Schwann cells, and *Nf2*^-/-^ MS02 mouse schwannoma cells. Responses were clustered using a Hierarchical Clustering in Spotfire TIBCO. Red indicates low % cell viability and blue is high viability. (B) Target enrichment plots for pan active hits selected by % viability at the maximum concentration of compound tested (MAXR) <30% for both cell lines and all assay modes (top panel) and for pan active hits selected by CRCs -1.1 or -1.2 for both cell lines and all assay modes (bottom). The -log p-values were calculated as follows: Given a selection of compounds, the annotated targets for these compounds were identified and the enrichment for each target computed, compared to background, using the Fishers exact test. For this test, the background was defined as all the targets annotated in the MIPE collection. The p-value from the test was adjusted for multiple hypothesis testing using the Benjamini-Hochberg method. A -log p-value >1 was used as a cut-off to consider a target or processed being overrepresented. (C) Number of combinations tested and assays used for the 6x6 and 10x10 matrix combination HTS. Delta bliss 6x6 and 10x10 matrix plots for the combination of MK-2206 and ALK-IN-1 in Syn5 cells, using the CellTiterGlo viability assay. Darker color indicates higher DeltaBliss scores for synergy. (D) Heat map of DeltaBliss Sum Negative for the 990 6x6 all vs. all pairwise combinations of the 45 drugs selected from the single-agent testing. The IC_50_ and maximum % inhibition of each compound in each indicated cell line were calculated. Responses were clustered using a Hierarchical Clustering in Spotfire TIBCO. Red indicates low % cell viability and blue is high viability.

To identify novel chemotherapeutic agents for *NF2*-associated tumors, we screened the NCATS MIPE library 4.0 of 1,932 compounds in a high-throughput dose-response format [9] using the isogenic pairs of *NF2*-expressing HS11 and *NF2*-deficient HS01 Schwann cells and *NF2*-expressing Syn1 and *NF2*-null Syn5 arachnoidal cells, as well as *Nf2*^-/-^ mouse MS02 schwannoma cells and human Ben-Men-1 meningioma cells (Syn6). This panel of cell lines was used to assess selectivity of pharmacological responses based on *NF2* status and to distinguish the differences in compound responses between human and mouse *NF2*-related tumor cells to better predict the drug activity in animal models. The MIPE library was initially screened as single agents in dose-response mode, to determine the potency (IC_50_), efficacy (maximum % inhibition), and area under the curve (AUC) for each compound in each cell line. Active compounds were selected using dose-response curve analysis algorithms, which assigns each tested compound a Curve Response Class (CRC) number [10]. This method classified primary hits into different categories according to their IC_50_, magnitude of response, quality of curve fitting (r^2^), and number of asymptotes. A CRC of -1.1 represents a compound with a complete curve and with high efficacy; a CRC of -1.2 represents a compound with a complete curve but with partial efficacy. Compounds with CRC 4 are inactive. Compounds with CRCs of -1.1, -1.2, and a maximum response of <30% cell viability were considered for further testing in a combination screen. The heat map of the maximal % activity using the hierarchical clustering function in Spotfire Tibco is shown in Fig 1A. The results of this single-agent screen demonstrated that the pharmacological responses clustered cells by tumor type (meningioma versus schwannoma) rather than *NF2*-expressing versus *NF2*-deficient cells. S1 Fig provides a view of the pharmacological responses according to different relevant targets, cellular pathways and mechanisms, based on the AUC parameters which include both potency and efficacy of the compounds. This high-level biological analysis of pharmacological responses confirms that all cell lines tested, independent of Merlin status or tumor type, behaved very similarly and that compounds targeting proteasome, chaperone, topoisomerase, RTK, serine-threonine kinase (STK), phosphatidylinositol 3-kinase (PI3K), and tubulin are the most active (S1 Fig). 62 and 82 compounds were active in all cells by a maximum response criteria of <30% cell viability and CRC of -1.1 and -1.2, respectively. Compounds with CRC -1.1 were hypothesized to be cytotoxic, while compounds that were -1.2 could either be cytostatic or induce senescence and slow cell growth. Targets that were enriched for these active compounds included inhibitors of histone deacetylase (HDAC), proteasome, Janus kinase 2 (JAK2), tubulin, mammalian target of rapamycin (mTOR), and cyclin-dependent kinase 1 (CDK1), PI3K, and topoisomerase (Figs 1B and S1).

We then selected 45 compounds to be tested in combination based on: (1) the potency and efficacy in each cell line, (2) the targets in key pathways regulated by Merlin, and (3) mechanisms of interest identified by genome and kinome analyses. These compounds were first tested using the CellTiterGlo assay at 72-h, in 6x6, all versus all dose-response matrices, for a total of 990-pairwise combinations (Fig 1C), with concentrations customized to include a range > 4-fold and < 4-fold the IC_50_, to better determine possible synergies in both the meningioma (Syn1 vs Syn5) and the schwannoma cell (HSO1 vs HS11) models. Synergistic combinations were ranked based on the Delta Bliss Sum Negative (DBSumNeg) values [11] for each pairwise combination matrix. We detected 33 combinations that had a DBSumNeg value of <-2 in *Nf2-*deficient Syn5 cells, and 69 combinations, which had a DBSumNeg of <-2 in HS01 cells. A heat map of hierarchical clustering of DBSumNeg for each pairwise compound combination in each cell line showed that meningioma and schwannoma cells each clustered separately by DBSumNeg synergy scores, regardless of *NF2* status as seen in the single agent screen (Fig 1D).

**Fig 2:**
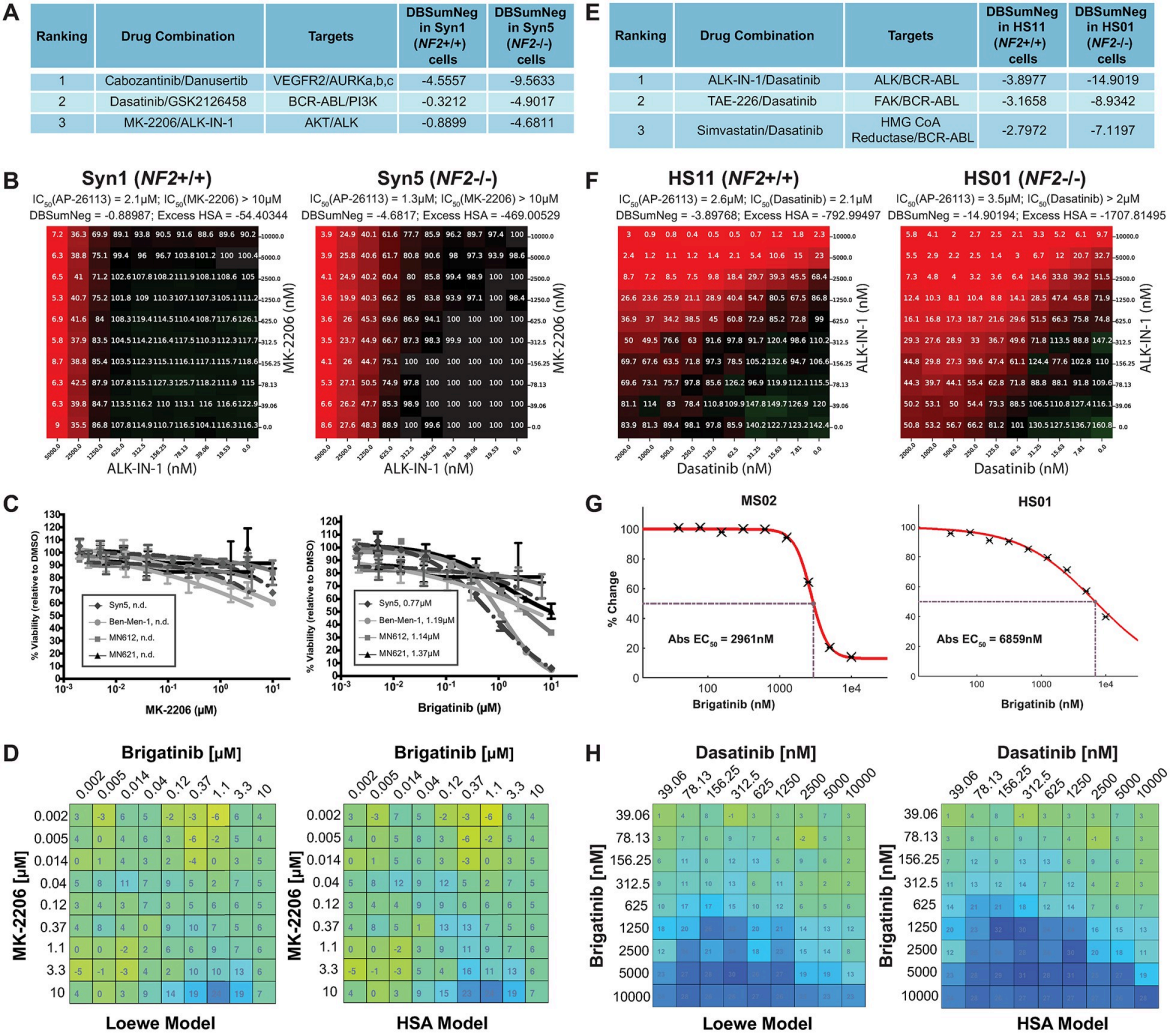
Like ALK-IN-1, brigatinib exhibited growth-inhibitory synergy with MK-2206 or dasatinib in meningioma and schwannoma cells. (A) The top synergistic drug combinations identified for *NF2*-deficient meningioma cells. (B) 10x10 dose-viability response matrix of ALK-IN-1 vs MK-2206 in Syn1 and Syn5 cells, assessed at 72h. (C) Single-drug dose-response curves (DRCs) for MK-2206 (left) or brigatinib (27) were generated for human *NF2*-null cell lines, including Syn5, Ben-Men-1, and two primary meningiomas (MN612, MN621). Drug concentrations are outlined under Supplementary Methods in S1 File. n.d., not determined. Data is expressed as +/- SEM. (D) Combination treatment of Ben-Men-1 cells was carried out in a 10x10 dose-matrix format using the same increasing concentration range of MK-2206 and brigatinib as with single DRCs. Heatmaps were generated on a colorimetric scale using Combenefit software, which calculates the drug interaction effects (relative to vehicle/DMSO control). (E) The top synergistic drug combinations identified for NF2-related schwannoma cells. (F) 10x10 dose-viability response matrix of ALK-IN-1 vs dasatinib in HS11 and HS01 cells, assessed at 72h. (G) Brigatinib dose-response in mouse MS02 and human HS01 cells at 60 and 72h, respectively. (H) Loewe and highest single agent (HSA) synergy matrix analysis of brigatinib vs. dasatinib in HS01 cells treated for 72h.

Next we selected 176 pairwise compound combinations based on DBSumNeg values, synergy selectivity for *NF2*-deficient cells, mechanisms of interest, and clinical interest and tested them in 10x10 dose-response matrix format with the same set of meningioma and schwannoma cell models using both a cell viability readout (CellTiterGlo at 72h) and an apoptosis readout (CaspaseGlo, 20h). Based on differential drug synergy in *NF2*-deficient versus *NF2*-expressing cells, the stage of drug development, and compound performance in prior preclinical and clinical evaluations including pharmacology, response rate, toxicity, and availability, we selected and ranked the top three drug combinations for each *NF2* tumor type. These combinations in meningioma cells included: cabozantinib (an inhibitor of RTKs, including vascular endothelial growth factor receptor 2 [VEGFR2] and hepatocyte growth factor receptor MET)/danusertib (an Aurora kinase inhibitor), dasatinib (an inhibitor of BCR-ABL and SRC-family TKs)/GSK2126458 (a dual PI3K/mTOR inhibitor), and MK-2206 (an AKT inhibitor)/ALK-IN-1 (an inhibitor of ALK and multiple other TKs) (Fig 2A). The most effective combinations in Schwann cells were slightly different and included: ALK-IN-1/dasatinib, TAE226 (a dual FAK/IGF-1R inhibitor)/dasatinib, and dasatinib/simvastatin (an HMG-CoA reductase inhibitor) for schwannoma (Figs 2E, S3 and S4).

### ALK-IN-1 and its derivative brigatinib synergized with MK-2206 or dasatinib to inhibit *NF2*-deficient meningioma and schwannoma cell viability, respectively

ALK-IN-1 is a chemical analogue of the FDA-approved ALK inhibitor brigatinib (AP26113. ALUNBRIG^®^) [12]. As ALK-IN-1 is no longer in clinical development, we compared brigatinib with ALK-IN-1 for their ability to synergize with MK-2206 or dasatinib to inhibit viability of *NF2*-deficient cells. Similar to ALK-IN-1, brigatinib inhibited viability of *NF2*-deficient Syn5 arachnoidal and Ben-Men-1 meningioma cells and two independent primary meningioma cell lines with *NF2* loss (MN612 and MN621). MK-2206 alone showed little growth-inhibitory activity (Fig 2B and 2C), however, when combined with brigatinib, MK-2206 exhibited mild synergy in growth inhibition of Ben-Men-1 (Fig 2D) and Syn5, MN612, and MN621 cells (S2 Fig).

ALK-IN-1 and dasatinib combination treatment reduced cell viability in the human Schwann cell lines, and the *NF2*-deficient (HS01) cells were more sensitive to treatment (Fig 2F). As a single agent, brigatinib inhibited viability of mouse *Nf2*^*-/-*^ schwannoma MS02 and human *NF2*-deficient HS01 Schwann cells (Fig 2G). Mouse MS02 cells appeared more sensitive to brigatinib inhibition than human HS01 cells. As with ALK-IN-1 in the broad screen (Fig 2F), brigatinib demonstrated significant synergy with dasatinib in reducing viability of both HS01 and MS02 cells (Figs 2H and S3). Based on these results, we further evaluated brigatinib monotherapy and in combination with either MK-2206 or dasatinib by transcriptome analysis of treated cells and for effects on tumor growth *in vivo*.

### Transcriptome analysis of drug-treated *NF2*-expressing and *NF2*-deficient cells

Baseline transcriptome analysis revealed that ALK is not expressed in Syn1/Syn5 arachnoidal and HS01/HS11 Schwann cells, suggesting that brigatinib acts through another target. Consistent with its differential effect on their growth, brigatinib treatment, with (BTM) or without MK-2206 (BT), elicited an exaggerated transcriptomic response from Syn5 compared to Syn1 cells. MK-2206 treatment alone did not result in any DEGs. Most differentially expressed genes (DEGs) from treatment of Syn1 (560 BT and 830 BTM) were also DEGs in treated Syn5 (456 BT and 625 BTM), but the latter expressed far more DEGs overall (2605 BT and 2171 BTM of which 1658 DEGs were present and altered in the same direction by both treatments) (S5A Fig and S1 Table). DEGs shared by treated Syn5 and Syn1 were enriched in DNA replication and cell cycle pathways, but the more numerous Syn5-specific DEGs were enriched in pathways related to RNA (S2A Table). Interestingly, in untreated cells, *NF2* inactivation is associated with a similar level of dysregulation to that caused by drug treatment of Syn5 (2458 DEGs from untreated Syn5 vs untreated Syn1). A comparison of these 2458 DEGs caused by *NF2* inactivation with the DEGs elicited in Syn 5 by either brigatinib or brigatinib + MK-2206 revealed substantial overlap (988 and 832, respectively), with more than 99% of these shared DEGs being dysregulated in opposite directions by *NF2* inactivation and by drug treatment of the *NF2* deficient cells (S5B Fig and S1 Table).

Treatment of HS01 and HS11 cells with brigatinib did not significantly disrupt the transcriptome, while treatment with either dasatinib or simvastatin had modest effects (S1 Table, S5C Fig). The combination of brigatinib + dasatinib showed more robust effects, with DEGs in HS01 Schwann cells being enriched most significantly in cytoplasmic pathways, in contrast to the arachnoidal cells (S2B Table).

### Kinome profiling in brigatinib- treated cells

**Fig 3:**
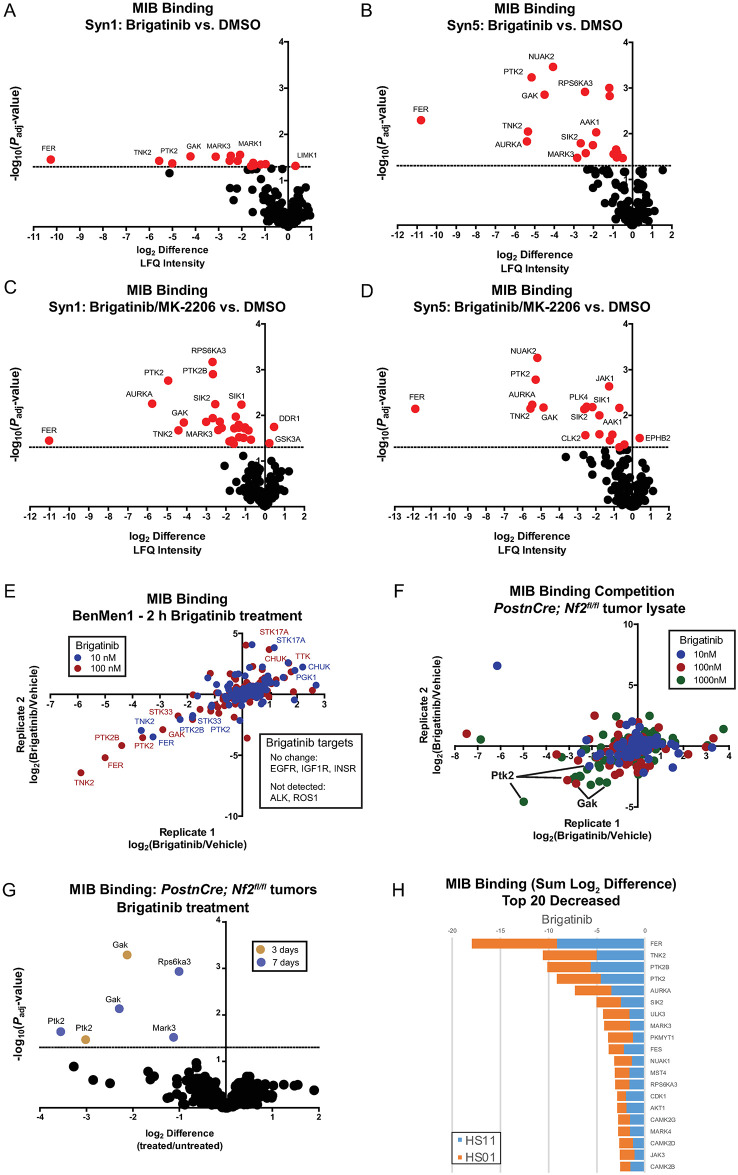
MIB/MS kinome profiling reveals brigatinib target specificity in multiple *NF2*-deficient cell models. (A-D) MIB/MS kinome profiling was performed following treatment of Syn1 and Syn5 cells with vehicle (DMSO), brigatinib (1μM) or the combination of MK-2206 (0.5μM) and brigatinib (1μM) for 24h in biological triplicate. MIB binding (log2LFQ intensities) were used for comparisons and the volcano plots indicate log2 difference and significance (5% FDR, indicated by dashed line). (E) MIB/MS kinome profiling was performed following treatment of Ben-Men-1 cells with vehicle (DMSO) or brigatinib (10nM, blue or 100nM, red) for 2h in biological duplicate. The log2 difference in MIB binding (LFQ intensity) was calculated and plotted relative to vehicle for each replicate. (F) Tumor lysates from the *Postn-Cre*;*Nf2*^*flox/flox*^ mouse model were prepared and equal amounts of total protein were incubated for 2h with vehicle (0.001% ethanol) or brigatinib (10nM, blue; 100nM, red; 1000nM, green) in biological duplicate prior to MIB/MS kinome profiling. The log2 difference in MIB binding (LFQ intensity) was calculated and plotted relative to vehicle for each replicate. (G) *Postn-Cre*;*Nf2*^*flox/flox*^ mice were treated with vehicle or brigatinib for 3 or 7 days (gold and blue, respectively, for indicated kinases). Tumors were harvested and subjected to MIB/MS kinome profiling. MIB binding (log2LFQ intensities) was determined and used for comparisons of brigatinib to vehicle treatment. The volcano plot indicates log2 difference and significance (5% FDR, indicated by dashed line). (H) HS11 and HS01 cells were treated with vehicle (DMSO) or brigatinib (1μM) for 24h prior to MIB/MS kinome profiling. MIB binding (log2LFQ intensities) was determined and the top 20 kinases with decreased MIB binding compared to vehicle are shown as a stacked bar plot for the two cell lines.

Brigatinib is used clinically for the treatment of ALK-positive non small cell lung carcinoma, with reported IC50 of 0.6nM determined by in vitro kinase assay [13]. However, it has numerous kinase targets including FER, ROS1, FLT3, EGFR, FAK1 (PTK2), and KIT with IC50 values <250nM and additional kinases with IC50 ~500nM-1uM such as ABL, EPHA1, EPHA7, and EPHB1 [13,14]. As brigatinib administration at 90 mg or 180mg dosage has been shown to achieve Cmax of 552 (0.94 uM) or 1452 ng/ml (2.482 uM), respectively [15], we sought to determine the kinases affected using comparable or lower concentrations of brigatinib effective in schwannoma and meningioma cells. To address this question, we conducted multiplexed kinase inhibitor bead (MIB) kinome profiling experiments. MIBs are a mixture of Type I kinase inhibitors designed to bind kinases when they are in the “Asp-Phe-Gly (DFG)-in” (active) conformation [16,17]. A spectrum of MIBs capable of binding almost all expressed kinases is employed, and binding is influenced not only by kinase expression but kinase activity as well. Kinases bound to the MIBs are subsequently identified and quantified via mass spectrometry [17,18]. In *NF2*-expressing (Syn1) or *NF2*-deficient (Syn5) arachnoidal cells treated for 24 hours, brigatinib significantly reduced MIB binding of multiple tyrosine kinases including Aurora kinase A (AURKA), Cyclin G-associated kinase (GAK), Fps/Fes-related kinase (FER), Activated Cdc42-associated kinase (ACK1 or TNK2), and FAK1 (PTK2) (Fig 3A and 3B). Similar target effects were observed with the addition of MK-2206, an allosteric AKT inhibitor, to brigatinib though unique peptides were only detected for AKT3 and in insufficient samples to quantitate (Fig 3C and 3D).

By adding kinase inhibitors to lysate or briefly treating cells, the direct target spectrum can be more reliably assessed by kinome profiling. This overall approach has substantially informed our knowledge of polypharmacology for numerous inhibitors in clinical use [19]. As validation of the observed kinase targets in Syn1 and Syn5 cells, 2-hour incubation of 10 and 100nM brigatinib was performed prior to kinome profiling in an *NF2*-deficient meningioma cell line Ben-Men-1 (Fig 3E). Brigatinib reproducibly reduced MIB binding of FER, TNK2, and FAK1 (PTK2) at both 100 and 1000nM. In a compatible experiment using lysate prepared from schwannomas from a genetically engineered murine model (GEMM), *Nf2*^flox/flox^;*Postn-Cre* mice, a MIB competition assay identified FAK1 (PTK2) and GAK as the primary targets of brigatinib (Fig 3F). Further, kinome profiling from dorsal root ganglia in *Nf2*^flox/flox^;*Postn-Cre* mice treated for either 3 or 7 days with 50 mg/kg/qd brigatinib monotherapy demonstrated a significant reduction in activated FAK1 (PTK2) and GAK (Fig 3G). The target specificity of brigatinib in HS01 and HS11 cells was also analyzed by lysate treatment followed by kinome profiling and the kinases with the largest reduction in binding were FER, TNK2, FAK1 (PTK2) and FAK2 (PTK2B) (Fig 3H). In comparison, the kinase inhibition profile was distinct for dasatinib, which primarily inhibited Src family kinase and Ephrin receptors (S6B and S6C Fig). Consistent with the transcriptome analysis, ALK was not detected using MIB kinome profiling methodology in either schwannomas or meningiomas, suggesting that non-ALK targets of brigatinib were driving the phenotypic effects.

Many of the kinase targets identified by kinome profiling were published targets of brigatinib, including FAK1 (PTK2), FAK2 (PTK2B), FER, MARK3, MARK1, RPS6KAS3, AURKA, SIK2, JAK1, and STK22 [13]. However, several kinases were not previously published brigatinib targets, such as ACK1 (TNK2), GAK, NUAK2, AAK1, PLK4 and SIK1. Since GAK and ACK1 (TNK2) were strongly inhibited in our models, we performed NanoBRET^™^ TE (target engagement) intracellular kinase assays with brigatinib to determine the IC50 for (ACK1) TNK2 and GAK using FAK1 (PTK2) and FER as positive controls (NanoLuc fusions with ALK are not commercially available). As shown in S6D–S6G Fig, brigatinib potently impacted the BRET ratio for each of these kinases in HEK293T cells: FAK1 (PTK2), IC50 = 573.3nM; FER, IC50 = 303.6nM; GAK, IC50 = 154.6 nM; and TNK2, IC50 = 350.3 nM). Collectively, these data identify brigatinib as a direct and potent inhibitor of multiple kinases, including FAK1 (PTK2) and FAK2 (PTK2B), and novel targets, GAK and TNK2, in both arachnoid and schwannoma cells.

**Fig 4:**
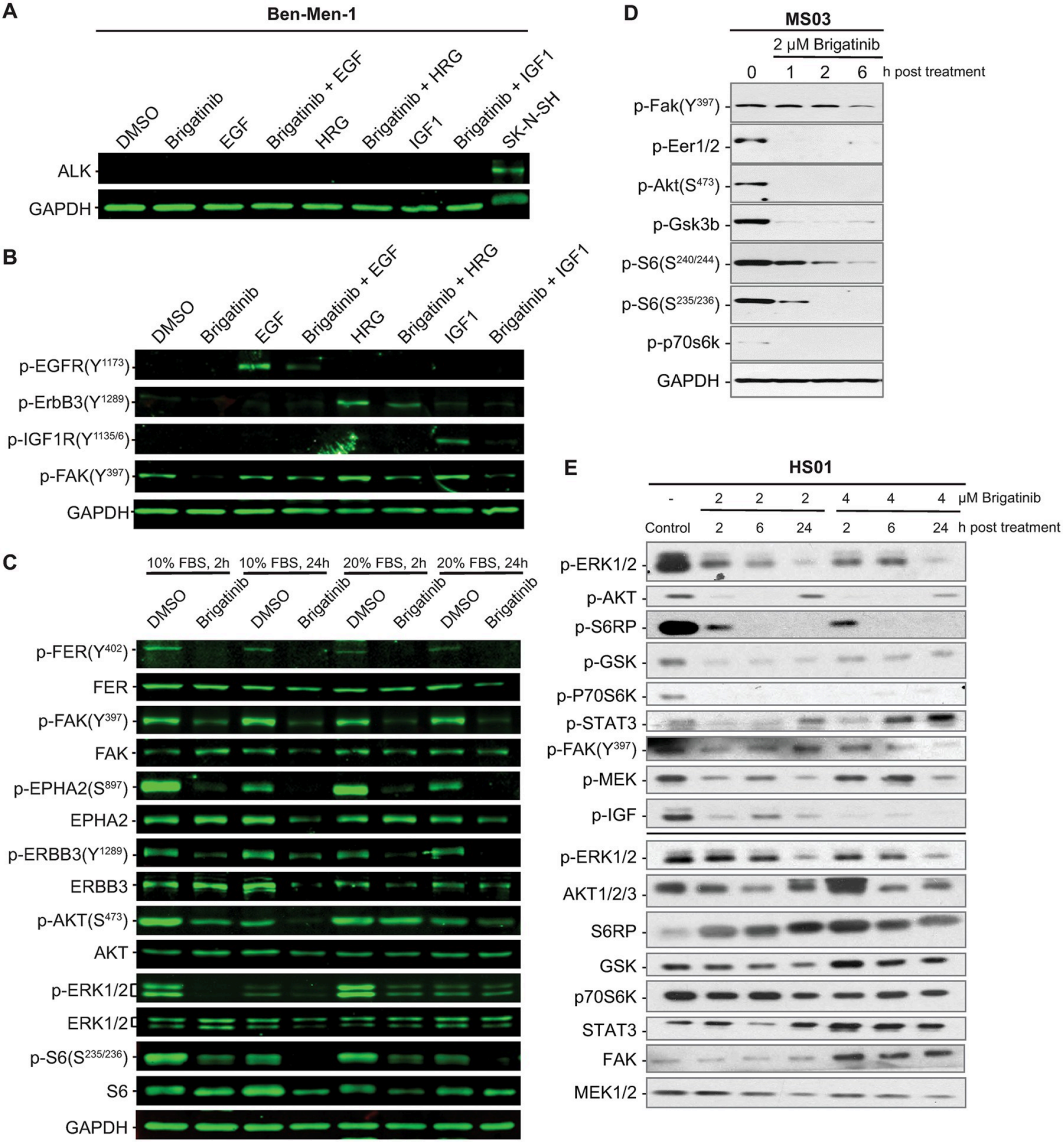
Brigatinib inhibits multiple RTKs, non-RTKs, and their downstream signals. (A) Western blot analysis was conducted to detect ALK expression in Ben-Men-1 cells grown under various growth conditions and with or without brigatinib treatment. (B) Brigatinib treatment greatly reduced phosphorylation of non-RTKs FER and FAK, RTKs EPHA2 and ErbB3, and their downstream signaling molecules AKT, ERK1/2, and S6 in active growing Ben-Men-1 cells or growth-arrested cells stimulated with 20% serum. (C) Brigatinib blocked phosphorylation of EGFR, ErbB3, and IGF1R in growth-arrested Ben-Men-1 cells stimulated with each cognate ligand. (D) Brigatinib reduced the levels of p-FAK, p-ERK1/2, p-AKT, p-S6, and p-Gsk in *Nf2* deficient murine Schwann Cells. (E) Brigatinib reduced phosphorylation of FAK, ERK1/2, MEK, AKT, S6RP, p70S6K, STAT3, and IGR in *NF2*-deficient HS01 human Schwann cells.

For validation of the targets identified in the kinome assay and to further probe known targets of brigatinib which were not altered in the kinome data set, a series of immunoblots were conducted on the Ben-Men-1 cell line. ALK expression was not observed in the Ben-Men-1 cells under any growth conditions (Figs 4A, S7 and S8). When EGF, HRG, or IGF-1 were added to the culture media to stimulate EGFR, ErbB3, and IGF-1R respectively, brigatinib potently inhibited the activation of these receptors (Fig 4B). Low levels of p-EGFR were detected in Ben-Men-1 cells whether actively growing, or growth-arrested and then stimulated with 20% serum. However, p-EGFR could be robustly stimulated with the addition of EGF to the media (S9 Fig). Therefore, it is possible that modulation of these known targets of brigatinib was not observed in the MIB experiments because appropriate stimulatory ligands were not present in those cell culture conditions. At 2 hours and 24 hours post stimulation with 10% or 20% FBS, treatment with brigatinib resulted in a significant reduction in FAK activation (Fig 4C). Treatment with brigatinib also reduced proliferative signaling as measured by p-ERK, p-AKT, p-S6 (Figs 4C and S8). Similarly, in Merlin-deficient (*NF2*-deficient) Schwann cells, ALK expression was not detected at baseline and treatment with brigatinib resulted in a reduction in FAK phosphorylation as well as reductions in p-ERK, p-AKT, and p-S6 (Figs 4D and S10). In human *NF2*-deficient Schwann cells, brigatinib treatment reduced phosphorylation of FAK, ERK, AKT, S6RP, GSK, p70S6K, and IGR at 2, 6 and 24 hours post-treatment with 2μM and 4μM brigatinib, while p-STAT3 and p-MEK were both reduced with 2μM but not 4μM brigatinib (Fig 4E). These data validate and recapitulate the mass spectrometry based findings that brigatinib is a potent inhibitor of FAK in both Merlin-deficient schwannoma and meningioma cell lines *in vitro*.

### Brigatinib treatment of meningiomas and schwannomas in murine models of NF2

**Fig 5:**
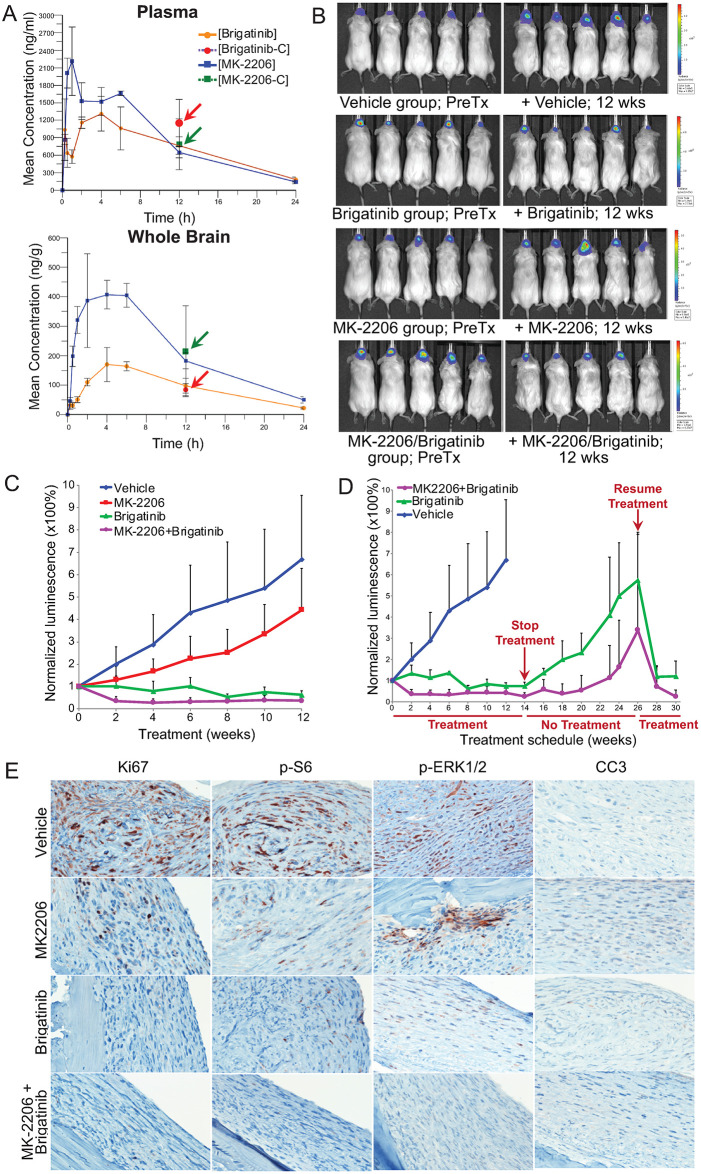
Brigatinib and its combination with MK-2206 effectively shrank intracranial meningioma xenografts. (A) Pharmacokinetic (PK) analysis was conducted to determine the concentrations of brigatinib and MK-2206 in mouse plasma and brain. Mice were treated with a single maximum tolerated dose (MTD) of brigatinib or MK-2206. Prior to and at various times after dosing (n = 3 per time point), blood and brain from each dosed mouse were collected for UHPLC-MS/MS. The mean concentration of each drug at each indicated time point with standard deviation was plotted. Also, mice were treated with a combined dose of brigatinib and MK-2206 at their MTD (n = 3), and the drug concentration in the plasma and brain determined (indicated with arrows). (B-C) Mice with established meningioma xenografts were treated with vehicle, brigatinib, MK-2206, or brigatinib+MK-2206 by oral gavage (n = 10 each) and tumor growth was monitored by BLI. (B) Shown are representative BL images of tumor-bearing mice acquired prior to (PreTx) and 12 weeks (wks) after treatment. (C) The relative tumor-emitted BL signals were quantified and denoted as % of total flux after treatment relative to the total flux prior to treatment designated as one (100%). The data are shown as mean ± standard deviation. At least seven mice from each group completed the entire 12-week treatment. (D) Upon cessation of treatment, tumors in mice that had been treated with brigatinib or brigatinib+MK-2206 for 14 weeks (n = 4 each) regrew. However, tumor shrinkage was observed when the treatment was re-initiated. Data shown for mice treated with brigatinib or brigatinib+MK-2206 are only from the cage of mice that had undergone cessation of treatment and retreatment. (E) Representative images of immunostained sections of the heads of tumor-bearing mice after 12-week treatment with vehicle, MK-2206, brigatinib, or MK-2206+brigatinib for Ki67, p-S6, p-ERKs, and cleaved caspase 3 (CC3) expression.

Patients with NF2 will often suffer from both meningiomas and schwannomas. Given the *in vitro* efficacy and the -omics supported mechanism of action in both tumor cell models, we chose to test brigatinib both alone and in combination with MK-2206 in a tumor xenograft model of meningioma. Mice were first injected with the human *NF2*-deficient Ben-Men-1 meningioma cells and then treated with either 120 mg/kg/qd MK-2206, 50 mg/kg/qd brigatinib, or the combination. Peak serum concentrations of brigatinib reached the 1.2μM concentration shown to be the IC50 of these cells *in vitro* with drug concentrations in the brain lagging by a little less than one order of magnitude (Figs 2C and 5A). Combination therapy increased concentrations of brigatinib in the serum but not in the CNS compared with treatment of brigatinib alone (Figs 5A and S11). As measured by luminescence engineered into the meningioma cells, brigatinib, both alone and in combination with MK-2206 blocked outgrowth of the xenograft (Figs 5B, 5C and S12).

To assess whether brigatinib and/or MK-2206 were tumoristatic or tumoricidal, treatment was halted after 14 weeks of therapy. Cessation of treatment was associated with rapid xenograft expansion (Fig 5D). However, treatment re-initiation resulted in rapid loss of luminescence, indicating that the xenograft was still drug sensitive (Fig 5D). Immunohistochemistry showed a significant reduction in tumor cell proliferation consistent with luminescence signal and proliferative signaling in treated xenografts (Fig 5E).

**Fig 6:**
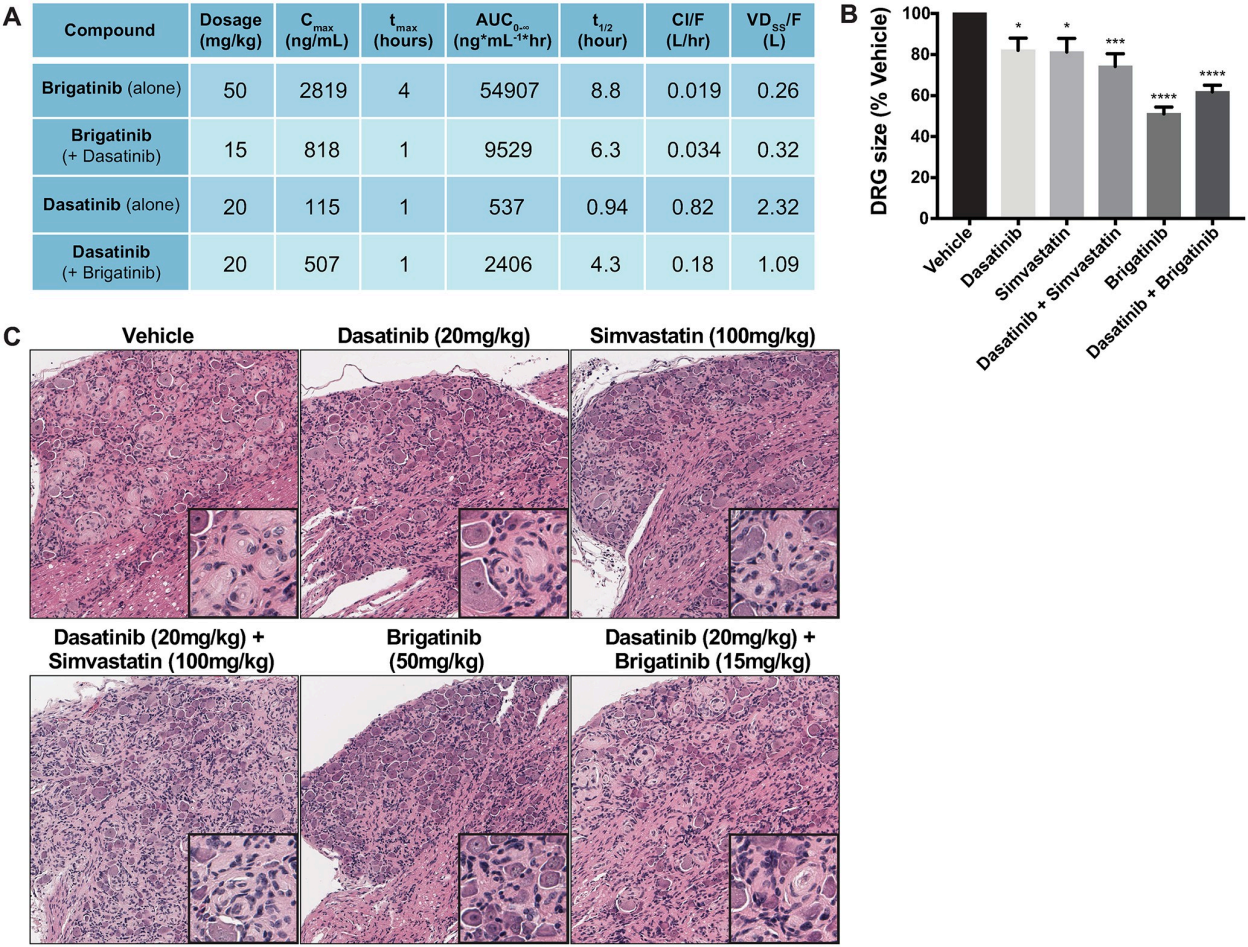
Brigatinib, dasatinib, simvastatin and combination treatments in the schwannoma mouse model. (A) Single-dose pharmacokinetics of compounds in *PostnCre; Nf2*^*flox/flox*^ mice. Plasma compound concentrations were measured at baseline, 1, 2, 4, 8 and 24 hours after administering a single oral dose to n = 3 *PostnCre; Nf2*^*flox/flox*^ mice. Parameters were calculated using a noncompartmental NCA-xls using PK add ins. (B) Volume of dorsal root ganglia (DRG) was measured post-mortem. All treatment groups had significantly smaller DRG volume when compared to vehicle, and brigatinib-treated DRGs showed the greatest reduction. One-Way ANOVA with Dunnett’s multiple comparison’s test; p = 0.0001(****), p = 0.0007(***), p<0.05(*). (C) Representative hematoxylin and eosin stained DRGs following 12 weeks of continuous treatment with the indicated drugs.

In parallel *in vivo* studies in schwannomas, *Postn-Cre*;*Nf2*^*flox/flox*^ mice were treated for 12 weeks with brigatinib and dasatinib, as single agents and in combination as well as dasatinib in combination with simvastatin, another FDA approved therapeutic which demonstrated synergy with dasatinib in the drug screens (S4 Fig). Mice treated with brigatinib alone received the maximum tolerated dose of 50 mg/kg/qd. Due to *in vivo* toxicity in the preclinical model, the dose of brigatinib in the brigatinib and dasatinib combination was reduced to 15 mg/kg/qd. The combination of the lower dosing and decreased half-life of brigatinib in the combination therapy group significantly reduced total drug exposure as measured by AUC (Fig 6A). While serum concentrations of brigatinib in the brigatinib alone treated mice came very close to the previously determined *in vitro* IC50 of MS02 cells, serum concentrations of brigatinib in the combination therapy group were much lower, in a range which we would predict to be less therapeutically efficacious given our *in vitro* data (Figs 2G and 6A). Brigatinib alone significantly reduced the volume of tumor-bearing dorsal root ganglion (DRG) tissue (Fig 6B). Finally, tissues from mice treated with brigatinib had some Schwann cell hypercellularity at the end of 12 weeks of treatment, but had far fewer areas of discrete schwannoma when compared to the other treatment conditions (Fig 6C). The brigatinib and dasatinib combination therapy also reduced the DRG volume, but by a smaller magnitude.

## Discussion

In these studies, we undertook an unbiased, systems based approach to ultimately identify brigatinib as a novel monotherapy for the treatment of *NF2* related schwannomas and meningiomas. This work began with a series of cell based drug screens and utilized the ~2000 compounds contained in the MIPE 4.0 oncology collection. ALK-IN-1 was identified as a potent and selective inhibitor of *NF2*-deficient arachnoid and Schwann cell lines compared with otherwise isogenically matched *NF2/Nf2*-expressing cells. We chose not to pursue ALK-IN-1 directly as a therapeutic because ALK-IN-1 was originally generated as a part of a series of structural analogs which could inhibit ALK. Ultimately therapeutic development of that series of molecules favored a different analog, AP26113, now known as brigatinib. We confirmed that brigatinib had efficacy comparable to ALK-IN-1 in slowing the growth of schwannoma and meningioma cells lines and also exhibited modest *in vitro* synergy in growth inhibition of meningioma cells when combined with MK-2206 (Figs 2B–2D and S2). Hence, brigatinib replaced ALK-IN-1 for the *in vivo* studies.

The reasoning behind relying on an unbiased, systems based screen to identify novel therapeutics for NF2 was driven in part by our incomplete understanding of the biochemical functions of Merlin which has hampered prior efforts to advance therapeutics for this disease. Indeed, we would not have anticipated a drug known for its ability to inhibit ALK demonstrating preclinical efficacy in either *NF2* associated schwannoma or meningioma. Given this observation, we were surprised that ALK is not expressed in either tumorigenic cell type (Figs 4A, S7, S8 and S10). Following up on this unexpected finding, we demonstrated that in our particular cell culture conditions and in the primary tumors *in vivo*, the kinases most inhibited by brigatinib were non-RTKs (FER, PTK2B/FAK2, and PTK2/FAK) and serine/threonine kinases (TNK2, STK33, and GAK). Similarly, the non-RTK PTK2/FAK and the serine-threonine kinases GAK, RPS6KA3/RSK2, and MARK3 were also found to be greatly suppressed in brigatinib-treated schwannoma cells (Fig 3). Our findings underscore the importance of kinome profiling in disease models of interest to identify potentially relevant but unanticipated drug targets [19].

From the New Drug Application [15] information of ALUNBRIG^™^ submitted to the FDA, we learned that while brigatinib has an IC_50_ of ~1 nM against ALK, it potently inhibits at relatively low IC_50_ values (≤ 10 nM) other RTKs, like ROS1 and FLT3, several non-RTKs, including FER, FES/FPS, FAK/PTK2, and breast tumor kinase/protein tyrosine kinase 6 (BRK/PTK6), as well as the serine/threonine kinases STK22D and checkpoint kinase 2 (CHK2). Brigatinib is also active at IC50 ≤ 100 nM against several RTKs, including the various EGFR-family members, vascular endothelial growth factor receptor 3 (VEGFR3), RET, and IGF-1R, non-RTKs, such as YES and PTK2B/FAK2, and a number of serine/threonine kinases. In addition, brigatinib inhibits various fibroblast growth factor receptors (FGFRs), several EPH receptors, and non-RTKs at low micromolar concentrations that have been achieved clinically by many other RTK inhibitors. Using a proteome-wide drug screening approach, it was confirmed that brigatinib strongly interacts with ALK, PTK2/FAK1, and PTK2B/FAK2 and has a medium affinity to IGF-1R and EGFR [20]. These results suggest that the non-RTKs FAK/PTK2 and FER, which are strongly inhibited by brigatinib in *NF2*-deficient meningioma and schwannoma cells, are important for the growth and survival of these tumors. However, the multiple other targets of brigatinib in signaling pathways linked to Merlin may also impact the therapeutic efficacy of this drug.

It should be emphasized that even though we did not detect changes in the RTKs that are frequently activated in *NF2*-related tumors by MIB/MS, we were able to demonstrate suppression of phosphorylated EGFR, ErbB3, and IGF-1R in cells treated with 1x IC_50_ concentration of brigatinib under ligand-induced growth conditions (Fig 3B). At this brigatinib concentration, we also detected inhibition of EphA2, which we previously showed increased expression and activation of this RTK in *NF2*-deficient cells [21]. Consequently, activation of all these RTKs and non-RTKs leads to downstream activation of both PI3K-AKT and MEK-ERK signaling (Fig 3). Collectively, our results suggest that the anti-tumor activity of brigatinib in *NF2* associated schwannoma and meningioma is mediated through blockade of multiple RTKs and non-RTKs.

Consistent with previous reports [13,22], brigatinib and MK-2206 exhibited an overall favorable pharmacokinetic (PK) profiles. In the plasma, brigatinib reached a maximum of 2.2 μM when given orally (1,311 ng/ml of C_max_ in S11 Fig), which is above the IC_50_ in Ben-Men-1 cells. Our PK data also showed effective blood-brain penetration and further indicates no drug-drug interaction between brigatinib and MK-2206 (Fig 5A). These results should guide the dosing regimen for these drugs in a future clinical trial. Importantly, brigatinib monotherapy induced tumor regression in meningioma and its combination with MK-2206 resulted in further tumor shrinkage (Fig 5B and 5C). Although meningiomas regrew upon cessation of treatment, these tumors remained responsive to brigatinib, either alone or in combination with MK-2206 (Fig 5D). These results suggest that it is possible to have short breaks from treatment while maintaining therapeutic responses to retreatment, particularly after treatment with the brigatinib/MK-2206 combination.

Brigatinib as a single agent was well-tolerated and showed efficacy in the *NF2* GEMM animals. We observed a nearly 40% reduction in average DRG volume after 12 weeks of daily brigatinib treatment (Fig 6B) and reduced discrete schwannoma formation in the brigatinib-treated DRGs (Fig 6C). The two combinatorial therapeutics of brigatinib/dasatinib and dasatinib/simvastatin which were identified in the drug screen and tested *in vivo* were also efficacious in reducing schwannoma growth albeit to a lesser extent than what was observed with brigatinib alone at a higher concentration. Of note, significant toxicity was observed in the brigatinib/dasatinib treatment group. Due to toxicity, the dose of brigatinib in the brigatinib/dasatinib combination had to be reduced from 50 mg/kg to 15 mg/kg. The lower dose of brigatinib in the combination therapy group significantly reduced total drug exposure as measured by AUC (Fig 6A). Therefore we believe the reduction in efficacy of the brigatinib/dasatinib combination therapy over the brigatinib single agent treatment may be a result of the greater than threefold reduction in brigatinib dosing given to the combination treated mice. The magnitude of the reduction in average DRG volume observed in the Brigatinib treated mice is the largest we have seen for any treatment in the *Postn-Cre*;*Nf2*^*flox/flox*^ animals. DRG tissue harvested from mice at the end of the 12-week treatment period were evaluated by western blot and showed that brigatinib alone and brigatinib + dasatinib combination treatments resulted in greater inhibition of p-FAK, p-AKT and p-S6 pathways when compared to the other treatment groups (S13 Fig). Interestingly, dasatinib alone did not result in a significant inhibition of these kinases, also supporting the importance of brigatinib *in vivo* in the brigatinib + dasatinib combination group.

The studies executed here rely at least in part on xenograft and genetically engineered murine models. Consequently, we acknowledge that there could be important species-specific biological features of our studies compared to those in humans. However, in a closely related disease, Neurofibromatosis Type 1, we and others have found that genetically engineered murine models closely replicate the tumor pathogenesis and offer insights into a meaningful clinical signature in patients [23] for two distinctly different drugs [23–25]. Furthermore, the omics methodology utilized in one of these recent studies [25] was also key to identify specific activated kinases that may be important in tumor progression. The same methodologies were used in these studies. Finally, the kinases that were identified using MIB/MS were independently validated using other methodologies. Based on the data presented here, brigatinib possesses potent anti-tumor activity against *NF2*-deficient tumors via inhibition of multiple RTKs frequently activated in these tumors as well as blockade of several non-RTKs and serine-threonine kinases, but not ALK. Given the fact that brigatinib caused tumor shrinkage in both *NF2* associated schwannoma and meningioma, a clinical trial was initiated to assess the therapeutic effectiveness of brigatinib in NF2-associated tumors (ClinicalTrials.gov: NCT04374305).

## Materials and methods

### Compounds and library

The MIPE (Mechanism Interrogation Plates) oncology compound library 4.0 consisting of 745 FDA-approved drugs, 420 Phase I-III investigational agents, and 767 preclinical molecules was screened at the National Center for Advancing Translational Sciences (NCATS), National Institutes of Health. All compounds in the library have been annotated for their known nominal target, representing diverse and redundant mechanisms of action. Also, the library contains several compounds per target, allowing target-based enrichment analysis of responses; however, most compounds have polypharmacology. See Supplemental Methods in S1 File for additional details.

### Cells and compound screening

A panel of NF2 tumor-relevant cells (Schwann cells for schwannoma and arachnoidal cells for meningioma with *NF2*-expressing or *NF2*-deficient) was previously assembled for systematic drug evaluation [1]. In this study, we used the isogenic pairs of *NF2*-expressing HS11 and *NF2*-deficient HS01 Schwann cells and *NF2*-expressing Syn1 and *NF2*-null Syn5 arachnoidal cells [26,27]. Also, human *NF2*-deficient meningioma Ben-Men-1, mouse *Postn-Cre*; *Nf2*^*flox/flox*^ schwannoma MS02, and primary human meningioma and schwannoma cells were used [28,29]. All cells were grown and tested for mycoplasma contamination as previously described [1].

For compound screening, actively-growing HS11 and HS01 Schwann cells and Syn1 and Syn5 arachnoidal cells as well as Ben-Men-1 cells were seeded in white 1536-well plates (Corning, NY) at a density of 750 cells/well using a Multidrop Combi Reagent dispenser with a small pin cassette (ThermoFisher, Fair Lawn, NJ). The next day, cells were treated for 72 hours with 11 two-fold serial dilutions of each compound in the MIPE 4.0 library. Cell viability was measured using CellTiter-Glo (Promega, Madison, WI). The potency (IC_50_) and efficacy (maximum % inhibition) of each compound in each cell line were calculated as described [10].

HEK293T cells used for NanoBRET assays were cultured in Dulbecco’s Modified Eagle Medium supplemented with 10% fetal bovine serum and penicillin-streptomycin. Cells were obtained from ATCC and routinely tested for mycoplasma contamination by DAPI staining.

### *In vitro* confirmation of compound efficacy and synergism

Meningioma-relevant cells, including Syn1, Syn5, Ben-Men-1, and primary cultures of *NF2*-null meningioma cells, were seeded in 384-well plates and treated the next day with serial dilutions of brigatinib, MK-2206, or the combination of MK-2206 with brigatinib in a 10x10 matrix format. Cells were treated with 0.1% DMSO as the vehicle control. Cell viability was measured after 72 h of treatment using CellTiter-Glo. DRCs were generated using GraphPad Prism 7.0 (www.graphpad.com).

For the schwannoma-relevant cell panel, HS01, HS11, and MS02 cells were seeded in 384-well plates overnight and incubated with various dilutions of ALK-IN-1, brigatinib, dasatinib, or the combination of dasatinib with ALK-IN-1 or brigatinib in the 10x10 matrix format. Cell viability was measured after 60 or 72 h of treatment for mouse MS02 or human HS01 and HS11 cells, respectively, using CellTiter-Fluor (Promega). A similar study to validate the efficacy of dasatinib, simvastatin, and their combination was also conducted.

For both cell types, dose-response measurements were normalized to vehicle controls and dose-response curves were generated. Drug combination synergy was analyzed using the Loewe, Bliss, and HSA models as previously described using the Combenefit sotware [30].

### RNA isolation and transcriptome analysis

RNA was isolated from all meningioma- and schwannoma-relevant cell lines treated in triplicate with single and combination drugs and used to prepare RNA libraries for RNAseq using Illumina HiSeq2000 and 2500 platforms [1]. Pairwise-compared, differentially-expressed genes were identified and gene ontology enrichment analysis for each comparison was conducted as described in Supplementary Methods in S1 File.

### Kinome profiling

Multiplexed kinase inhibitor bead affinity chromatography and mass spectrometry (MIB/MS) was conducted to identify and quantitate the activity of the expressed kinome and their responses to drug treatment as previously described [1,17,31]. Lysates prepared from cells treated with the indicated concentrations of each drug or drug combination for various times or from tissues from animals treated with the indicated drugs were prepared and loaded onto the column containing multiple inhibitor-conjugated beads [31]. Kinase-bound inhibitor beads were stringently washed, followed by protein purification and trypsin digestion. Liquid chromatography, mass spectrometry, and analysis was essentially as previously described [31]. Peptide suspension was separated using an EASY nLC-1000 System with an Easy-Spray C-18 column (Thermo Fisher). Raw files were processed for label-free quantification using MaxQuant LFQ and default parameters with the following modifications—razor plus unique peptides were used, matching between runs (3 min match time window), fixed modifications [cardbidomethy (C)] and dynamic modifications [phospho(STY)] [32]. Kinase LFQ intensities were used if two or more unique peptides were detected, and missing values were imputed in Perseus if observed in all replicates of another condition after log2 transformation for comparison. Data for each treated sample was plotted as a mean of fold change (log_2_) relative to DMSO- or vehicle-treated control or untreated animals. When at least three replicates were available, unpaired Student’s t-tests were performed in Perseus with Benjamini-Hochberg correction. FDR of 5% was used as a cut-off for significance.

### NanoBRET^™^ TE intracellular kinase assays

NanoLuc fusion vectors were purchased from Promega Corporation (PTK2, NV1921; FER, NV1331; GAK, NV1421; and TNK2, NV4451). The Promega Kinase assay kit with K-10 tracer (N2641) was purchased and used for all kinase vectors. The optimized protocol from Promega was employed, with the following modifications. HEK293T cells were plated at a density of 20,000 cells per well in white 96-well plates in assay medium (Opti-MEM Reduced Serum Medium with no phenol red (Life technologies Cat.#11058–021) containing 1% fetal bovine serum). Four hours after plating, cells were transfected with 0.1 micrograms total DNA per well (9 parts carrier DNA: 1 part NanoLuc kinase fusion vestor) using jetPRIME transfection reagent (Polyplus) at a 2:1 ratio (microliters jetPRIME to micrograms DNA). Twenty hours later, the cells were treated with tracer according to protocol and serial dilutions of brigatinib or vehicle (ethanol) for 2 hours at 37 C, 5% CO_2_. Substrate and inhibitor solutions were prepared according to protocol and incubated for 3–5 min before reading on a Synergy H4 (BioTek) plate reader using a 450/50 nm emission filter (BioTek Part no. 7082208) and a long-pass 610 nm filter (BioTek Part no. 7092209) with an integration time of 1 sec. BRET ratios were exported and multiplied by 1,000 to convert to NanoBRET ratios in milliBRET units (mBU), without background correction, according to Promega protocol. Values were plotted in GraphPad Prism 8 and the best-fit IC50 values were obtained by nonlinear regression fit using the log(inhibitor) vs. response–variable slope (four parameter) function in Prism. The experiments were performed at least twice, and representative data are shown.

### Quantifiable orthotopic meningioma model

All animal studies conducted at Nationwide Children’s Hospital were conducted in accordance with the Institutional Animal Care and Use Committee under the approved protocol number AR09-00054. Mice were housed in the barrier facility within the vivarium in our Abigail Wexner Research Institute with HEPA (high-efficient particulate air)-filtered room air, temperature control at 72°F, and humidity set at 40–60%. Environmental parameters within each animal room were monitored electronically 24/7. The mouse rooms were equipped with micro-isolator caging on ventilated racks and class II biological safety cabinets. Each polycarbonate cage (30cm x 18cm x 15cm) housed a maximum of 5 mice and contained corn cob bedding and a polycarbonate hut or tube for hiding and climbing or a LifeSpan^™^ hanging devices for additional floor space above the cage bottom. Mice were fed non-fat rodent chow and filtered or sterile drinking water, and cages changed every week. All animals were euthanized by CO_2_ asphyxiation, followed by cervical dislocation according to the approved IACUC protocol.

For animal dosing, MK-2206 was dissolved in 30% Captisol^®^ and brigatinib was formulated in 90% polyethylene glycol 300 and 10% 1-methyl-2-pyrrolidinone. The MTD was determined as described (Plowman et al., 1999). Eight-to-12 week-old NSG (NOD-SCID gamma or *NOD*.*Cg-Prkdc*^*scid*^ *Il2rg*^*tmlWjl*^*/SzJ*) mice (The Jackson Laboratory) were treated with various doses of MK-2206 every other day or brigatinib every day by oral gavage for two weeks (n = 3 per dose). Treated mice were monitored daily for any signs of distress, such as significant weight loss (>20%), lethargy, and dehydration. Following MTD determination for each drug, a combination of MK-2206 and brigatinib at the MTD was also evaluated to ensure tolerability.

For PK analysis, NSG mice were fed a single oral dose of brigatinib, MK-2206, or their combination at the MTD. Prior to and at various times after dosing (n = 3 per time point), whole blood was collected from the facial vein into EDTA-containing Microtainer^®^ (Becton Dickinson). Plasma samples were obtained by centrifugation. Immediately after blood sampling, mice were euthanized for brain harvesting. Both the plasma and brain samples were frozen at -80°C until analysis of compound concentrations using ultra-high pressure liquid chromatography tandem-mass spectrometry (UHPLC-MS/MS). Plasma and brain samples were deproteinized and were analyzed in the presence of the brigatinib analogue ALK-IN-1 and dasatinib-d_8_ as the internal standards for brigatinib and MK-2206, respectively.

To generate the orthotopic meningioma model, Ben-Men-1-LucB cells into the skull base of NSG mice as previously described [28]. Injected mice were monitored daily for any significant weight loss (>20%), lethargy, dehydration, and neurological problems. Mice with any of these abnormalities would have been euthanized, followed by a complete histopathological examination, however no mice met the criteria for early euthanasia in this study. In addition, we monitored tumor growth in mice by bioluminescence imaging (BLI) every two weeks using a Xenogen IVIS^®^ Spectrum imaging system (Perkin Elmer). Mice with successful tumor engraftment were randomized into four groups (n = 10 each) and treated with MK-2206, brigatinib, MK-2206/brigatinib combination, or a mixture of the corresponding vehicles in which MK-2206 and brigatinib were formulated (1:1 ratio) by oral gavage. BLI was performed biweekly to assess the effects on tumor growth. The luminescence detected in each mouse was normalized to its pretreatment signal and expressed as the mean normalized luminescence ± standard deviation for each treatment group [28]. After 14 weeks of treatment, we stopped treating a cage of mice that had received brigatinib or the MK-2206/brigatinib combination and monitored possible tumor regrowth for 12 more weeks. Then, we retreated these mice to examine the effects of retreatment. All mice were humanely euthanized at the conclusion of the studies and tissues were collected for histopathological assessment.

### Schwannoma model

All animal studies conducted at Indiana University were in accordance with the Institutional Animal Care and Use Committee under protocol number 11406. The methodologies of tumor assessment for the *PostnCre; Nf2*^*flox/flox*^ mice were used as previously described [33]. Drugs were formulated as follows and administered by oral gavage: 50 mg/kg brigatinib dissolved in 90% PEG400 with 10% 1-metyl-2-pyrrolidinone; 20mg/kg dasatinib in pH3.0 citrate buffer; and 100 mg/kg simvastatin in 1% carboxymethylcellulosein water with 0.25% Tween80 and 0.05% antifoam 204 (Sigma). Cohorts of *Nf2*^*f/f*^*; Postn-Cre*+ mice were generated in-house, as previously described [33]. Animals were monitored daily for signs of distress such as hunched posture, labored breathing, scruffy coat, diarrhea, or lameness. All study mice began treatment at 4 months of age and were euthanized at the end of 12 weeks of treatment for histopathological assessment. Early endpoint criteria include: a palpable mass of more than of 1,000mm^3^, greater than 20% reduction in body weight, any of the previously mentioned signs of distress that could not be remedied by the veterinary staff, or at the recommendation of the veterinarian. The total animals were enrolled in each treatment group are as follows: vehicle n = 17, brigatinib n = 17, simvastatin n = 16, dasatinib n = 15, dasatinib and simvastatin n = 15 and dasatinib and brigatinib n = 16. The number of animals that met the criteria for euthanasia and were humanely euthanized according to the IACUC protocol or were found deceased prior to meeting criteria for euthanasia were as follows: vehicle, n = 1, brigatinib, n = 7, simvastatin n = 2, dasatinib n = 1, dasatinib and simvastatin n = 1, and dasatinib and brigatinib n = 4.

Tissue processing and DRG volume quantification methods were carried out as previously described [33]. Dissected nerve trees were pre-embedded in 2% agar, processed in a Leica tissue processor through graded alcohols, xylenes, and finally in molten paraffin. Five-micron thick sections were cut on a Leica rotary microtome and mounted on charged slides, then stained with hematoxylin and eosin. Images were acquired with an Aperio CS2 slide scanner (Leica).

HPLC/MS was performed by the IU Simon Cancer Center’s Clinical Pharmacology Analytical Core. Six mice per drug dosage and three mice for vehicle only were used for pharmacokinetic experiments. Mice were given a single dose of drug and then blood was collected 1 and 4 hours later for one set of 3 mice and 2, 8, and 24 hours later for a second set of 3 mice. The blood was centrifuged at 4°C, and then plasma was collected and stored at -80°C. Samples were acidified and extracted in hexane:ethyl acetate (50:50, v/v). After solvent evaporation, mobile phase (acetonitrile:5mM ammonium acetate; 70:30, v/v) was mixed with residual sample and injected into an Agilent 1290 HPLC system with an Eskigent Autosampler. Mass spectrometry was performed using an ABSciex 5500 Q-TRAP.

## Supporting information

S1 Fig Cell survival responses to all MIPE 4.0 library agents (as judged by relative AUCs) binned per mechanistic classes with mechanistic superclasses listed in order from top to bottom: Transcriptional regulation, physiological homeostasis, other, metabolism, DNA repair, cell surface protein, cell signaling, cell growth, antimicrobial. Solid dots represent the median response for all the compounds, in each mechanistic class, and for each cell line.
(PDF)

S2 Fig Single and combination drug treatment of meningioma-related cell lines. Drug treatment of the *NF2*-expressing Syn1(+) and *NF2*-null Syn5(-) cells (A), Ben-Men-1 cells (B), and two primary meningioma cultures MN612 and MN621 (C) was performed using the same drug doses as for Fig 2C and 2D. Single drug DRCs and combination drug heatmaps were generated using Combenefit software. Note that heatmap data for Ben-Men-1 (B) is the same as shown in Fig 2D.
(PDF)

S3 Fig Brigatinib and dasatinib synergized to reduce *NF2*-deficient Schwann cell viability. (A) *Nf2*^-/-^ mouse MS02 schwannoma and *NF2*-deficient human HS01 Schwann cells were plated and tested in dose-response 10x10 matrix format as described in Methods. Shown are single-agent dose response curves for ALK-IN-1 and brigatinib. (B) Brigatinib/dasatinib combination matrix analyses were conducted with HS01 cells. Shown are the brown-scale viability and synergy matrix plots with modeled surface synergy distributions in Loewe, Bliss, and HSA models. (C) Brigatinib/dasatinib combination matrix were analyzed with MS02 cells as described in S3B Fig.
(PDF)

S4 Fig Dasatinib and simvastatin synergized to reduce the viability of Schwann and schwannoma cells. 10x10 matrix dose-response analysis was performed for *NF2*-deficient HS01 (A) and *NF2*-expressing HS11 (B) human Schwann cells and *Nf2*^*-/-*^ mouse schwannoma MS02 cells (C) treated with the brigatinib/simvastatin combination. Shown are the viability (brown scale) and synergy matrix (blue to red scale) plots with modeled surface synergy distributions in Loewe, Bliss, and HSA models.
(PDF)

S5 Fig Gene expression changes caused by drug treatments. (A) Significant (Bonferroni-adjusted p < 0.05) changes in gene expression in Syn5 and Syn1 arachnoidal cells, which show extensive overlap with an overall greater response in the Syn5 merlin-null cells, are caused by brigatinib or brigatinib/MK-2206 treatment, but not by MK-2206 alone. (B) Genes whose expression is altered both by lack of merlin in Syn5 and by treatment of Syn5 with either brigatinib or brigatinib/MK-2206 treatment overwhelmingly show the expression change in opposite directions. (C) Significant changes in gene expression in HS01 and HS11 Schwann cells are limited in single drug treatments and most pronounced with the brigatinib/dasatinib combination. (D) The small number of genes differentially expressed in HS01 as a result of merlin expression do not overlap extensively with those altered in HS01 by the brigatinib/dasatinib treatment, and those overlaps that do occur are concordant in direction, contrasting with the arachnoidal cells.
(PDF)

S6 Fig MIB/MS kinome profiling reveals dasatinib and brigatinib target specificity. (A) Control sciatic nerve tissues from *Postn-Cre; Nf2*^*flox/flox*^ mice were used to prepare lysates. Equal amount of protein lysates (1mg) was incubated with vehicle (0.001% ethanol) or brigatinib (10nM, blue; 100nM, red; 1000nM, green) for 2h in biological duplicate. The log2 difference in MIB binding (LFQ intensity) was calculated and plotted relative to vehicle for each replicate. (B) HS11 and HS01 cells were treated with vehicle or brigatinib (1μM) for 24h prior to MIB/MS kinome profiling. MIB binding (log2LFQ intensities) was determined and PCA performed in Perseus. (C) HS11 and HS01 cells were treated with vehicle or dasatinib (0.6μM) for 24h prior to MIB/MS kinome profiling. MIB binding (log2LFQ intensities) was determined and the top 20 kinases with decreased MIB binding compared to vehicle are shown as a stacked bar plot for the two cell lines. (D-G). NanoBRET target engagement intracellular kinase assays were performed using the indicated kinases (PTK2/FAK1, FER, GAK, and TNK2) expressed as NanoLuc fusion constructs in HEK293T cells. Cells were treated with the indicated concentration of vehicle or brigatinib for 2 h prior to reading the plates. The 450/50 nm and long-pass 610 nm emission values were used to determine the NanoBRET^™^ ratios. Values were plotted in Prism and the best-fit IC50 values are indicated. Representative data is shown from experiments performed at least twice.
(PDF)

S7 Fig ALK was not detected in normal meningeal and meningioma cells with or without *NF2* expression. Equal amounts of protein lysates from three human meningioma cell lines (KT21-MG1-Luc, NF2-Men-1, and Ben-Men-1), normal meningeal cells, and four primary meningioma cell cultures (hMen-10A, hMen-10B, hMen-12Ao, hMen-12B) were used in Western blot analysis to probe ALK and merlin expression. SK-N-SH neuroblastoma cells, which express ALK, were used as a positive control.
(PDF)

S8 Fig Inhibition of multiple RTKs and non-RTKs in Ben-Men-1 cells treated with 1x IC_50_ of brigatinib under various growth conditions. PathScan^®^ RTK signaling antibody array analysis was conducted according to Supplementary Methods in S1 File using cells grown in 10% FBS and treated with 1x IC_50_ of brigatinib for 2h (A) and 24 h (B) or in growth-arrested cells stimulated with 20% FBS in the presence of 1x IC_50_ of brigatinib for 2h (C) and 24 h (D). The numeric table below each pair of arrays displays the fold-change in fluorescence detected for each phospho-protein expressed in cells treated with brigatinib relative to DMSO control after subtraction of the fluorescence in the background control spots (indicated as “-”). Positive control spots are denoted as “+”.
(PDF)

S9 Fig Ben-Men-1 cells expressed low levels of p-EGFR, which was abolished by brigatinib treatment. Western blot analysis revealed that while phosphorylated EGFR was robustly induced in EGF-stimulated Ben-Men-1 cells, only very low levels of p-EGFR were detected in actively-growing cells or growth-arrested cells stimulated with 20% serum for 24h. Brigatinib treatment diminished p-EGFR expression.
(PDF)

S10 Fig ALK expression was not detected in Schwann and schwannoma cells. (A) Western blotting was performed to detect ALK expression in human Schwann cells, four primary vestibular schwannoma cultures (hVS-16H, hVS-16S, hVS-12J, hVS-12Ga), and SK-N-SH neuroblastoma cells. Tubulin was used as a loading control. (B) Western blotting for ALK was also performed using extracts from nine vestibular schwannoma tumors. GAPDH was used as a loading control.
(PDF)

S11 Fig Mouse PK parameters following a single oral dose of brigatinib or MK-2206 at the MTD. Mice were orally fed 50 mg/kg of brigatinib or 120 mg/kg of MK-2206. PK analysis was performed according to Supplementary Methods in S1 File.
(PDF)

S12 Fig Relative tumor sizes in meningioma-bearing mice prior to or after treatment with MK-2206 and brigatinib, either alone or in combination, for 12 weeks. Detailed drug treatment and tumor measurement were as described in Fig 5B and 5C and Supplementary Methods in S1 File.
(PDF)

S13 Fig Brigatinib inhibited p-FAK, p-AKT, and p-S6 target pathways in *Postn-Cre;Nf2*^*flox/flox*^ mice. DRGs from *Postn-Cre;Nf2*^*flox/flox*^ mice treated with the indicated drug or drug combination were used in Western blotting as described in Supplementary Methods in S1 File. Mice were treated for 12 weeks with the following drug dosages: 20mg/kg dasatinib, 50mg/kg brigatinib, 20mg/kg dasatinib + 15mg/kg brigatinib, 100mg/kg simvastatin, and 20mg/kg dasatinib + 100mg/kg simvastatin. DRG tissues were harvested 2 or 24 hours after the last dose of drug.
(PDF)

S1 Table Fold-change and significance of differential expression in treated and untreated cells. (XLSX)

S2 Table Pathways enriched for differentially expressed genes. (XLSX)

S3 Table Kinome data plotted in Fig 3A–3D. (XLSX)

S4 Table Kinome data plotted in Fig 3E. (XLSX)

S5 Table Kinome data plotted in Figs 3F and S6A. (XLSX)

S6 Table Kinome data plotted in Fig 3G. (XLSX)

S7 Table Kinome data plotted in Figs 3H and S6B. (XLSX)

S1 File Supporting information methods. (DOCX)

S1 Raw images (PDF)

## References

[1] Synodos for NFC, Allaway R, Angus SP, Beauchamp RL, Blakeley JO, Bott M, et al. Traditional and systems biology based drug discovery for the rare tumor syndrome neurofibromatosis type 2. PLoS One. 2018;13(6):e0197350. doi: 10.1371/journal.pone.0197350 29897904 PMC5999111

[2] Blakeley JO, Evans DG, Adler J, Brackmann D, Chen R, Ferner RE, et al. Consensus recommendations for current treatments and accelerating clinical trials for patients with neurofibromatosis type 2. Am J Med Genet A. 2012;158A(1):24–41. doi: 10.1002/ajmg.a.34359 22140088 PMC3319201

[3] Evans DG, Kalamarides M, Hunter-Schaedle K, Blakeley J, Allen J, Babovic-Vuskanovic D, et al. Consensus recommendations to accelerate clinical trials for neurofibromatosis type 2. Clin Cancer Res. 2009;15(16):5032–9. doi: 10.1158/1078-0432.CCR-08-3011 19671848 PMC4513640

[4] Cui Y, Groth S, Troutman S, Carlstedt A, Sperka T, Riecken LB, et al. The NF2 tumor suppressor merlin interacts with Ras and RasGAP, which may modulate Ras signaling. Oncogene. 2019;38(36):6370–81. doi: 10.1038/s41388-019-0883-6 31312020 PMC6756068

[5] Hadfield KD, Smith MJ, Urquhart JE, Wallace AJ, Bowers NL, King AT, et al. Rates of loss of heterozygosity and mitotic recombination in NF2 schwannomas, sporadic vestibular schwannomas and schwannomatosis schwannomas. Oncogene. 2010;29(47):6216–21. doi: 10.1038/onc.2010.363 20729918

[6] Ruttledge MH, Sarrazin J, Rangaratnam S, Phelan CM, Twist E, Merel P, et al. Evidence for the complete inactivation of the NF2 gene in the majority of sporadic meningiomas. Nat Genet. 1994;6(2):180–4. doi: 10.1038/ng0294-180 8162072

[7] Ammoun S, Hanemann CO. Emerging therapeutic targets in schwannomas and other merlin-deficient tumors. Nat Rev Neurol. 2011;7(7):392–9. doi: 10.1038/nrneurol.2011.82 21647202

[8] Blakeley JO, Plotkin SR. Therapeutic advances for the tumors associated with neurofibromatosis type 1, type 2, and schwannomatosis. Neuro Oncol. 2016;18(5):624–38. doi: 10.1093/neuonc/nov200 26851632 PMC4827037

[9] Troutman S, Moleirinho S, Kota S, Nettles K, Fallahi M, Johnson GL, et al. Crizotinib inhibits NF2-associated schwannoma through inhibition of focal adhesion kinase 1. Oncotarget. 2016;7(34):54515–25. doi: 10.18632/oncotarget.10248 27363027 PMC5342359

[10] Ferrer M, Gosline SJC, Stathis M, Zhang X, Guo X, Guha R, et al. Pharmacological and genomic profiling of neurofibromatosis type 1 plexiform neurofibroma-derived schwann cells. Sci Data. 2018;5:180106. doi: 10.1038/sdata.2018.106 29893754 PMC5996849

[11] Borisy AA, Elliott PJ, Hurst NW, Lee MS, Lehar J, Price ER, et al. Systematic discovery of multicomponent therapeutics. Proc Natl Acad Sci U S A. 2003;100(13):7977–82. doi: 10.1073/pnas.1337088100 12799470 PMC164698

[12] Markham A. Brigatinib: First Global Approval. Drugs. 2017;77(10):1131–5. doi: 10.1007/s40265-017-0776-3 28597393

[13] Zhang S, Anjum R, Squillace R, Nadworny S, Zhou T, Keats J, et al. The Potent ALK Inhibitor Brigatinib (AP26113) Overcomes Mechanisms of Resistance to First- and Second-Generation ALK Inhibitors in Preclinical Models. Clin Cancer Res. 2016;22(22):5527–38. doi: 10.1158/1078-0432.CCR-16-0569 27780853

[14] Huang WS, Liu S, Zou D, Thomas M, Wang Y, Zhou T, et al. Discovery of Brigatinib (AP26113), a Phosphine Oxide-Containing, Potent, Orally Active Inhibitor of Anaplastic Lymphoma Kinase. J Med Chem. 2016;59(10):4948–64. doi: 10.1021/acs.jmedchem.6b00306 27144831

[15] U.S. Food and Drug Administration, Center for Drug Evaluation and Research. Alunbrig (brigatinib). NDA 208772 Multidisciplinary Review and Evaluation. April 28, 2017. Retrieved April 15, 2021 from https://www.accessdata.fda.gov/drugsatfda_docs/nda/2017/208772Orig1s000MultidisciplineR.pdf.

[16] Daub H, Olsen JV, Bairlein M, Gnad F, Oppermann FS, Korner R, et al. Kinase-selective enrichment enables quantitative phosphoproteomics of the kinome across the cell cycle. Mol Cell. 2008;31(3):438–48. doi: 10.1016/j.molcel.2008.07.007 18691976

[17] Duncan JS, Whittle MC, Nakamura K, Abell AN, Midland AA, Zawistowski JS, et al. Dynamic reprogramming of the kinome in response to targeted MEK inhibition in triple-negative breast cancer. Cell. 2012;149(2):307–21. doi: 10.1016/j.cell.2012.02.053 22500798 PMC3328787

[18] Stuhlmiller TJ, Miller SM, Zawistowski JS, Nakamura K, Beltran AS, Duncan JS, et al. Inhibition of Lapatinib-Induced Kinome Reprogramming in ERBB2-Positive Breast Cancer by Targeting BET Family Bromodomains. Cell Rep. 2015;11(3):390–404. doi: 10.1016/j.celrep.2015.03.037 25865888 PMC4408261

[19] Klaeger S, Heinzlmeir S, Wilhelm M, Polzer H, Vick B, Koenig PA, et al. The target landscape of clinical kinase drugs. Science. 2017;358(6367). doi: 10.1126/science.aan4368 29191878 PMC6542668

[20] Zhou Y, Liu Z, Rothschild KJ, Lim MJ. Proteome-wide drug screening using mass spectrometric imaging of bead-arrays. Sci Rep. 2016;6:26125. doi: 10.1038/srep26125 27194112 PMC4872124

[21] Angus SP, Oblinger JL, Stuhlmiller TJ, DeSouza PA, Beauchamp RL, Witt L, et al. EPH receptor signaling as a novel therapeutic target in NF2-deficient meningioma. Neuro Oncol. 2018;20(9):1185–96. doi: 10.1093/neuonc/noy046 29982664 PMC6071664

[22] Yap TA, Yan L, Patnaik A, Fearen I, Olmos D, Papadopoulos K, et al. First-in-man clinical trial of the oral pan-AKT inhibitor MK-2206 in patients with advanced solid tumors. J Clin Oncol. 2011;29(35):4688–95. doi: 10.1200/JCO.2011.35.5263 22025163

[23] Beauchamp RL, James MF, DeSouza PA, Wagh V, Zhao WN, Jordan JT, et al. A high-throughput kinome screen reveals serum/glucocorticoid-regulated kinase 1 as a therapeutic target for NF2-deficient meningiomas. Oncotarget. 2015;6(19):16981–97. doi: 10.18632/oncotarget.4858 26219339 PMC4627286

[24] Dombi E., Baldwin A., Marcus L. J., Fisher M. J., Weiss B., Kim A., et al. Activity of Selumetinib in Neurofibromatosis Type 1-Related Plexiform Neurofibromas. NEJMoa, 2016;375(26):2550–2560. doi: 10.1056/NEJMoa1605943 28029918 PMC5508592

[25] Gross A. M., Wolters P. L., Dombi E., Baldwin A., Whitcomb P., Fisher M. J., et al. Selumetinib in Children with Inoperable Plexiform Neurofibromas. NEJMoa, 2020;382(15):1430–1442. doi: 10.1056/NEJMoa1912735 32187457 PMC7305659

[26] Fisher M. J., Shih C. S., Rhodes S. D., Armstrong A. E., Wolters P. L., Dombi E., et al. Cabozantinib for neurofibromatosis type 1-related plexiform neurofibromas: a phase 2 trial. Nat Med., 2021;27(1):165–173. doi: 10.1038/s41591-020-01193-6 33442015 PMC8275010

[27] Petrilli AM, Garcia J, Bott M, Klingeman Plati S, Dinh CT, Bracho OR, et al. Ponatinib promotes a G1 cell-cycle arrest of merlin/NF2-deficient human schwann cells. Oncotarget. 2017;8(19):31666–81. doi: 10.18632/oncotarget.15912 28427224 PMC5458238

[28] Burns SS, Akhmametyeva EM, Oblinger JL, Bush ML, Huang J, Senner V, et al. Histone deacetylase inhibitor AR-42 differentially affects cell-cycle transit in meningeal and meningioma cells, potently inhibiting NF2-deficient meningioma growth. Cancer Res. 2013;73(2):792–803. doi: 10.1158/0008-5472.CAN-12-1888 23151902 PMC3549000

[29] Puttmann S, Senner V, Braune S, Hillmann B, Exeler R, Rickert CH, et al. Establishment of a benign meningioma cell line by hTERT-mediated immortalization. Lab Invest. 2005;85(9):1163–71. doi: 10.1038/labinvest.3700307 15965488

[30] Di Veroli GY, Fornari C, Wang D, Mollard S, Bramhall JL, Richards FM, et al. Combenefit: an interactive platform for the analysis and visualization of drug combinations. Bioinformatics. 2016;32(18):2866–8. doi: 10.1093/bioinformatics/btw230 27153664 PMC5018366

[31] Brighton HE, Angus SP, Bo T, Roques J, Tagliatela AC, Darr DB, et al. New Mechanisms of Resistance to MEK Inhibitors in Melanoma Revealed by Intravital Imaging. Cancer Res. 2018;78(2):542–57. doi: 10.1158/0008-5472.CAN-17-1653 29180473 PMC6132242

[32] Cox J, Hein MY, Luber CA, Paron I, Nagaraj N, Mann M. Accurate proteome-wide label-free quantification by delayed normalization and maximal peptide ratio extraction, termed MaxLFQ. Mol Cell Proteomics. 2014;13(9):2513–26. doi: 10.1074/mcp.M113.031591 24942700 PMC4159666

[33] Gehlhausen JR, Park SJ, Hickox AE, Shew M, Staser K, Rhodes SD, et al. A murine model of neurofibromatosis type 2 that accurately phenocopies human schwannoma formation. Hum Mol Genet. 2015;24(1):1–8. doi: 10.1093/hmg/ddu414 25113746 PMC4262489

