# Supplementary figures and images for: Brigatinib causes tumor shrinkage in both NF2-deficient meningioma and schwannoma through inhibition of multiple tyrosine kinases but not ALK
Source: PLoS One. 2021 Jul 15;16(7):e0252048. doi: 10.1371/journal.pone.0252048 (PMC8282008; doi:10.1371/journal.pone.0252048)

Fig. S1

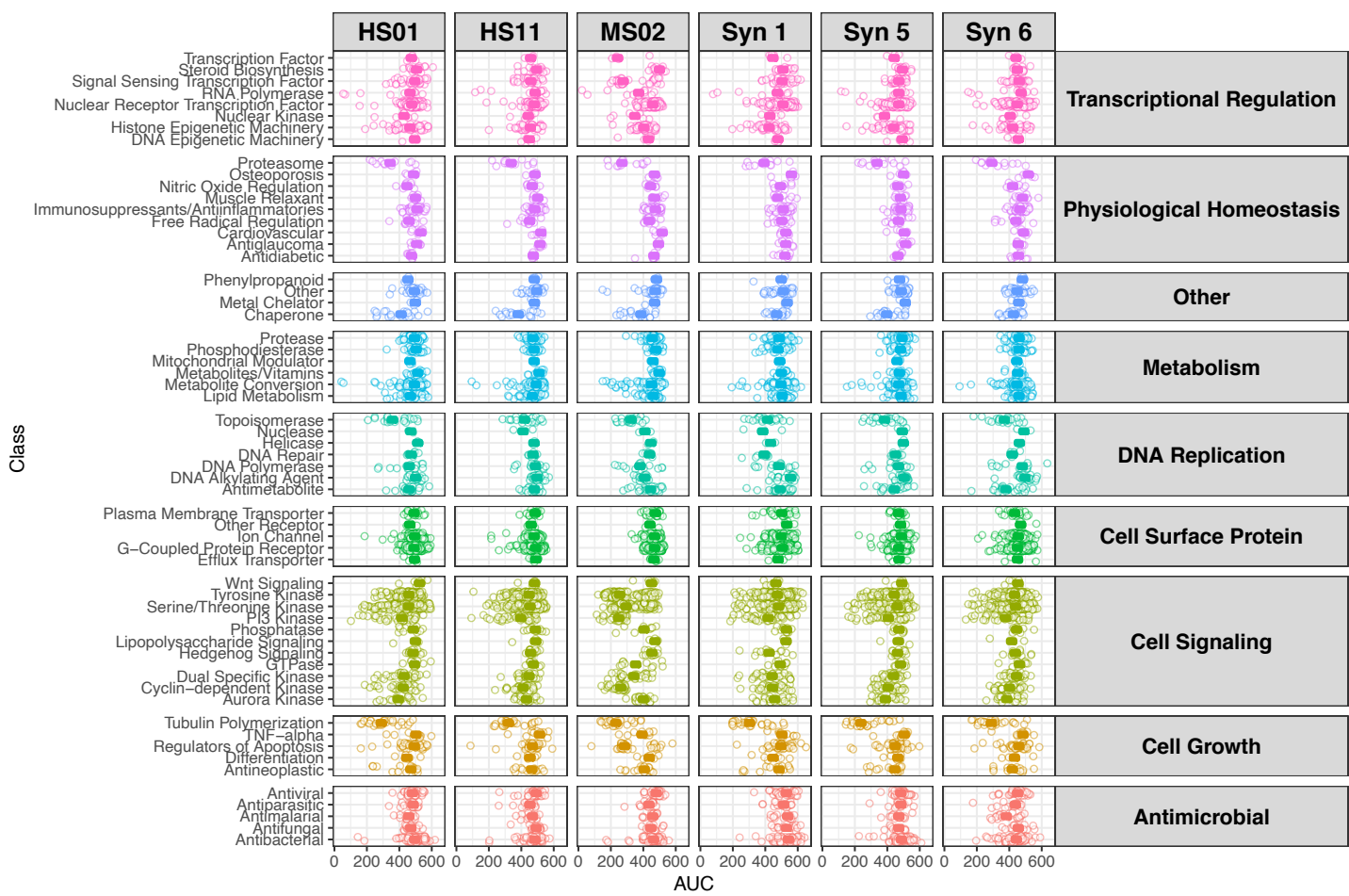

Supplement: S1 Fig — Solid dots represent the median response for all the compounds, in each mechanistic class, and for each cell line. (PDF) [file pone.0252048.s001.pdf]

Fig. S6

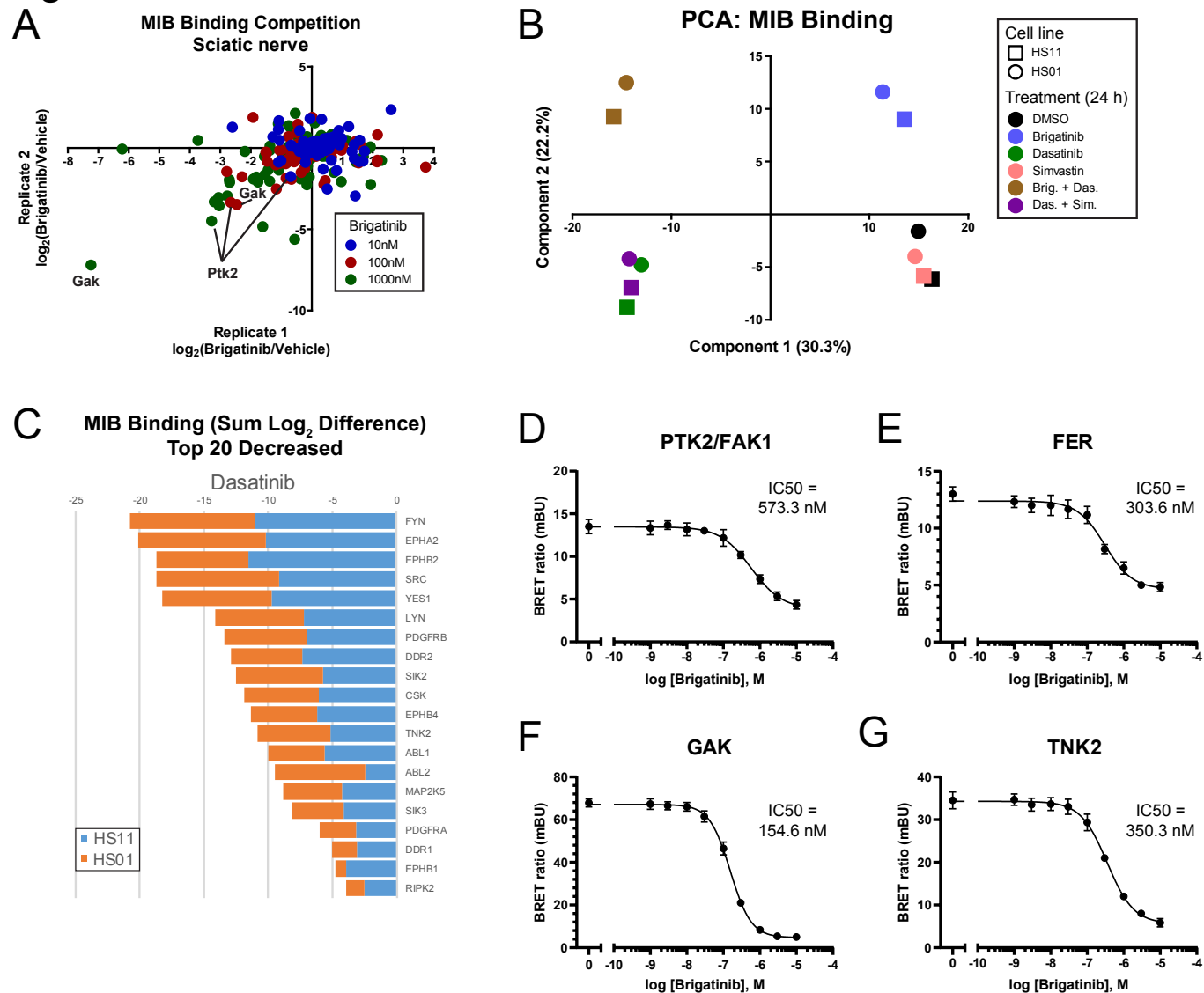

Supplement: S6 Fig — (A) Control sciatic nerve tissues from Postn-Cre; Nf2flox/flox mice were used to prepare lysates. Equal amount of protein lysates (1mg) was incubated with vehicle (0.001% ethanol) or brigatinib (10nM, blue; 100nM, red; 1000nM, green) for 2h in biological duplicate. The log2 difference in MIB binding (LFQ intensity) was calculated and plotted relative to vehicle for each replicate. (B) HS11 and HS01 cells were treated with vehicle or brigatinib (1μM) for 24h prior to MIB/MS kinome profiling. MIB binding (log2LFQ intensities) was determined and PCA performed in Perseus. (C) HS11 and HS01 cells were treated with vehicle or dasatinib (0.6μM) for 24h prior to MIB/MS kinome profiling. MIB binding (log2LFQ intensities) was determined and the top 20 kinases with decreased MIB binding compared to vehicle are shown as a stacked bar plot for the two cell lines. (D-G). NanoBRET target engagement intracellular kinase assays were performed using the indicated kinases (PTK2/FAK1, FER, GAK, and TNK2) expressed as NanoLuc fusion constructs in HEK293T cells. Cells were treated with the indicated concentration of vehicle or brigatinib for 2 h prior to reading the plates. The 450/50 nm and long-pass 610 nm emission values were used to determine the NanoBRET™ ratios. Values were plotted in Prism and the best-fit IC50 values are indicated. Representative data is shown from experiments performed at least twice. (PDF) [file pone.0252048.s006.pdf]

Fig. S7

ALK was not detected in normal meningeal and *NF2*-deficient or expressing meningioma cells

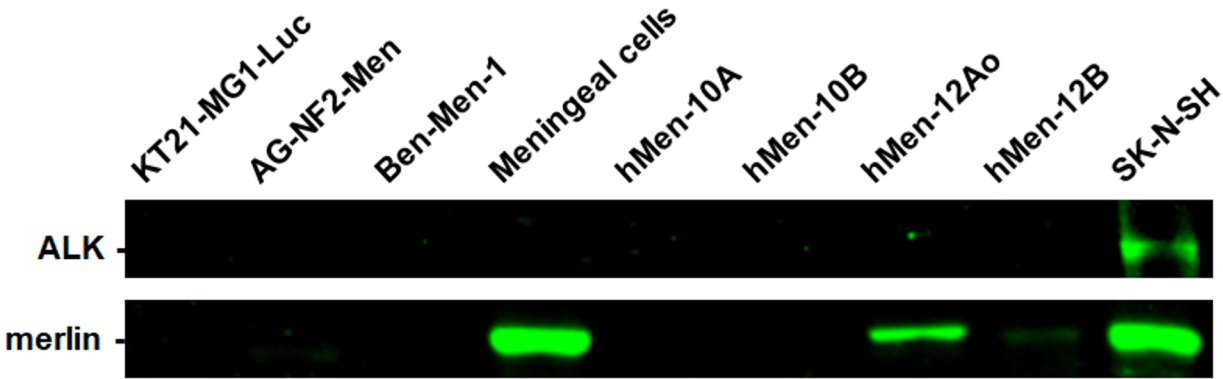

Supplement: S7 Fig — Equal amounts of protein lysates from three human meningioma cell lines (KT21-MG1-Luc, NF2-Men-1, and Ben-Men-1), normal meningeal cells, and four primary meningioma cell cultures (hMen-10A, hMen-10B, hMen-12Ao, hMen-12B) were used in Western blot analysis to probe ALK and merlin expression. SK-N-SH neuroblastoma cells, which express ALK, were used as a positive control. (PDF) [file pone.0252048.s007.pdf]

Fig. S8

A

10% FBS

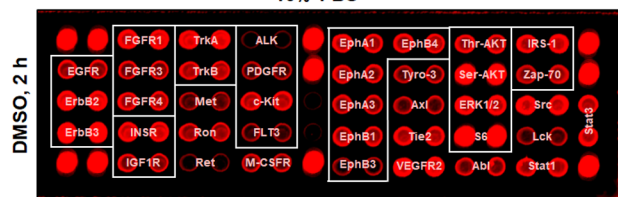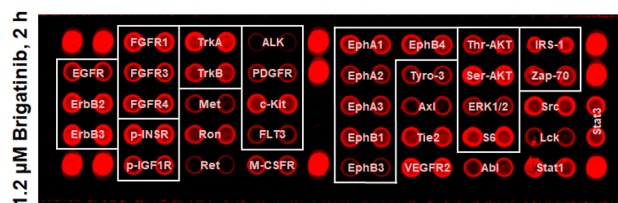

|       |       |       |       |   |       |       |       |       |       |
|-------|-------|-------|-------|---|-------|-------|-------|-------|-------|
| +     | 0.711 | 0.891 | 1.164 | + | 0.732 | 0.730 | 0.790 | 0.530 | +     |
| 0.696 | 0.818 | 0.771 | 0.749 | + | 0.501 | 0.748 | 0.327 | 1.167 | +     |
| 0.696 | 0.763 | 0.744 | 0.715 | - | 0.687 | 0.701 | 0.238 | 0.506 | 0.717 |
| 0.689 | 0.712 | 0.827 | 0.829 | - | 0.686 | 0.704 | 0.243 | 1.094 |       |
| +     | 0.771 | 1.073 | 0.787 | + | 0.616 | 0.696 | 0.662 | 0.765 | +     |

B

10% FBS

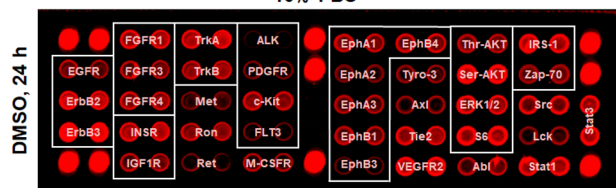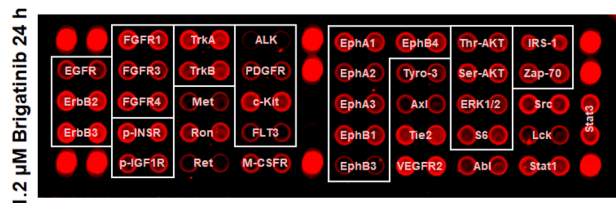

|       |       |       |       |   |       |       |       |       |       |
|-------|-------|-------|-------|---|-------|-------|-------|-------|-------|
| +     | 1.004 | 1.021 | 1.163 | + | 1.082 | 1.011 | 0.934 | 0.289 | +     |
| 1.015 | 0.696 | 0.978 | 1.138 | + | 1.092 | 1.039 | 0.384 | 1.075 | +     |
| 1.019 | 1.007 | 0.903 | 1.117 | - | 0.993 | 1.003 | 0.503 | 1.050 | 0.822 |
| 0.892 | 0.930 | 0.983 | 1.189 | - | 1.023 | 0.971 | 0.428 | 1.254 |       |
| +     | 0.977 | 0.868 | 1.023 | + | 1.096 | 0.957 | 0.599 | 0.567 | +     |

C

Growth arrest + 20% FBS

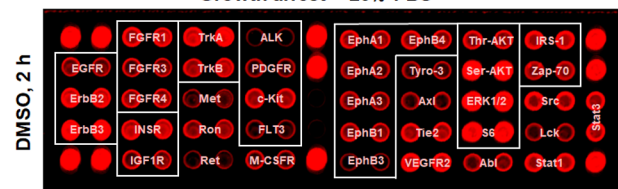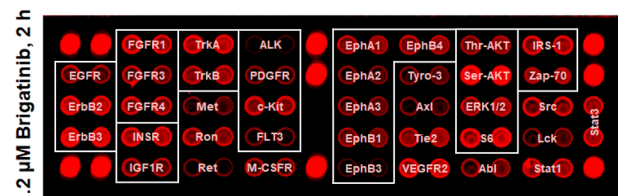

|       |       |       |       |   |       |       |       |       |       |
|-------|-------|-------|-------|---|-------|-------|-------|-------|-------|
| +     | 0.764 | 0.665 | 0.618 | + | 0.723 | 0.734 | 0.641 | 0.755 | +     |
| 0.790 | 1.187 | 0.770 | 0.742 | + | 0.598 | 0.750 | 0.545 | 0.651 | +     |
| 0.783 | 0.843 | 0.868 | 0.811 | - | 0.727 | 0.692 | 0.282 | 0.676 | 0.776 |
| 0.733 | 0.797 | 0.903 | 0.766 | - | 0.705 | 0.728 | 0.397 | 0.769 |       |
| +     | 0.803 | 0.909 | 0.838 | + | 0.642 | 0.779 | 0.707 | 0.744 | +     |

D

Growth arrest + 20% FBS

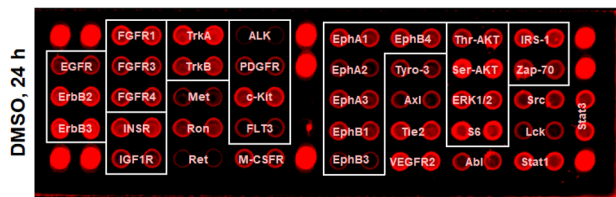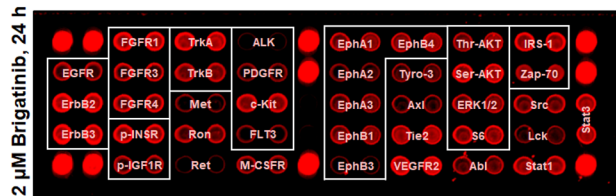

|       |       |       |       |   |       |       |       |       |       |
|-------|-------|-------|-------|---|-------|-------|-------|-------|-------|
| +     | 1.108 | 0.869 | 0.770 | + | 0.970 | 1.000 | 0.569 | 1.009 | +     |
| 1.068 | 1.100 | 0.948 | 0.994 | + | 1.029 | 1.013 | 0.532 | 0.308 | +     |
| 1.130 | 1.115 | 0.934 | 1.036 | - | 0.962 | 0.974 | 0.673 | 0.765 | 0.956 |
| 0.879 | 1.070 | 1.024 | 0.947 | - | 0.994 | 1.012 | 0.534 | 0.927 |       |
| +     | 0.969 | 0.818 | 0.899 | + | 1.046 | 0.972 | 0.677 | 0.855 | +     |

Supplement: S8 Fig — PathScan® RTK signaling antibody array analysis was conducted according to Supplementary Methods in S1 File using cells grown in 10% FBS and treated with 1x IC50 of brigatinib for 2h (A) and 24 h (B) or in growth-arrested cells stimulated with 20% FBS in the presence of 1x IC50 of brigatinib for 2h (C) and 24 h (D). The numeric table below each pair of arrays displays the fold-change in fluorescence detected for each phospho-protein expressed in cells treated with brigatinib relative to DMSO control after subtraction of the fluorescence in the background control spots (indicated as “-”). Positive control spots are denoted as “+”. (PDF) [file pone.0252048.s008.pdf]

Fig. S9

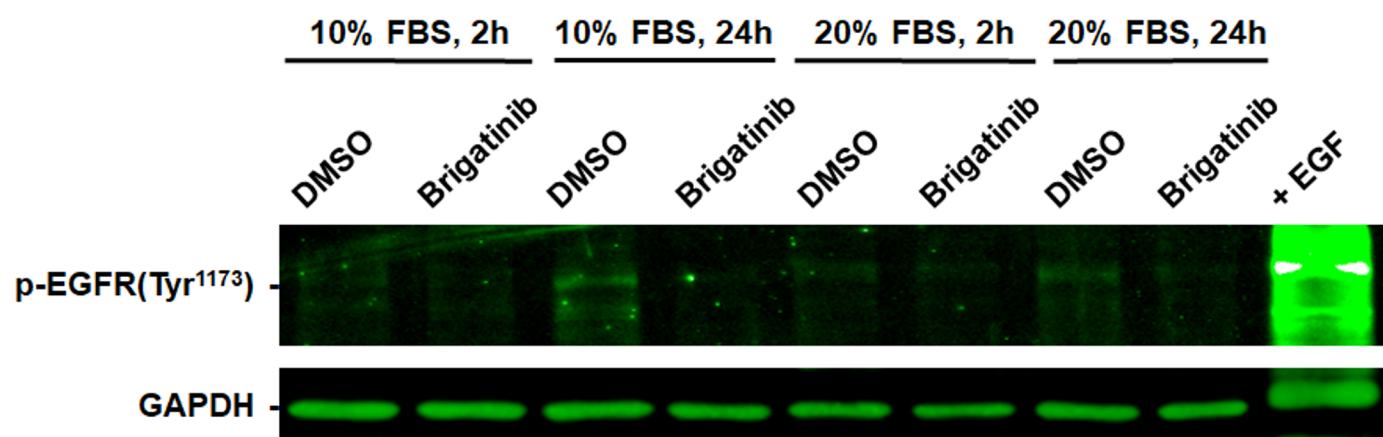

Supplement: S9 Fig — Western blot analysis revealed that while phosphorylated EGFR was robustly induced in EGF-stimulated Ben-Men-1 cells, only very low levels of p-EGFR were detected in actively-growing cells or growth-arrested cells stimulated with 20% serum for 24h. Brigatinib treatment diminished p-EGFR expression. (PDF) [file pone.0252048.s009.pdf]

Fig. S10

**A**

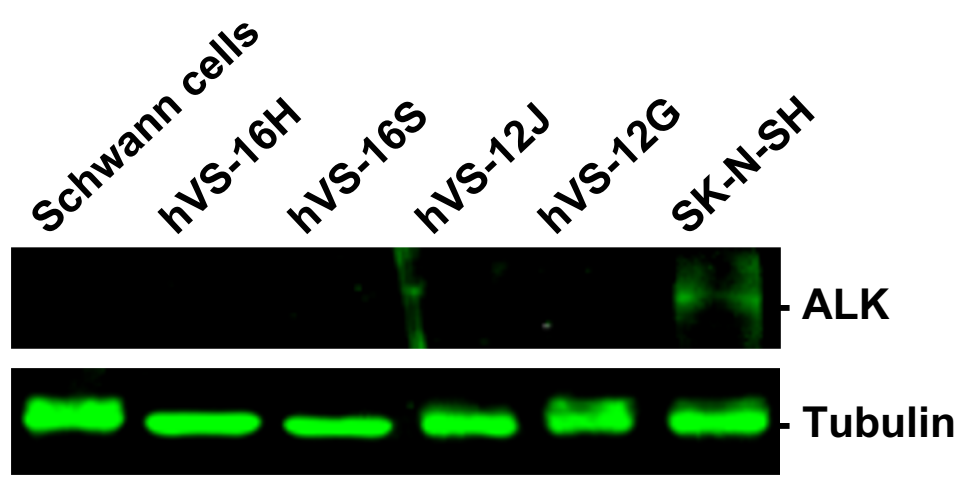

**B**

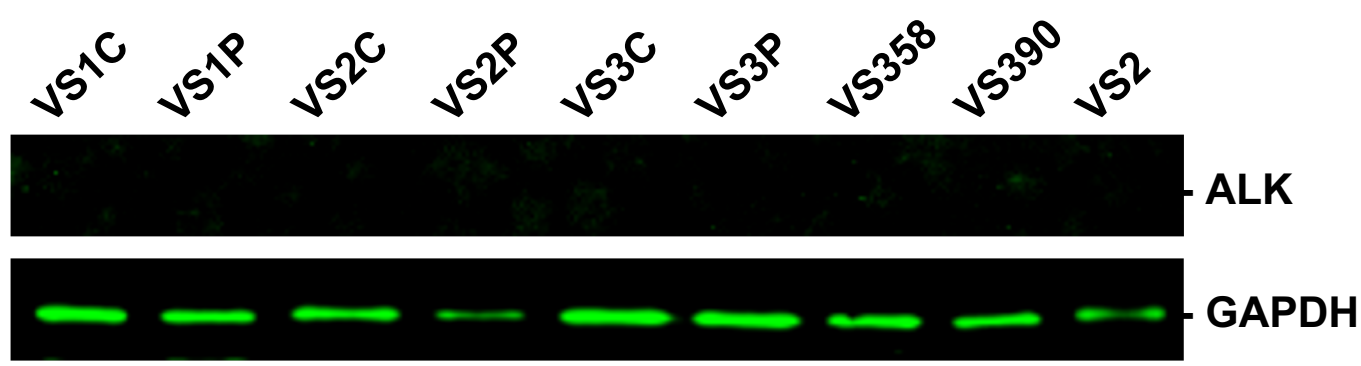

Supplement: S10 Fig — (A) Western blotting was performed to detect ALK expression in human Schwann cells, four primary vestibular schwannoma cultures (hVS-16H, hVS-16S, hVS-12J, hVS-12Ga), and SK-N-SH neuroblastoma cells. Tubulin was used as a loading control. (B) Western blotting for ALK was also performed using extracts from nine vestibular schwannoma tumors. GAPDH was used as a loading control. (PDF) [file pone.0252048.s010.pdf]

Fig. S13

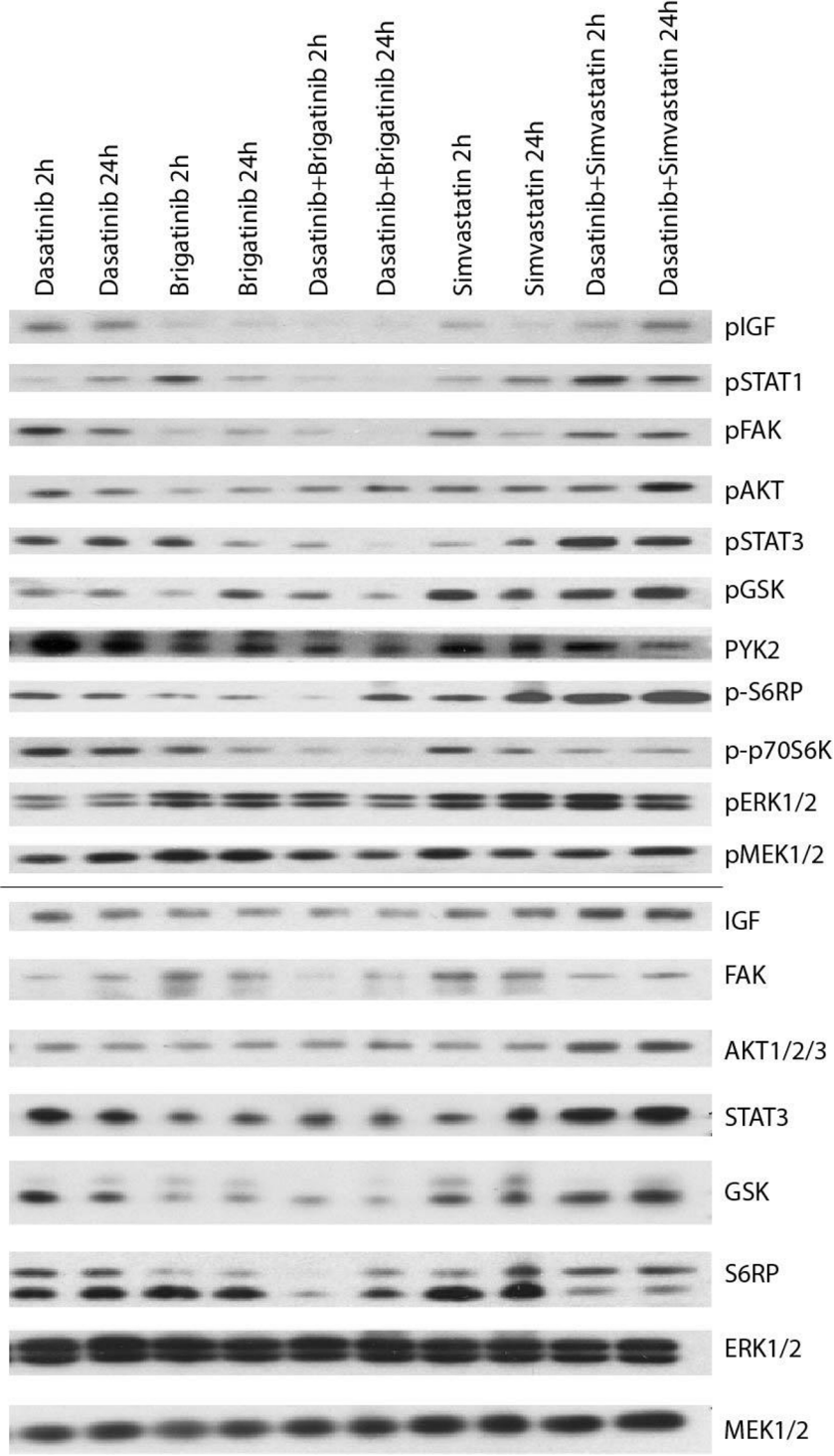

Supplement: S13 Fig — DRGs from Postn-Cre;Nf2flox/flox mice treated with the indicated drug or drug combination were used in Western blotting as described in Supplementary Methods in S1 File. Mice were treated for 12 weeks with the following drug dosages: 20mg/kg dasatinib, 50mg/kg brigatinib, 20mg/kg dasatinib + 15mg/kg brigatinib, 100mg/kg simvastatin, and 20mg/kg dasatinib + 100mg/kg simvastatin. DRG tissues were harvested 2 or 24 hours after the last dose of drug. (PDF) [file pone.0252048.s013.pdf]
