# Supplementary material for: Brigatinib causes tumor shrinkage in both NF2-deficient meningioma and schwannoma through inhibition of multiple tyrosine kinases but not ALK
Source: PLoS One. 2021 Jul 15;16(7):e0252048. doi: 10.1371/journal.pone.0252048 (PMC8282008; doi:10.1371/journal.pone.0252048)
Supplement: S2 Fig — Drug treatment of the NF2-expressing Syn1(+) and NF2-null Syn5(-) cells (A), Ben-Men-1 cells (B), and two primary meningioma cultures MN612 and MN621 (C) was performed using the same drug doses as for Fig 2C and 2D. Single drug DRCs and combination drug heatmaps were generated using Combenefit software. Note that heatmap data for Ben-Men-1 (B) is the same as shown in Fig 2D. (PDF) [file pone.0252048.s002.pdf]

Fig. S2

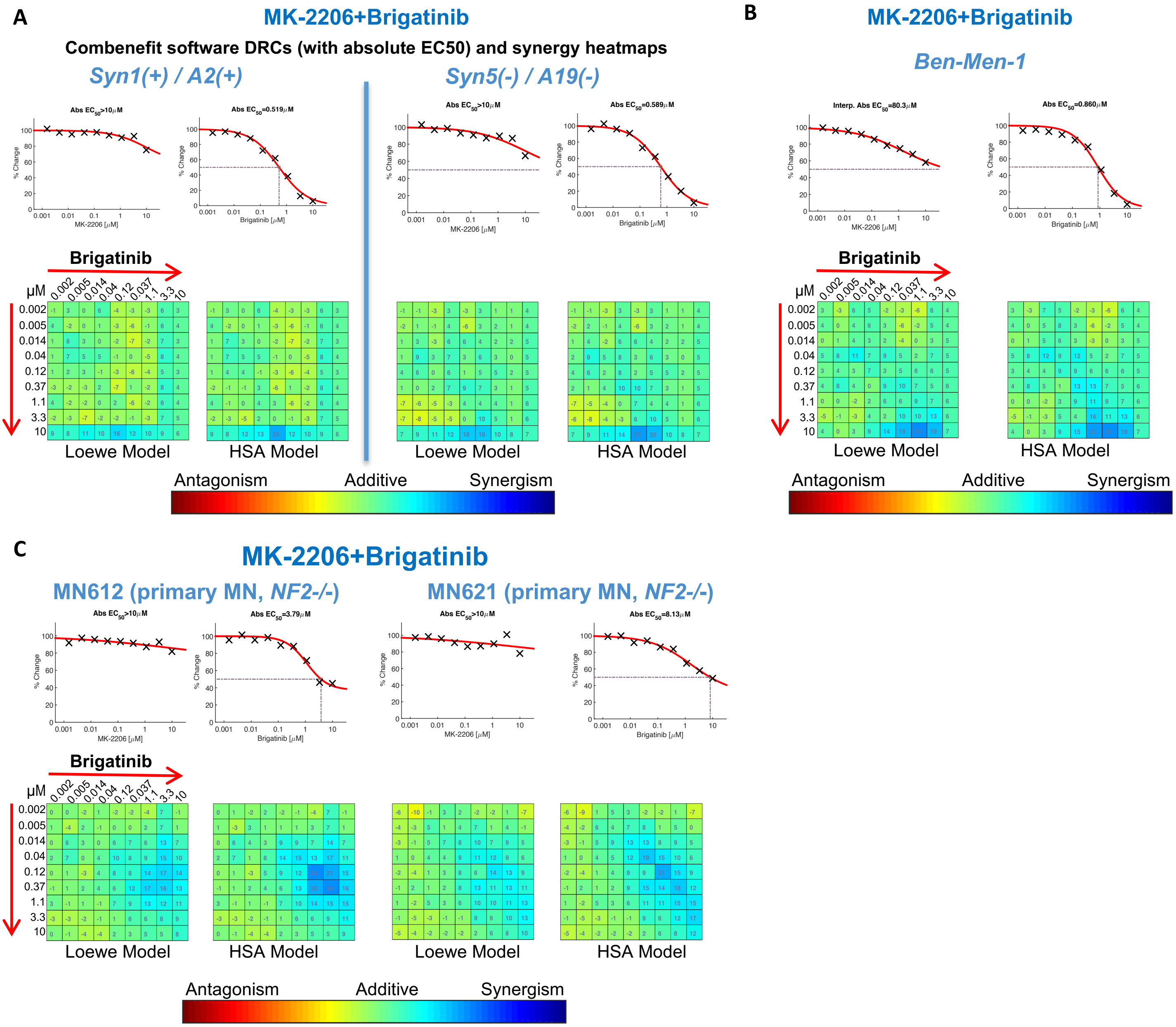
A
MK-2206+Brigatinib
Combenefit software DRCs (with absolute EC50) and synergy heatmaps
Syn1(+) / A2(+)
Syn5(-) / A19(-)
Abs EC50>10μM
Abs EC50=0.519μM
Abs EC50>10μM
Abs EC50=0.589μM
MK-2206 [μM]
Brigatinib [μM]
% Change
Brigatinib
μM
Loewe Model
HSA Model
Loewe Model
HSA Model
Antagonism
Additive
Synergism
B
MK-2206+Brigatinib
Ben-Men-1
Interp. Abs EC50=80.3μM
Abs EC50=0.860μM
Brigatinib
Loewe Model
HSA Model
Antagonism
Additive
Synergism
C
MK-2206+Brigatinib
MN612 (primary MN, NF2-/-)
MN621 (primary MN, NF2-/-)
Abs EC50>10μM
Abs EC50=3.79μM
Abs EC50>10μM
Abs EC50=8.13μM
Brigatinib
Loewe Model
HSA Model
Loewe Model
HSA Model
Antagonism
Additive
Synergism
