# Supplementary material for: Brigatinib causes tumor shrinkage in both NF2-deficient meningioma and schwannoma through inhibition of multiple tyrosine kinases but not ALK
Source: PLoS One. 2021 Jul 15;16(7):e0252048. doi: 10.1371/journal.pone.0252048 (PMC8282008; doi:10.1371/journal.pone.0252048)

Fig. S3

A ALK inhibitors: Single Agent

Cell Line: HS01

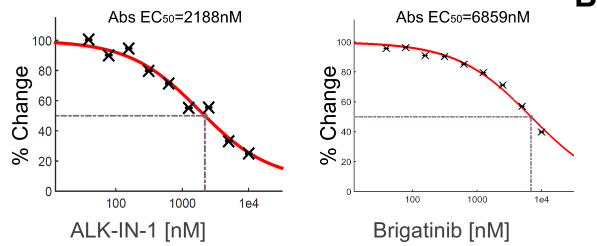

Cell Line: MS02

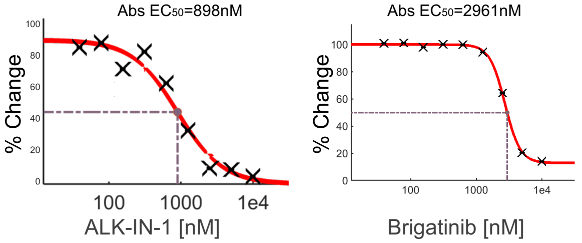

B

HS01
Brigatinib-Dasatinib
ALK-BCR/ABL

Combination dose-response data in matrix format

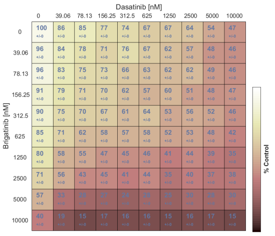

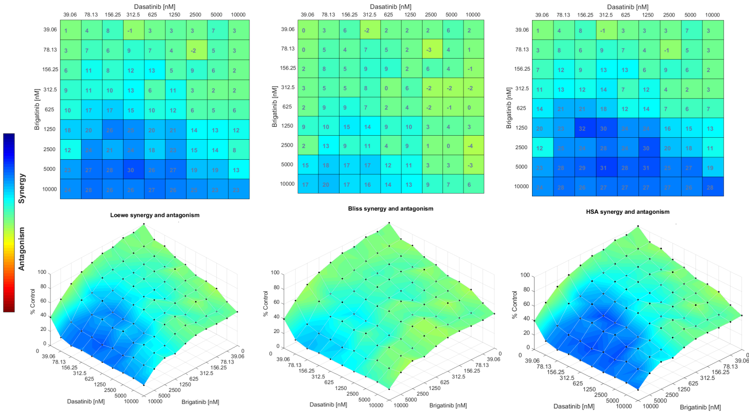

C

MS02
Brigatinib-Dasatinib
Alk-Bcr/Abl

Combination dose-response data in matrix format

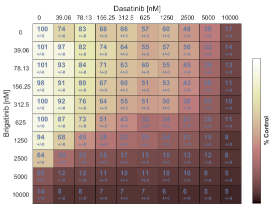

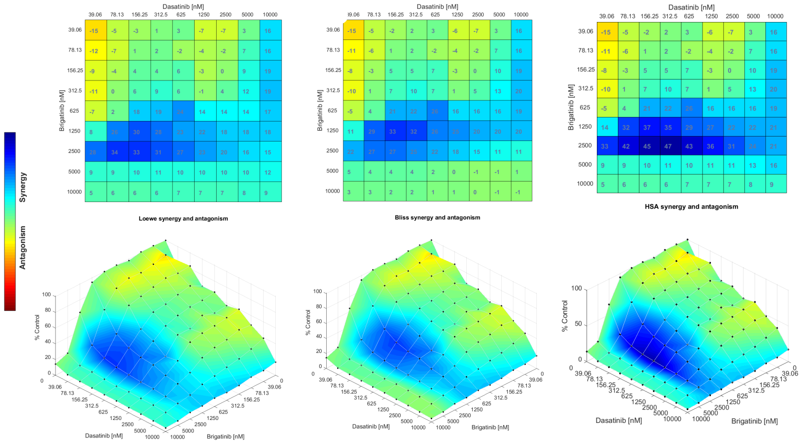

Supplement: S3 Fig — (A) Nf2-/- mouse MS02 schwannoma and NF2-deficient human HS01 Schwann cells were plated and tested in dose-response 10x10 matrix format as described in Methods. Shown are single-agent dose response curves for ALK-IN-1 and brigatinib. (B) Brigatinib/dasatinib combination matrix analyses were conducted with HS01 cells. Shown are the brown-scale viability and synergy matrix plots with modeled surface synergy distributions in Loewe, Bliss, and HSA models. (C) Brigatinib/dasatinib combination matrix were analyzed with MS02 cells as described in S3B Fig. (PDF) [file pone.0252048.s003.pdf]
