# Supplementary material for: Brigatinib causes tumor shrinkage in both NF2-deficient meningioma and schwannoma through inhibition of multiple tyrosine kinases but not ALK
Source: PLoS One. 2021 Jul 15;16(7):e0252048. doi: 10.1371/journal.pone.0252048 (PMC8282008; doi:10.1371/journal.pone.0252048)
Supplement: S4 Fig — 10x10 matrix dose-response analysis was performed for NF2-deficient HS01 (A) and NF2-expressing HS11 (B) human Schwann cells and Nf2-/- mouse schwannoma MS02 cells (C) treated with the brigatinib/simvastatin combination. Shown are the viability (brown scale) and synergy matrix (blue to red scale) plots with modeled surface synergy distributions in Loewe, Bliss, and HSA models. (PDF) [file pone.0252048.s004.pdf]

Fig. S4

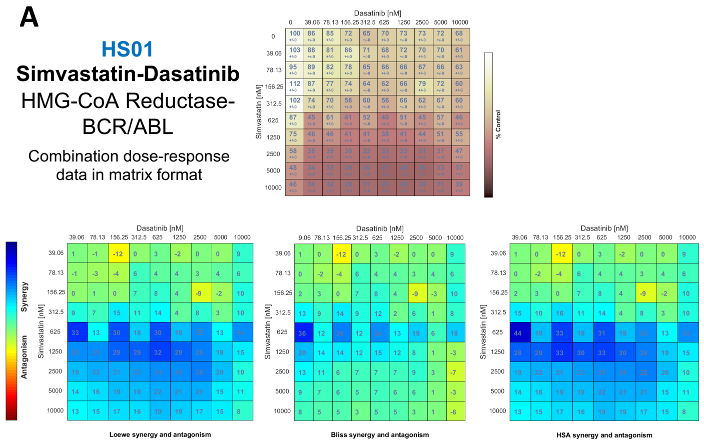
A
HS01
Simvastatin-Dasatinib
HMG-CoA Reductase-BCR/ABL
Combination dose-response data in matrix format
Dasatinib [nM]
Simvastatin [nM]
% Control
Synergy
Antagonism
Loewe synergy and antagonism
Bliss synergy and antagonism
HSA synergy and antagonism

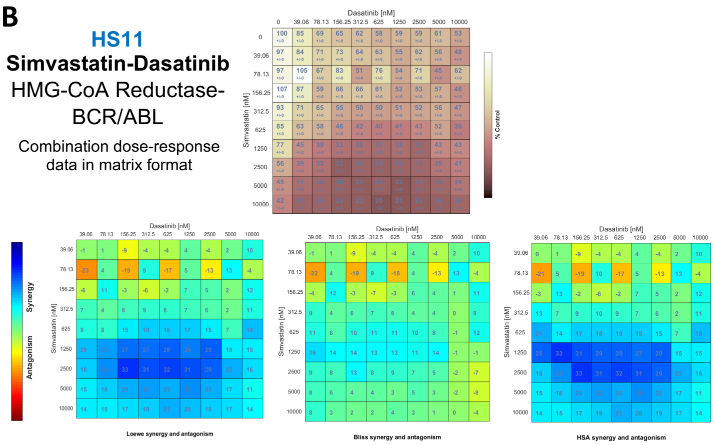
B
HS11
Simvastatin-Dasatinib
HMG-CoA Reductase-BCR/ABL
Combination dose-response data in matrix format
Dasatinib [nM]
Simvastatin [nM]
% Control
Synergy
Antagonism
Loewe synergy and antagonism
Bliss synergy and antagonism
HSA synergy and antagonism

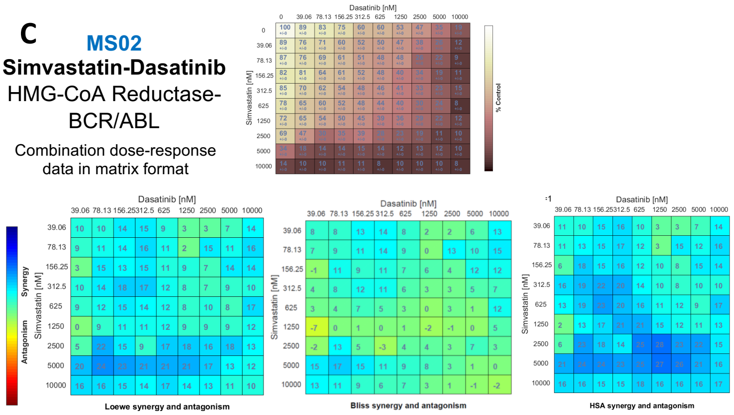
C
MS02
Simvastatin-Dasatinib
HMG-CoA Reductase-BCR/ABL
Combination dose-response data in matrix format
Dasatinib [nM]
Simvastatin [nM]
% Control
Synergy
Antagonism
Loewe synergy and antagonism
Bliss synergy and antagonism
HSA synergy and antagonism
