# Supplementary material for: Brigatinib causes tumor shrinkage in both NF2-deficient meningioma and schwannoma through inhibition of multiple tyrosine kinases but not ALK
Source: PLoS One. 2021 Jul 15;16(7):e0252048. doi: 10.1371/journal.pone.0252048 (PMC8282008; doi:10.1371/journal.pone.0252048)
Supplement: S5 Fig — (A) Significant (Bonferroni-adjusted p < 0.05) changes in gene expression in Syn5 and Syn1 arachnoidal cells, which show extensive overlap with an overall greater response in the Syn5 merlin-null cells, are caused by brigatinib or brigatinib/MK-2206 treatment, but not by MK-2206 alone. (B) Genes whose expression is altered both by lack of merlin in Syn5 and by treatment of Syn5 with either brigatinib or brigatinib/MK-2206 treatment overwhelmingly show the expression change in opposite directions. (C) Significant changes in gene expression in HS01 and HS11 Schwann cells are limited in single drug treatments and most pronounced with the brigatinib/dasatinib combination. (D) The small number of genes differentially expressed in HS01 as a result of merlin expression do not overlap extensively with those altered in HS01 by the brigatinib/dasatinib treatment, and those overlaps that do occur are concordant in direction, contrasting with the arachnoidal cells. (PDF) [file pone.0252048.s005.pdf]

Fig. S5

A

		Syn5	Syn1	Shared	Discrepant
**Brigatinib**	Up	1281	242	180	0
	Down	1324	318	276	0
**MK-2206**	Up	0	0	0	0
	Down	0	0	0	0
**Brigatinib + MK-2206**	Up	1091	337	226	0
	Down	1080	493	399	0

B

	DEG in Syn5 + Brigatinib vs. Syn5 Untreated		
**DEG in Untreated Syn5 vs. Syn1**		Up (1281)	Down (1324)
	Up (1034)	5	327
	Down (1424)	653	3

	DEG in Syn5 + Brigatinib + MK-2206 vs. Syn5 Untreated		
**DEG in Untreated Syn5 vs. Syn1**		Up (1091)	Down (1080)
	Up (1034)	3	262
	Down (1424)	567	0

C

		HS01	HS11	Shared	Discrepant
**Brigatinib**	Up	0	0	0	0
	Down	0	1	0	0
**Dasatinib**	Up	18	19	8	0
	Down	7	21	2	0
**Brigatinib + Dasatinib**	Up	731	451	324	0
	Down	666	581	355	0
**Simvastatin**	Up	35	16	12	0
	Down	7	6	4	0
**Dasatinib + Simvastatin**	Up	171	102	72	0
	Down	174	283	108	0

D

	DEG in HS01 + Brigatinib + Dasatinib vs. HS01 Untreated		
**DEG in Untreated HS01 vs. HS11**		Up (731)	Down (666)
	Up (40)	11	0
	Down (15)	0	1
