# Supplementary material for: Brigatinib causes tumor shrinkage in both NF2-deficient meningioma and schwannoma through inhibition of multiple tyrosine kinases but not ALK
Source: PLoS One. 2021 Jul 15;16(7):e0252048. doi: 10.1371/journal.pone.0252048 (PMC8282008; doi:10.1371/journal.pone.0252048)
Supplement: S11 Fig — Mice were orally fed 50 mg/kg of brigatinib or 120 mg/kg of MK-2206. PK analysis was performed according to Supplementary Methods in S1 File. (PDF) [file pone.0252048.s011.pdf]

Fig. S11

Mouse pharmacokinetic parameters following a single oral dose of brigatinib (50 mg/kg) or MK-2206 (120 mg/kg)

Drug	PK Parameters					
	$T_{1/2}$ (h)	T_{max} (h)	C_{max} (ng/mL)	$AUC_{0-\infty}$ (h*ng/mL)	V/F (L/kg)	CL/F (L/h/kg)
**Plasma**						
Brigatinib	7.2	4	1311	18662	27.8	2.7
MK2206	5.6	1	2208	21286	45.3	5.6
**Brain (Gross)**						
	$T_{1/2}$ (h)	T_{max} (h)	C_{max} (ng/g)	$AUC_{0-\infty}$ (h*ng/g)	V/F (kg/kg)	CL/F (kg/h/kg)
Brigatinib	6.2	4	170	2319	193.3	22.6
MK2206	6.0	4	406	5462	191.5	22.0
