# Supplementary material for: Brigatinib causes tumor shrinkage in both NF2-deficient meningioma and schwannoma through inhibition of multiple tyrosine kinases but not ALK
Source: PLoS One. 2021 Jul 15;16(7):e0252048. doi: 10.1371/journal.pone.0252048 (PMC8282008; doi:10.1371/journal.pone.0252048)
Supplement: S12 Fig — Detailed drug treatment and tumor measurement were as described in Fig 5B and 5C and Supplementary Methods in S1 File. (PDF) [file pone.0252048.s012.pdf]

Fig. S12

Relative tumor sizes in meningioma-bearing mice prior to or after treatment with MK-2206 and brigatinib, either alone or in combination

Week	Vehicle control	SD	MK-2206	SD	Brigatinib	SD	MK-2206 + Brigatinib	SD
0	1	0	1	0	1	0	1	0
2	2.00	0.77	1.31	0.58	1.02	0.44	0.35	0.20
4	2.88	1.33	1.68	0.54	0.79	0.44	0.28	0.18
6	4.29	2.13	2.24	1.01	1.02	0.37	0.31	0.18
8	4.85	2.62	2.52	1.05	0.54	0.14	0.34	0.21
10	5.39	2.63	3.35	1.31	0.75	0.23	0.39	0.20
12	6.69	2.85	4.43	1.86	0.62	0.20	0.37	0.18

The relative tumor size shown following drug treatment was normalized to the tumor size prior to treatment (week 0), which is designated as 1 (100%).
