# Supplementary material for: Brigatinib causes tumor shrinkage in both NF2-deficient meningioma and schwannoma through inhibition of multiple tyrosine kinases but not ALK
Source: PLoS One. 2021 Jul 15;16(7):e0252048. doi: 10.1371/journal.pone.0252048 (PMC8282008; doi:10.1371/journal.pone.0252048)
Supplement: S1 File — (DOCX) [file pone.0252048.s021.docx]

**Supporing Information: Methods**

*Additional methods for MIPE screen*

Brigatinib (ALUNBRIG^®^; CAS: 1197953-54-0), MK-2206 (CAS: 1032350-13-2), dasatinib (SPRYCEL^®^; CAS: 302962-49-8), and simvastatin (ZOCOR^®^; CAS: 79902-63-9) were synthesized by Proactive Molecular Research, Alachua, FL with purities of greater than 98-99% as determined by liquid chromatography-mass spectrometry (LC-MS) and nuclear magnetic resonance (NMR) spectroscopy. ALK-IN-1 (CAS: 1197958-12-5) was obtained from NCATS and Advanced ChemBlocks (Burlingame, CA) and dasatinib-d_8_ (CAS: 1132093-70-9) was purchased from Cayman Chemical (Ann Arbor, MI). For *in vitro* study, all compounds were dissolved in DMSO as 10 mM stock solutions and diluted in medium prior to adding to cells with DMSO concentration kept constant at 0.1%.

*Maximum tolerated dose (MTD) determination, and pharmacokinetic (PK) analysis in NSG mice*

For animal dosing, MK-2206 was dissolved in 30% Captisol^®^ and brigatinib was formulated in 90% polyethylene glycol 300 and 10% 1-methyl-2-pyrrolidinone. The MTD was determined as described (1). Eight-to-12 week-old NSG (NOD-SCID gamma or *NOD.Cg-Prkdc^scid^ Il2rg^tmlWjl^/SzJ*) mice (The Jackson Laboratory) were treated with various doses of MK-2206 every other day or brigatinib every day by oral gavage for two weeks (three mice per dose). The starting doses for MK-2206 and brigatinib were based on those used previously (2,3). Following MTD determination for each drug, a combination of MK-2206 and brigatinib at the MTD was also evaluated to ensure tolerability.

For PK analysis, mice were fed a single oral dose of brigatinib, MK-2206, or their combination at the MTD. Prior to and at various times after dosing (n=3 per time point), whole blood was collected from the facial vein into EDTA-containing Microtainer^®^ (Becton Dickinson). Plasma samples were obtained by centrifugation. Immediately after blood sampling, mice were euthanized for brain harvesting. Both the plasma and brain samples were frozen at -80 ^o^C until analysis of compound concentrations using ultra-high pressure liquid chromatography tandem-mass spectrometry (UHPLC-MS/MS). Plasma and brain samples were deproteinized and were analyzed in the presence of the brigatinib analogue ALK-IN-1 and dasatinib-d_8_ as the internal standards for brigatinib and MK-2206, respectively. Detailed UHPLC-MS/MS analysis is appended in Supplementary Methods.

*Additional methods for Orthotopic Meningioma Model*

The Institutional Animal Care and Use Committee at Nationwide Children’s Hospital approved this animal study. The coordinates used were 1.5 mm anterior and 1.5 mm to the right of the bregma and 4.5 mm below the skull surface. The injected mice were monitored for tumor growth by bioluminescence imaging (BLI) every two weeks using a Xenogen IVIS^®^ Spectrum imaging system (Perkin Elmer, Bradford, CT). Mice with successful tumor engraftment were randomized into four groups (n=10 each) and treated with MK-2206, brigatinib, MK-2206/brigatinib combination, or a mixture of the corresponding vehicles in which MK-2206 and brigatinib were formulated (1:1 ratio) by oral gavage. BLI was performed biweekly to assess the effects on tumor growth. Previously we showed that the BL signal detected in luciferase-expressing tumor xenografts correlates with the tumor size (4). The luminescence detected in each mouse was normalized to its pretreatment signal and expressed as the mean normalized luminescence ± standard deviation for each treatment group. After 14 weeks of treatment, treatment was halted in a subset of mice in the brigatinib alone and MK-2206/brigatinib combination cohorts and monitored possible tumor regrowth for 12 more weeks. Then, treatment was reinitiated to determine tumor response.

*Meningioma Model Immunohistochemistry*

The heads of drug- or vehicle-treated mice bearing meningiomas were decalcified, followed by paraffin embedding as described previously (4). Serial 5-µm sections were cut and stained with hematoxylin and eosin to localize the tumor. Then, the sections containing tumors were processed for immunohistochemical staining using antibodies against Ki67 (RM-9106-S, Neomarkers), phospho-S6 (p-S6[Ser^235/236^]; #4858, Cell Signaling), p-ERK1/2(Thr^202^/Tyr^204^) (#4370, Cell Signaling), and cleaved caspase 3 (CC3; #9664, Cell Signaling). Negative control slides were treated with the same procedures but without the primary antibody.

*Additional methods for* in vivo *Schwannoma Model*

Indiana University’s Institutional Animal Care and Use Committee approved this study. *Postn-Cre; Nf2^flox/flox^* mice were divided into treatment groups and administered drug by oral gavage. The number of animals used in analysis for each treatment group is as follows: Dasatinib (n=14), Simvastatin (n=15), Brigatinib (n=10), Dasatinib/Simvastatin (n=14), Dasatinib/Brigatinib (n=14), Vehicle Controls (n=37). Drugs were formulated as follows and administered by oral gavage: 50 mg/kg brigatinib dissolved in 90% PEG400 with 10% 1-metyl-2-pyrrolidinone; 20mg/kg dasatinib in pH3.0 citrate buffer; and 100 mg/kg simvastatin in 1% carboxymethylcellulosein water with 0.25% Tween80 and 0.05% antifoam 204 (Sigma). Tissue processing and DRG volume quantification methods were carried out as previously described (29). Dissected nerve trees were pre-embedded in 2% agar, processed in a Leica tissue processor through graded alcohols, xylenes, and finally in molten paraffin. Five-micron thick sections were cut on a Leica rotary microtome and mounted on charged slides, then stained with hematoxylin and eosin. Images were acquired with an Aperio CS2 slide scanner (Leica).

*PK analysis of brigatinib and dasatinib in plasma samples of PostnCre; Nf2^foxl/flox^ mice*

HPLC/MS was performed by the IU Simon Cancer Center’s Clinical Pharmacology Analytical Core. Samples were acidified and extracted in hexane:ethyl acetate (50:50, v/v). After solvent evaporation, mobile phase (acetonitrile:5mM ammonium acetate; 70:30, v/v) was mixed with residual sample and injected into an Agilent 1290 HPLC system with an Eskigent Autosampler. Mass spectrometry was performed using an ABSciex 5500 Q-TRAP.

*Phospho-receptor tyrosine kinase (RTK) array*

Ben-Men-1 cells were plated in Dulbecco modified Eagle medium (DMEM) containing 10% fetal bovine serum (FBS). The following day, cells were growth-arrested in medium without serum for two days. Serum-starved cells were stimulated with 20% FBS-containing medium in the presence of 1x IC_50_ concentration of brigatinib or DMSO vehicle. Also, cells grown in 10% FBS were treated with 1x IC_50_ concentration of brigatinib or DMSO in fresh 10% FBS-containing medium for 2 or 24 hours. Treated cells were lysed in 500 µL of 1x Cell Lysis buffer (#9803; Cell Signaling, Danvers, MA) with 1 mM freshly-added PMSF. Cell lysates were sonicated and their protein concentrations were assessed by microBCA assay (#23235; Thermo, Rockford, IL). 150 µg of total protein was added to each chamber of the PathScan RTK Antibody Array (#7949; Cell Signaling) according to the manufacturer’s instructions. The fluorescent signal of the array was detected using an Odyssey CLx near-infrared scanner (LI-COR, Lincoln, NE), followed by quantitation using Image Studio software (LI-COR). Also, the remaining protein lysates from the above treated cells were analyzed by Western blotting to validate array findings.

*Meningioma Cells Western blot analysis*

Ben-Men-1 cells were growth-arrested in serum-free DMEM for two days and then pretreated for 2 hours with 1.5 µM of brigatinib or DMSO vehicle, followed by incubation for 10 minutes with 50 ng/mL each of the following ligands: epidermal growth factor (EGF) (#PHG0311; Thermo Fisher, Waltham, MA), heregulin (HRG) (#100-03; PeproTech, Rocky Hill, NJ), or insulin-like growth factor 1 (IGF1) (#1150-01; Gold Biotechnology, St. Louis, MO). Following ligand stimulation, cells were placed on ice, lysed in cold RIPA buffer containing protease/phosphatase inhibitor cocktail (#78440; Sigma-Aldrich, St. Louis, MO), and sonicated. Also, lysates were prepared from primary NF2-associated and sporadic meningioma, Ben-Men-1, normal human meningeal (ScienCell, Carlsbad, CA), and SK-N-SH neuroblastoma cells. Equal amounts of protein lysates were used in Western blotting as previously described (4).

*Drug concentrations used for Figure 2 (also used for Fig S2A-C).*

For meningioma-related cells lines (Fig 2C), single drug dose response testing was carried out for MK-2206 or brigatinib at a concentration range of 0.0015 – 10 µM (3-fold dilutions seris, 9 dosage points) and DMSO (vehicle) control. For combination drug testing, cells were treated in a 10x10 dose matric format using same concentrations listed above.

*Additional methods for RNAseq and transcriptome analysis*

RNAseq reads were aligned to human reference genome Ensembl GRCh37 (v.75) using STAR (v. 2.5.2a) (5) with parameters ‘–outSAMunmapped Within –outFilterMultimapNmax 1 –outFilterMismatchNoverLmax 0.1 –alignIntronMin 21 –alignIntronMax 0 –alignEndsType Local –quantMode GeneCounts –twopassMode Basic’. In this step, STAR also generated gene level counts for all libraries. Quality checking of alignments was assessed by a custom script utilizing Picard Tools (http://broadinstitute.github.io/picard/), RNASeQC (6), RSeQC (7) and samTools (8). These analyses identified two samples, Syn1_1, and Syn1_11 (Brigatinib), as outlier samples as they failed to pass the following thresholds: exonic rate ≥ 0.8 and intergenic rate ≤ 0.1, and thus they were excluded from further analysis. Differentially expressed genes in pair-wise comparisons were identified by edgeR’s quasi-likelihood F test (v. 3.18.1) (9), which was run at the R platform (v. 3.4) on genes with greater than 10 counts across replicates per condition in pair-wise comparisons. Gene ontology (GO) enrichment analysis for each comparison was performed on differentially expressed genes at Bonferroni adjusted p values < 0.05, where all the analyzed genes in a given comparison were used as a background list, using DAVID (v. 6.8) (10). GO terms with Benjamini Hochberg adjusted p values < 0.05 were reported as significantly enriched for a given gene list.

*Western blot of drug-treated DRG tumor samples from Postn-Cre;Nf2^flox/flox^* mice

DRG tumor samples derived from mice shown in Figure 6 were collected at indicated time points after single dose oral gavage of indicated drugs. 9 DRGs for Dasatinib+Brigatinib treatment, 12 for all other conditions were collected in ice cold lysis buffer (1% IGEPAL; 0,1% DOC; 100 mM NaCl; 50 mM Tris pH 8.0) containing phosphatase and protease inhibitors (PhosSTOP, #04906837001 and cOmplete EDTA-free protease inhibitor cocktail, #4693132001, both from Roche). Lysis was performed in a Precellys 24 device (Bertin Technologies) at 2 x 5000 rpm for 30s with 5s break in-between. Cellular debris was removed twice by centrifugation at 12.000 rpm for 10 min and supernatant collected. Protein concentration was determined using a BCA protein assay kit (#23225; ThermorFisher). 10 µg each were separated by SDS-PAGE and transferred onto nitrocellulose membranes. Membranes were blocked in 5% milk/TBST and probed at 4°C overnight with the following primary antibodies in blocking buffer: p-AKT (Ser^473^) (#4060), ERK1/2 (#9102), p-ERK1/2 (Thr^202^/Tyr^204^) (#4696), p-FAK (Tyr^397^) (#8556), GSK (#9832), p-GSK (Ser^9^) (#8213), IGF1R-beta (#9750), p-IGF1R-beta (Tyr^1135^/Tyr^1136^) (#3024), MEK1/2 (#8727), p-MEK1/2 (Ser^217^/Ser^221^), p-p70S6K (Thr^389^) (#9234), S6RP (#2317), p-S6RP (Ser^235^/Ser^236^) (#4858), p-Stat1 (Tyr^701^) (#7649), Stat3 (#9139), p-Stat3 (Tyr^705^) (#9145) [all from Cell Signaling], Pyk2 (#ab32571; Abcam), AKT1/2/3 (#sc-8312), FAK (#sc-271126) (both from Santa Cruz Biotechnology). Membranes were washed three times for 10 min each in TBST before probing with following secondary antibodies in blocking buffer for 1h at room temperature: Chemiluminescent signals were generated using ECL (#32106) or ECL+ (#32132) (both ThermoFisher) following manufacturer’s instructions and detected on Super RX-N films (#4141019289; Fuji).

**Supplemental Methods References**

1. Plowman J CR, Alley M, Sausville E, Schepartz S. US-NCI testing procedures. Relevance of Tumor Models for Anticancer Drug Development: Karger; 1999. p 121-35.

2. Hirai H, Sootome H, Nakatsuru Y, Miyama K, Taguchi S, Tsujioka K*, et al.* MK-2206, an allosteric Akt inhibitor, enhances antitumor efficacy by standard chemotherapeutic agents or molecular targeted drugs i*n vitro* and *in vivo*. Mol Cancer Ther 2010;9:1956-67

3. Siaw JT, Wan H, Pfeifer K, Rivera VM, Guan J, Palmer RH*, et al.* Brigatinib, an anaplastic lymphoma kinase inhibitor, abrogates activity and growth in ALK-positive neuroblastoma cells, Drosophila and mice. Oncotarget 2016;7:29011-22

4. Burns SS, Akhmametyeva EM, Oblinger JL, Bush ML, Huang J, Senner V*, et al.* Histone deacetylase inhibitor AR-42 differentially affects cell-cycle transit in meningeal and meningioma cells, potently inhibiting NF2-deficient meningioma growth. Cancer Res; 2013; 73:792-803

5. Dobin, A., Davis, C.A., Schlesinger, F., Drenkow, J., Zaleski, C., Jha, S., Batut, P., Chaisson, M., and Gingeras, T.R. STAR: ultrafast universal RNA-seq aligner. Bioinformatics; 2013; 29, 15-21.

6. DeLuca, D.S., Levin, J.Z., Sivachenko, A., Fennell, T., Nazaire, M.D., Williams, C., Reich, M., Winckler, W., and Getz, G. RNA-SeQC: RNA-seq metrics for quality control and process optimization. Bioinformatics; 2012; 28, 1530-1532.

7. Li, H., Handsaker, B., Wysoker, A., Fennell, T., Ruan, J., Homer, N., Marth, G., Abecasis, G., Durbin, R., and Genome Project Data Processing, S. The Sequence Alignment/Map format and SAMtools. Bioinformatics; 2009; 25, 2078-2079.

8. Wang, L., Wang, S., and Li, W. RSeQC: quality control of RNA-seq experiments. Bioinformatics; 2012; 28, 2184-2185.

9. Robinson, M.D., McCarthy, D.J., and Smyth, G.K. edgeR: a Bioconductor package for differential expression analysis of digital gene expression data. Bioinformatics; 2010; 26, 139-140.

10. Huang da, W., Sherman, B.T., and Lempicki, R.A. Systematic and integrative analysis of large gene lists using DAVID bioinformatics resources. Nat Protoc; 2009; 4, 44-57.
