# Supplementary material for: Brigatinib causes tumor shrinkage in both NF2-deficient meningioma and schwannoma through inhibition of multiple tyrosine kinases but not ALK
Source: PLoS One. 2021 Jul 15;16(7):e0252048. doi: 10.1371/journal.pone.0252048 (PMC8282008; doi:10.1371/journal.pone.0252048)
Supplement: S1 Raw images — (PDF) [file pone.0252048.s022.pdf]

Raw image files for Chang et al.

Figure 4A

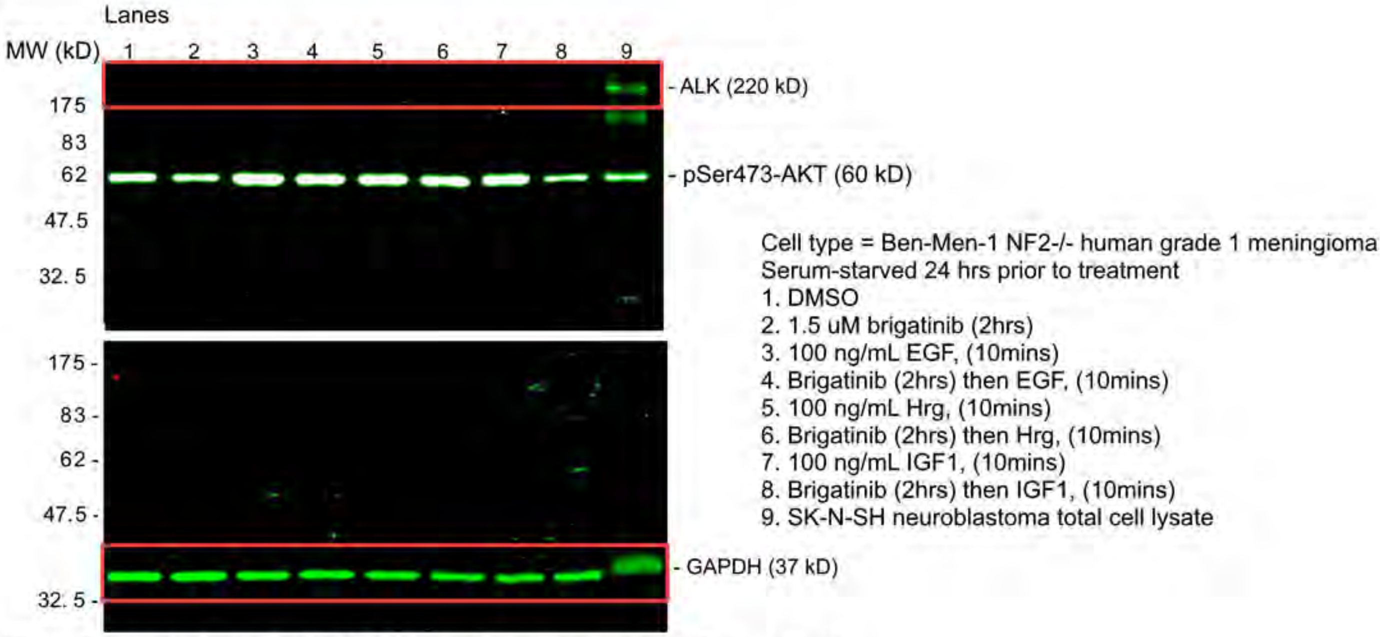

Cell type = Ben-Men-1 NF2-/- human grade 1 meningioma
Serum-starved 24 hrs prior to treatment

1. DMSO
2. 1.5 uM brigatinib (2hrs)
3. 100 ng/mL EGF, (10mins)
4. Brigatinib (2hrs) then EGF, (10mins)
5. 100 ng/mL Hrg, (10mins)
6. Brigatinib (2hrs) then Hrg, (10mins)
7. 100 ng/mL IGF1, (10mins)
8. Brigatinib (2hrs) then IGF1, (10mins)
9. SK-N-SH neuroblastoma total cell lysate

Digital image files acquired using the LI-COR Odyssey CLx scanner and ImageStudio software
Manual scan settings: 84 micron resolution, medium quality

Protein of interest is indicated by a red box with label + molecular weight to the right
Approximate MW ladder is indicated to the left
Other proteins probed on blots are indicated by label+MW to the right

Figure 4C

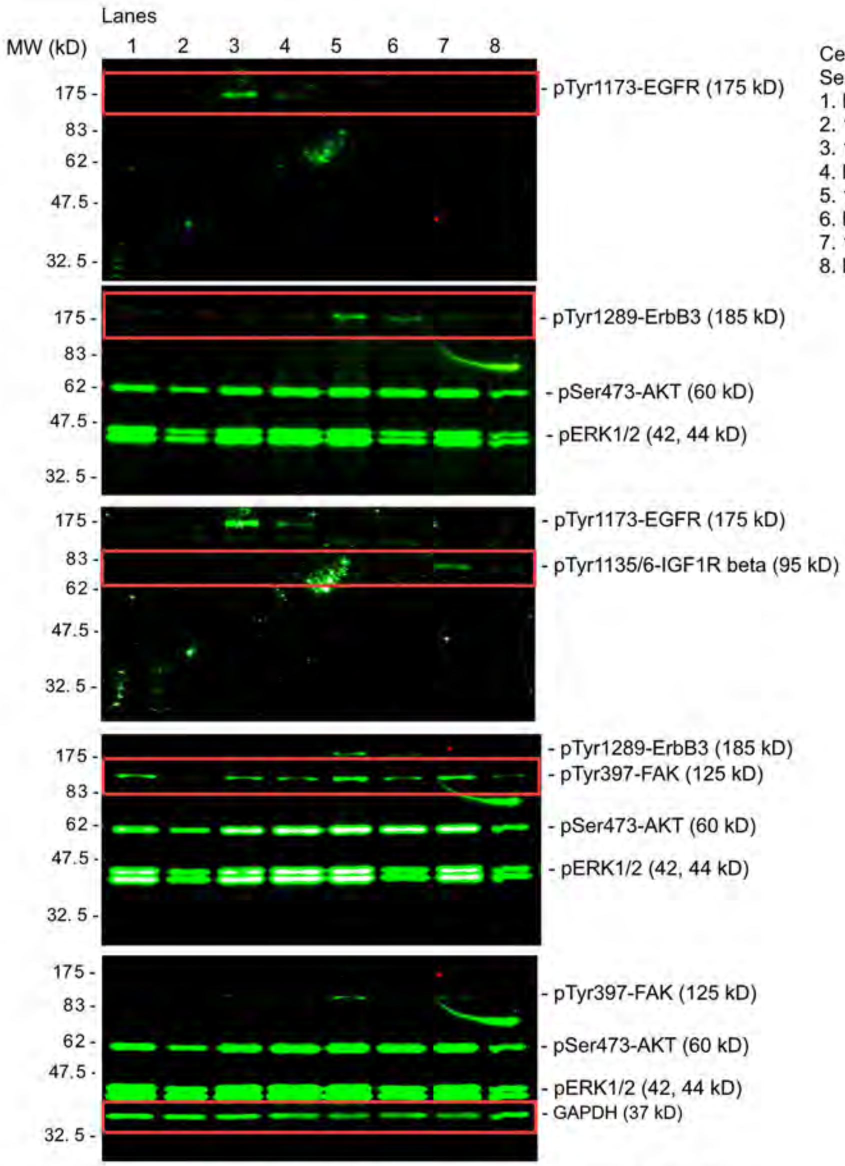

Cell type = Ben-Men-1 NF2-/- human grade 1 meningioma
Serum-starved 24 hrs prior to treatment
1. DMSO
2. 1.5 uM brigatinib (2hrs)
3. 100 ng/mL EGF (10mins)
4. Brigatinib (2hrs) then EGF (10mins)
5. 100 ng/mL Hrg (10mins)
6. Brigatinib (2hrs) then Hrg (10mins)
7. 100 ng/mL IGF1, (10mins)
8. Brigatinib (2hrs) then IGF1 (10mins)

Digital image files acquired using the LI-COR Odyssey CLx scanner and ImageStudio software
Manual scan settings: 84 micron resolution, medium quality
Protein of interest is indicated by a red box with label + molecular weight to the right
Approximate MW ladder is indicated to the left
Other proteins probed on blots are indicated by label+MW to the right

Figure 4D

Effect of Brigatinib in schwann cell line

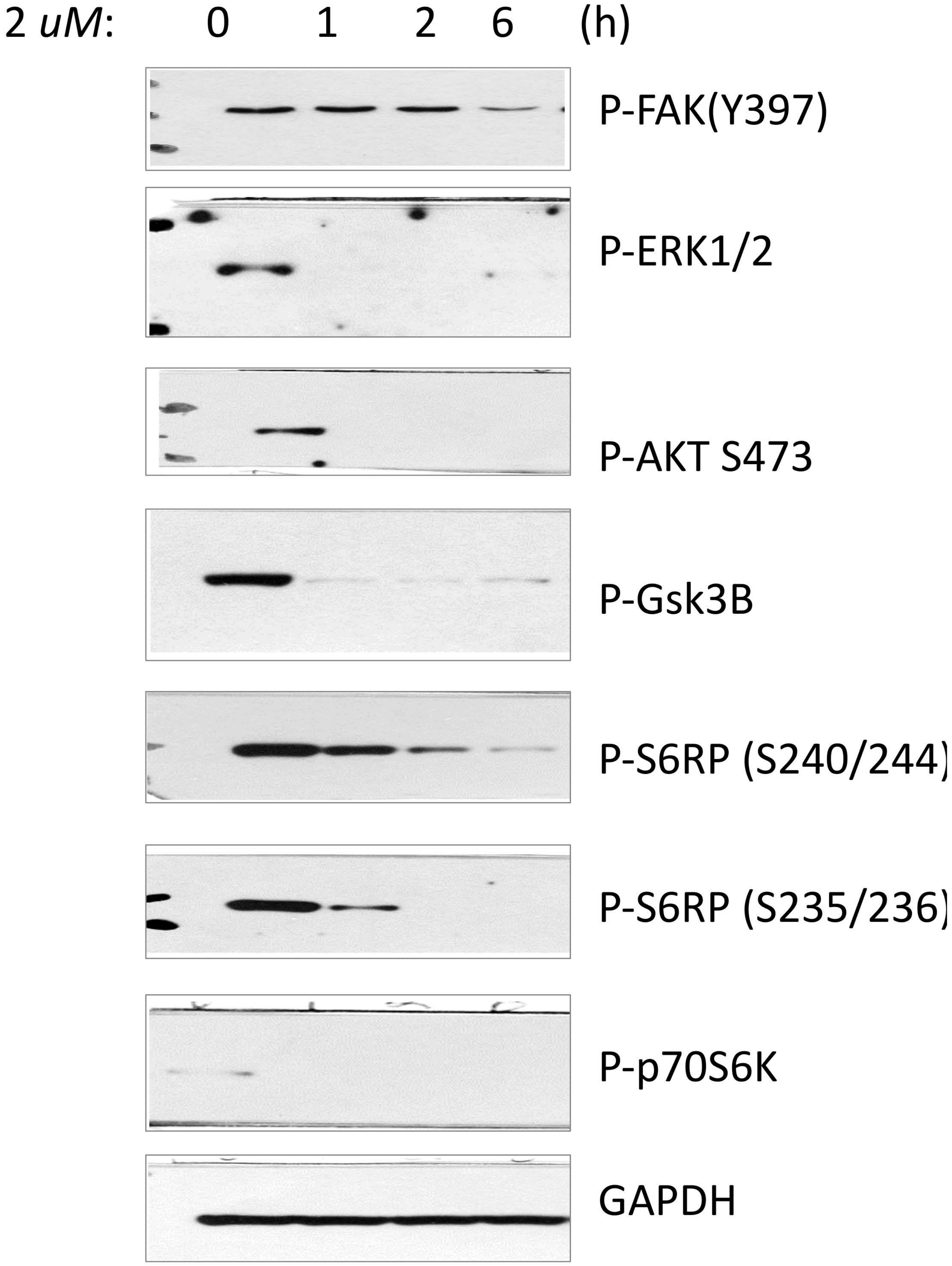

Fig 4E

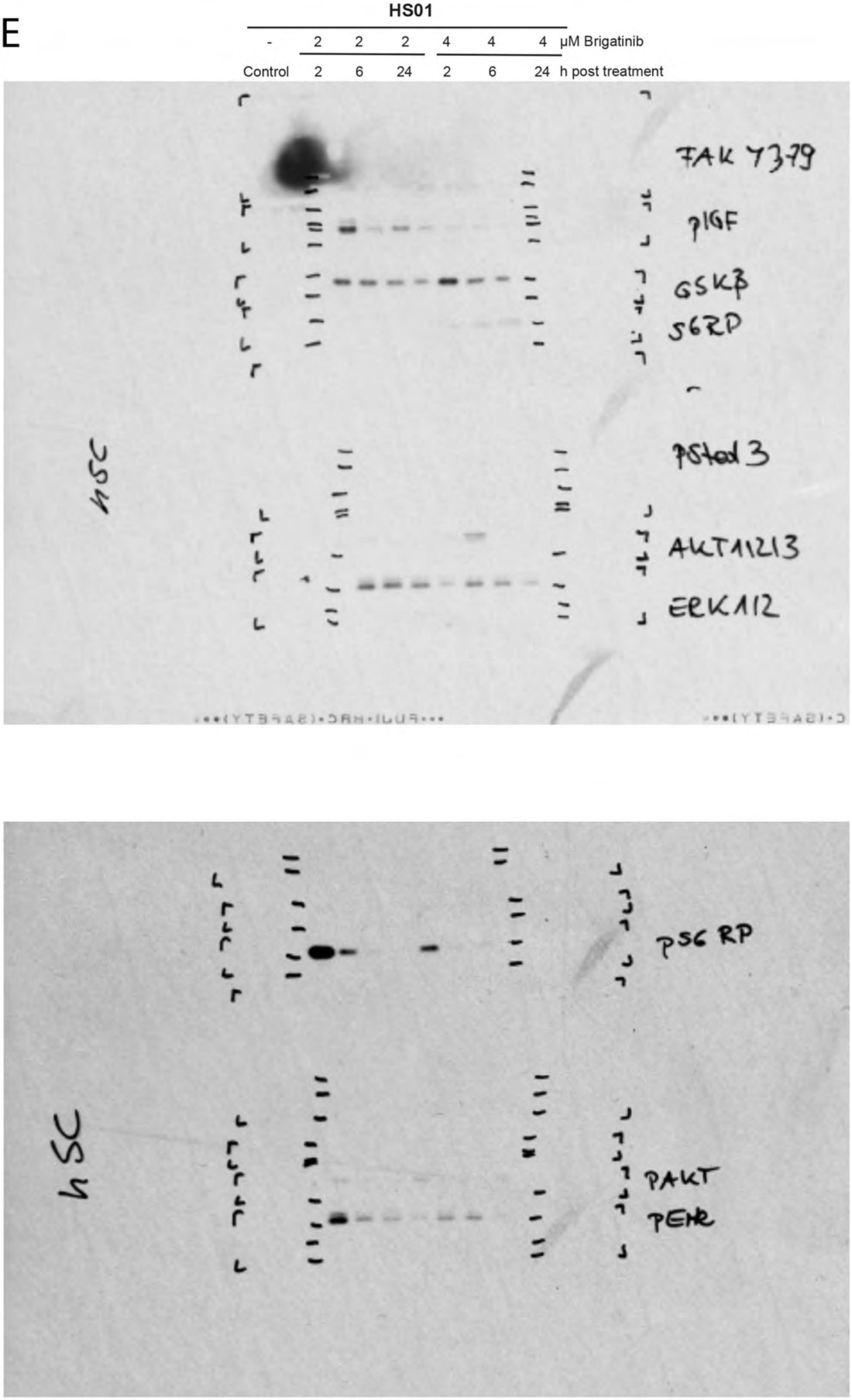
HS01
- 2 2 2 4 4 4 µM Brigatinib
Control 2 6 24 2 6 24 h post treatment

Fig 4E cont.

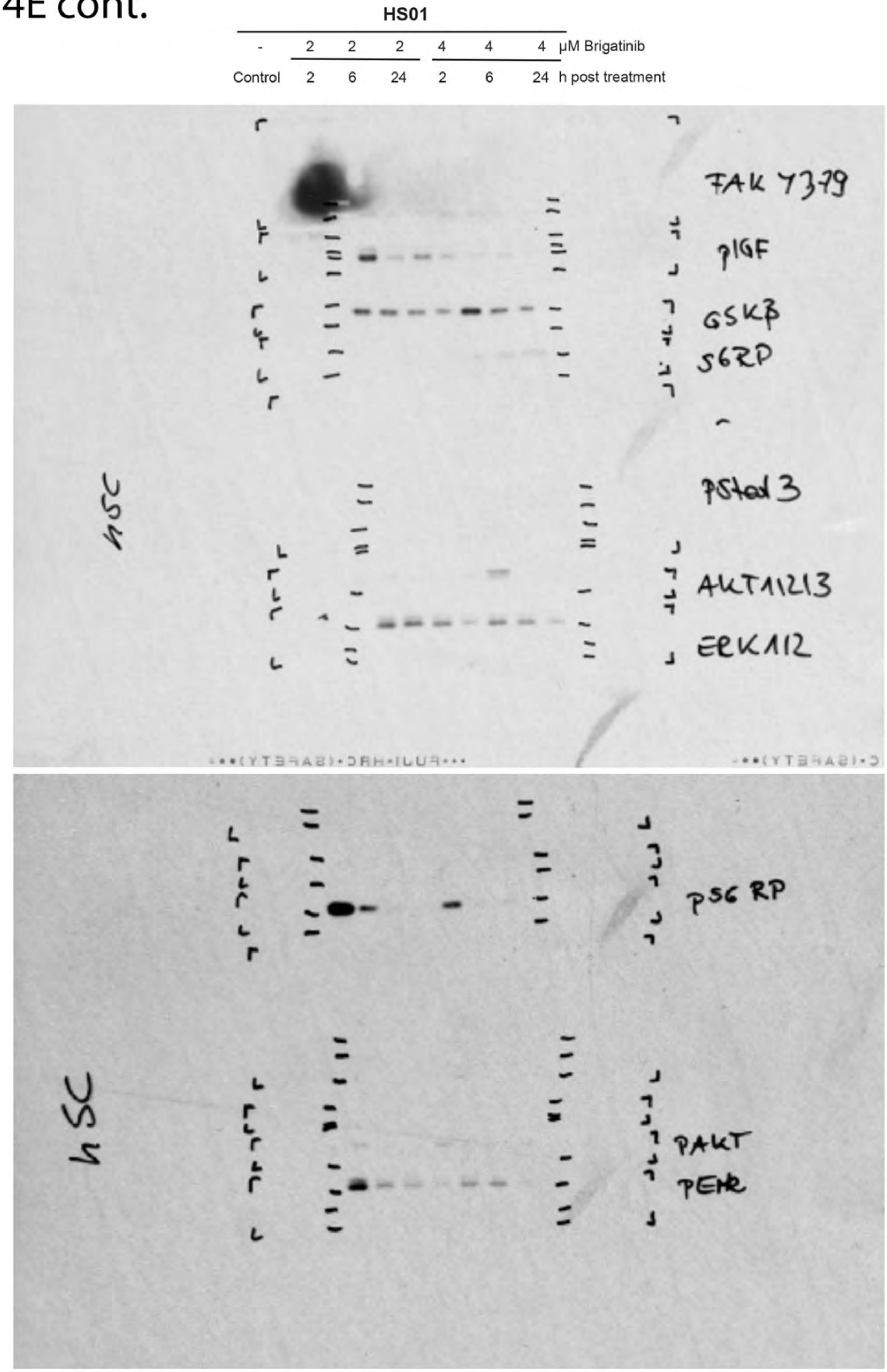
HS01
- 2 2 2 4 4 4 µM Brigatinib
Control 2 6 24 2 6 24 h post treatment
FAK Y379
pIGF
GSKβ
S6RP
pStat3
AKT1/2/3
ERK1/2
hSC
pS6 RP
pAKT
pERK
hSC

Fig 4E cont.

HS01							
-	2	2	2	4	4	4	µM Brigatinib
Control	2	6	24	2	6	24	h post treatment

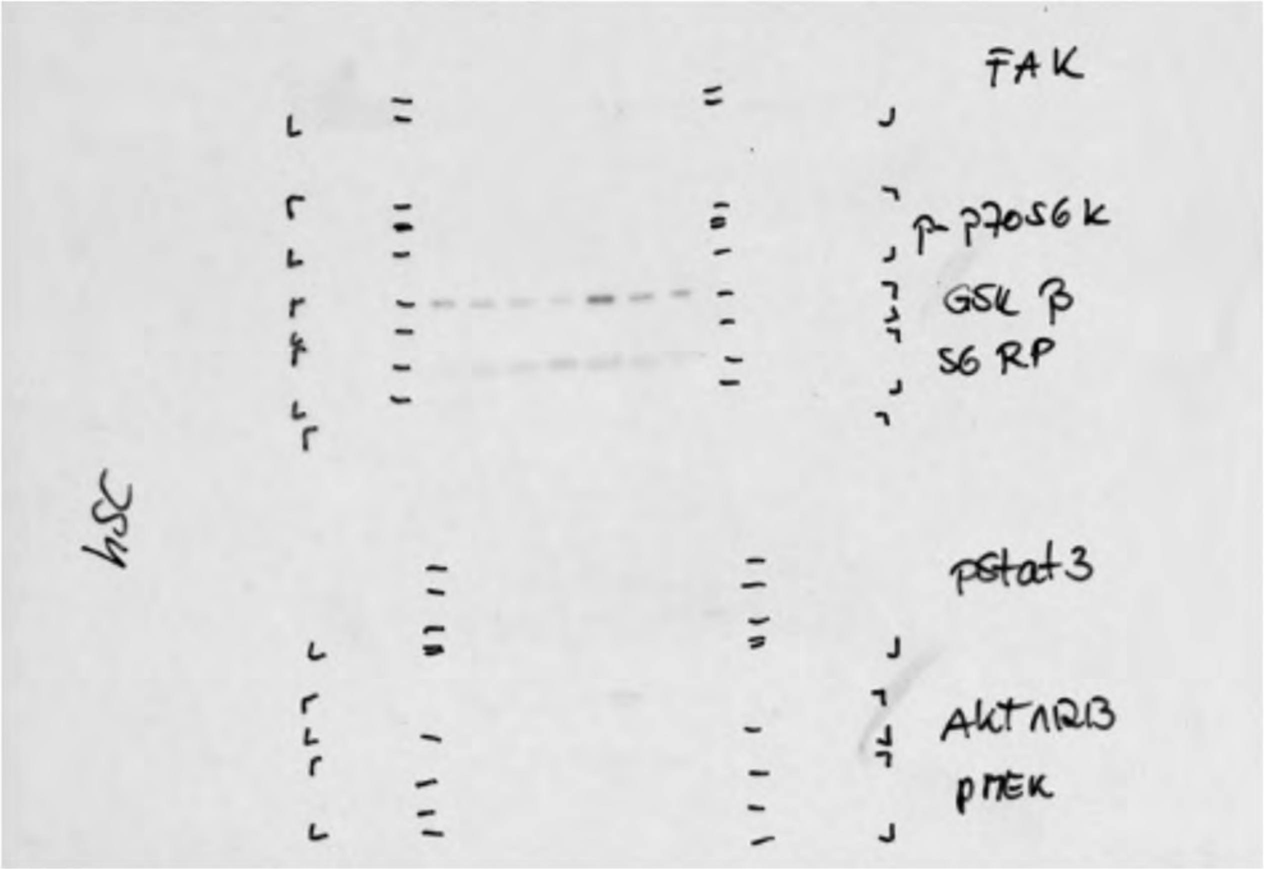

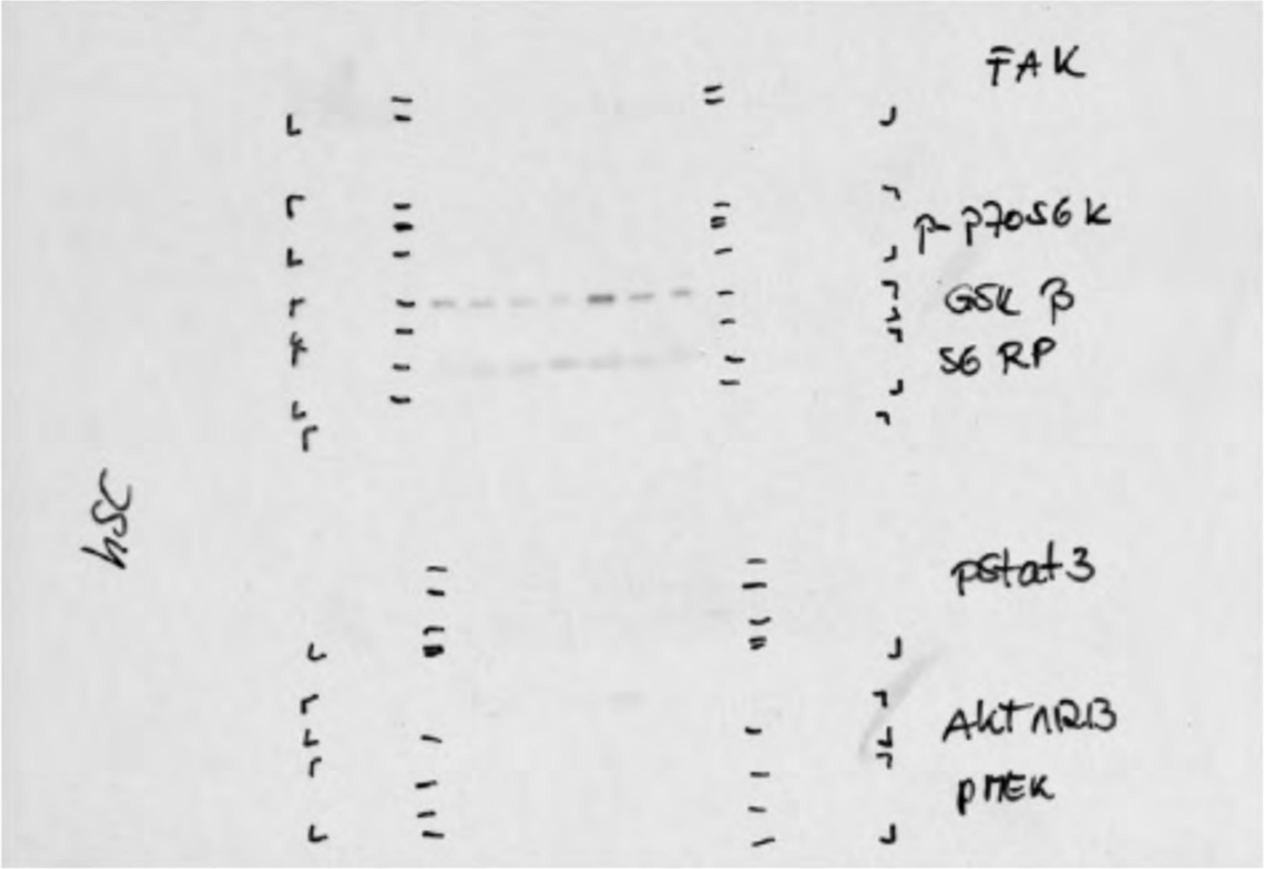

Fig 4E cont.

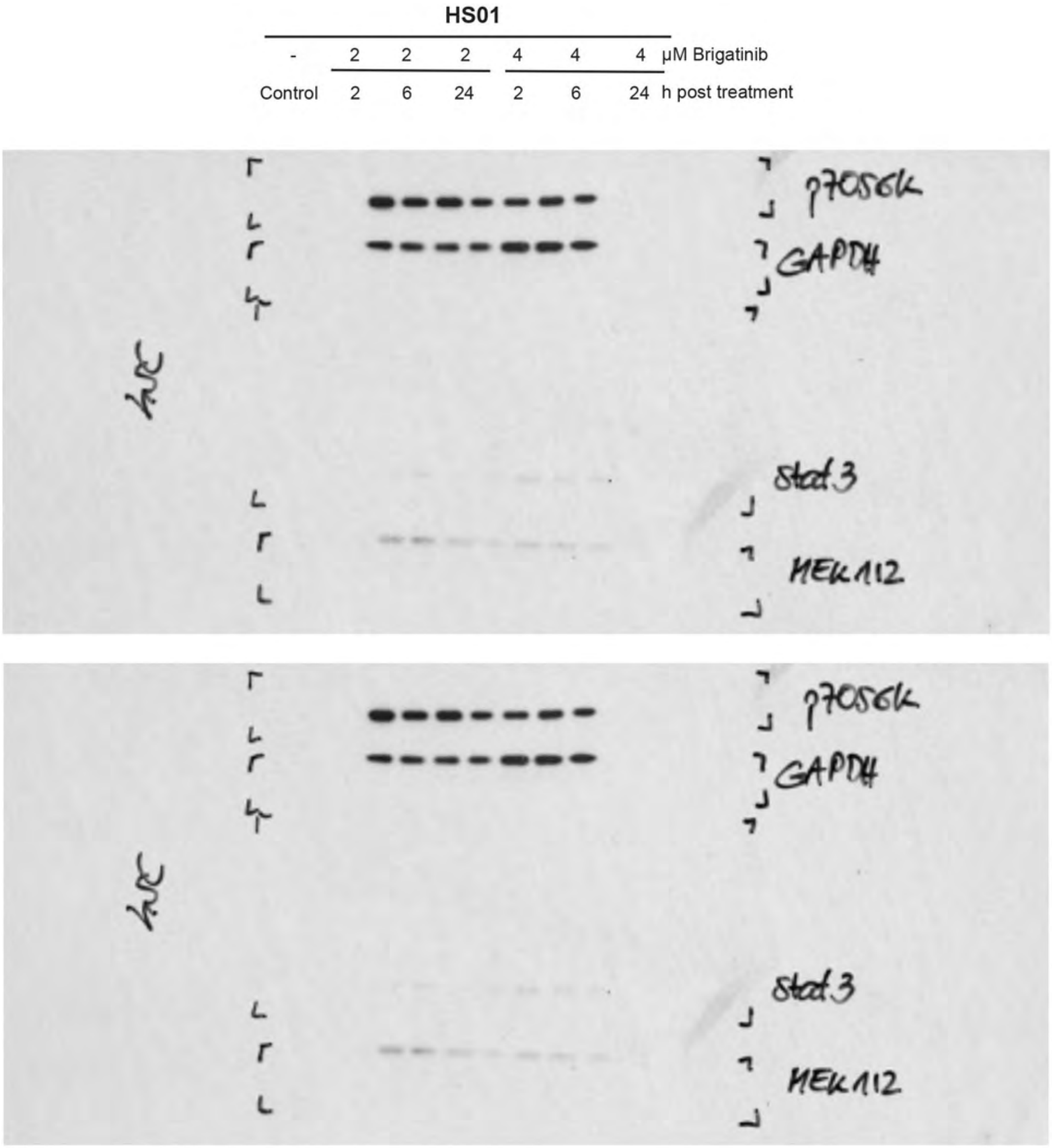
HS01
- 2 2 2 4 4 4 µM Brigatinib
Control 2 6 24 2 6 24 h post treatment
p70S6k
GAPDH
Stat3
MEK1/2
p70S6k
GAPDH
Stat3
MEK1/2

Fig 4E cont. Ponceau Loading Control

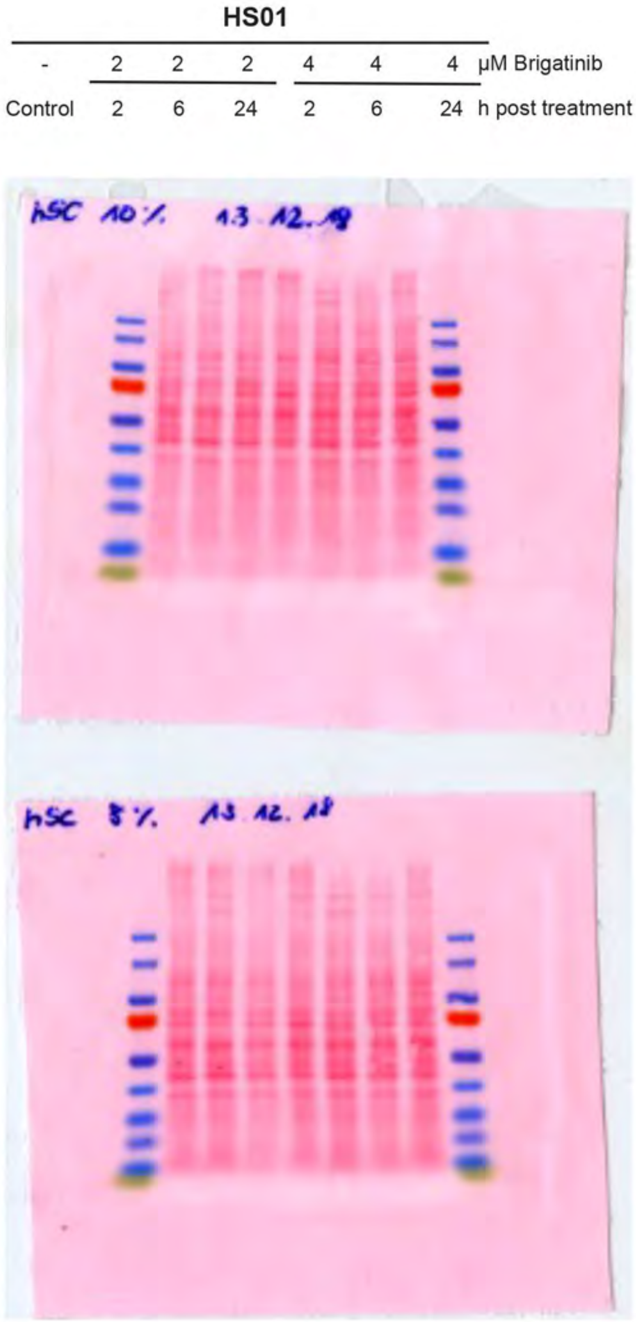

Supplementary Figure S10A

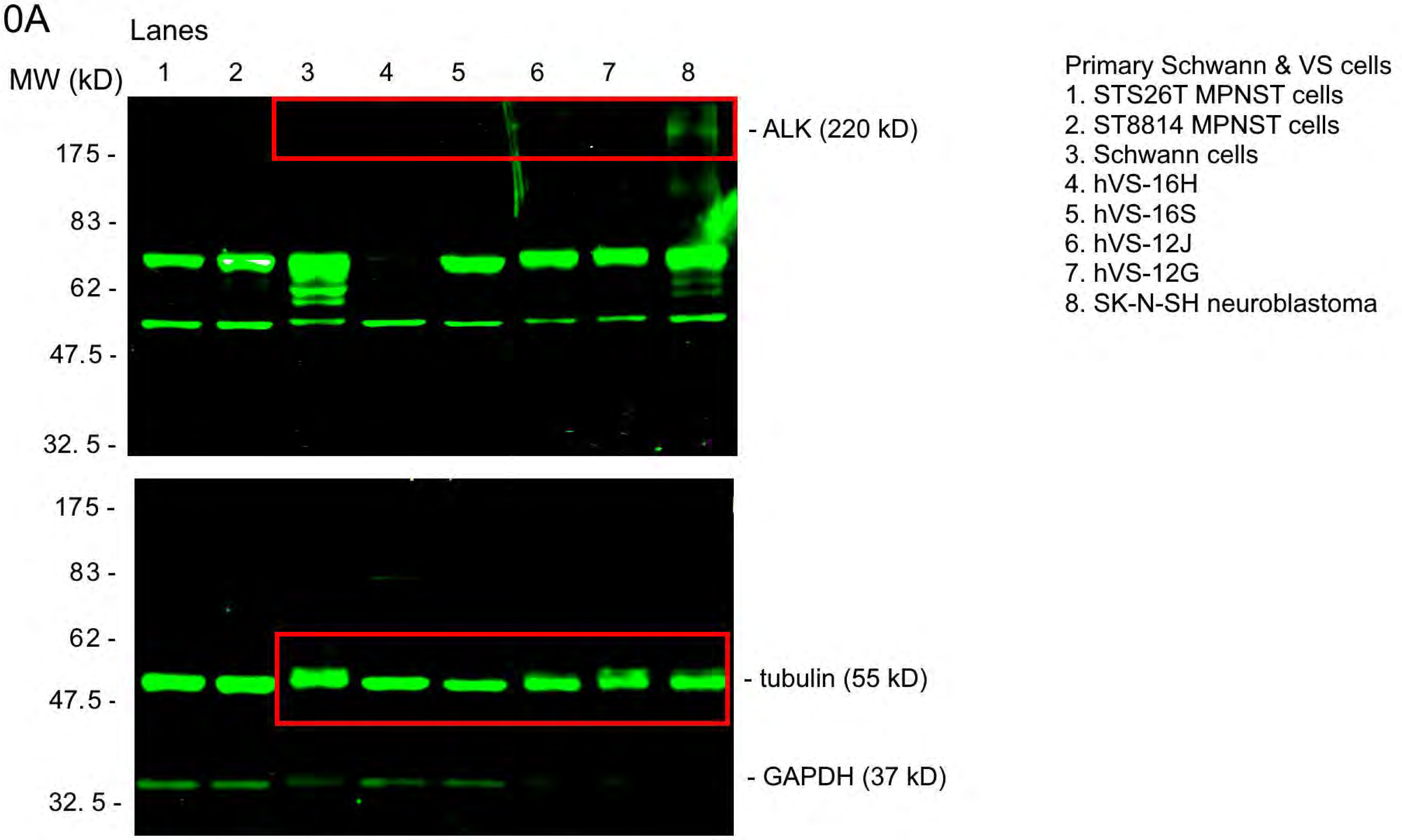

Supplementary Figure S10B

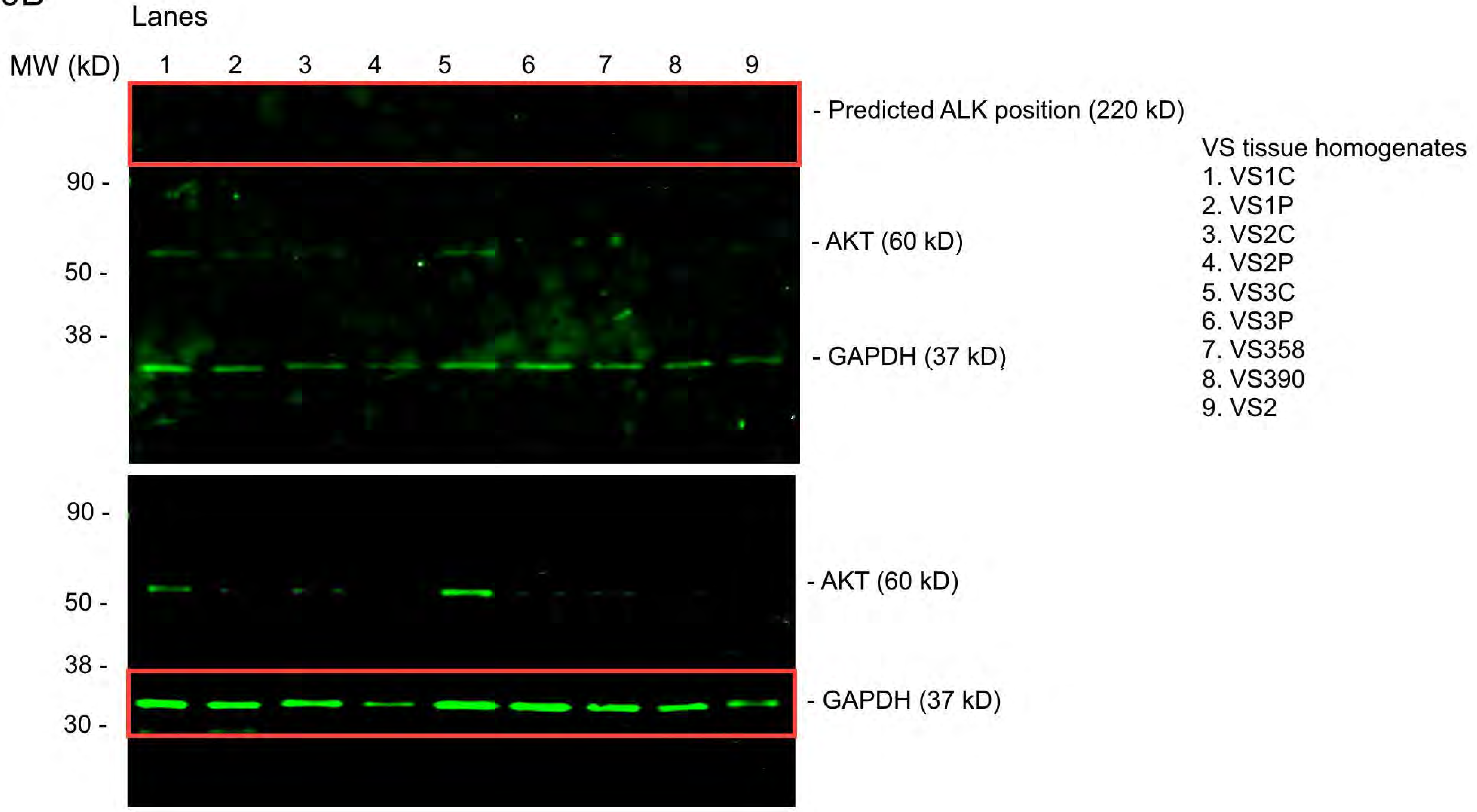

Digital image files acquired using the LI-COR Odyssey CLx scanner and ImageStudio software
Manual scan settings: 84 micron resolution, medium quality
Protein of interest is indicated by a red box with label + molecular weight to the right
Approximate MW ladder is indicated to the left
Other proteins probed on blots are indicated by label+MW to the right

Supplementary Figure S7

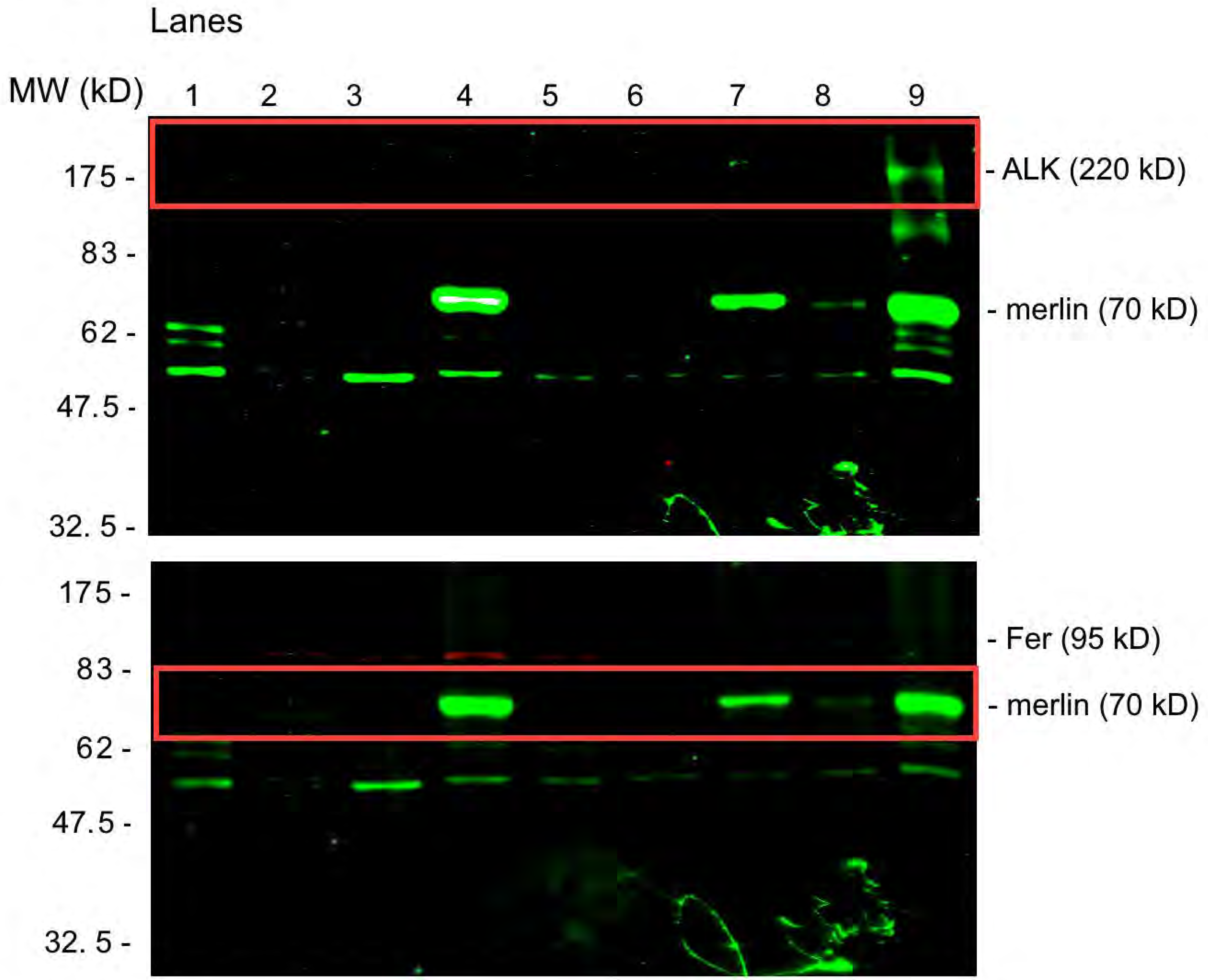

Meningioma lines/primary cultures & meningeal cells
1. KT21-MG1-Luc
2. AG-NF2-Men
3. Ben-Men-1
4. Meningeal cells
5. hMen-10A
6. hMen-10B
7. hMen-12Ao
8. hMen-12B
9. SK-N-SH neuroblastoma

Digital image files acquired using the LI-COR Odyssey CLx scanner and ImageStudio software
Manual scan settings: 84 micron resolution, medium quality
Protein of interest is indicated by a red box with label + molecular weight to the right
Approximate MW ladder is indicated to the left
Other proteins probed on blots are indicated by label+MW to the right

Supplementary Figure S9

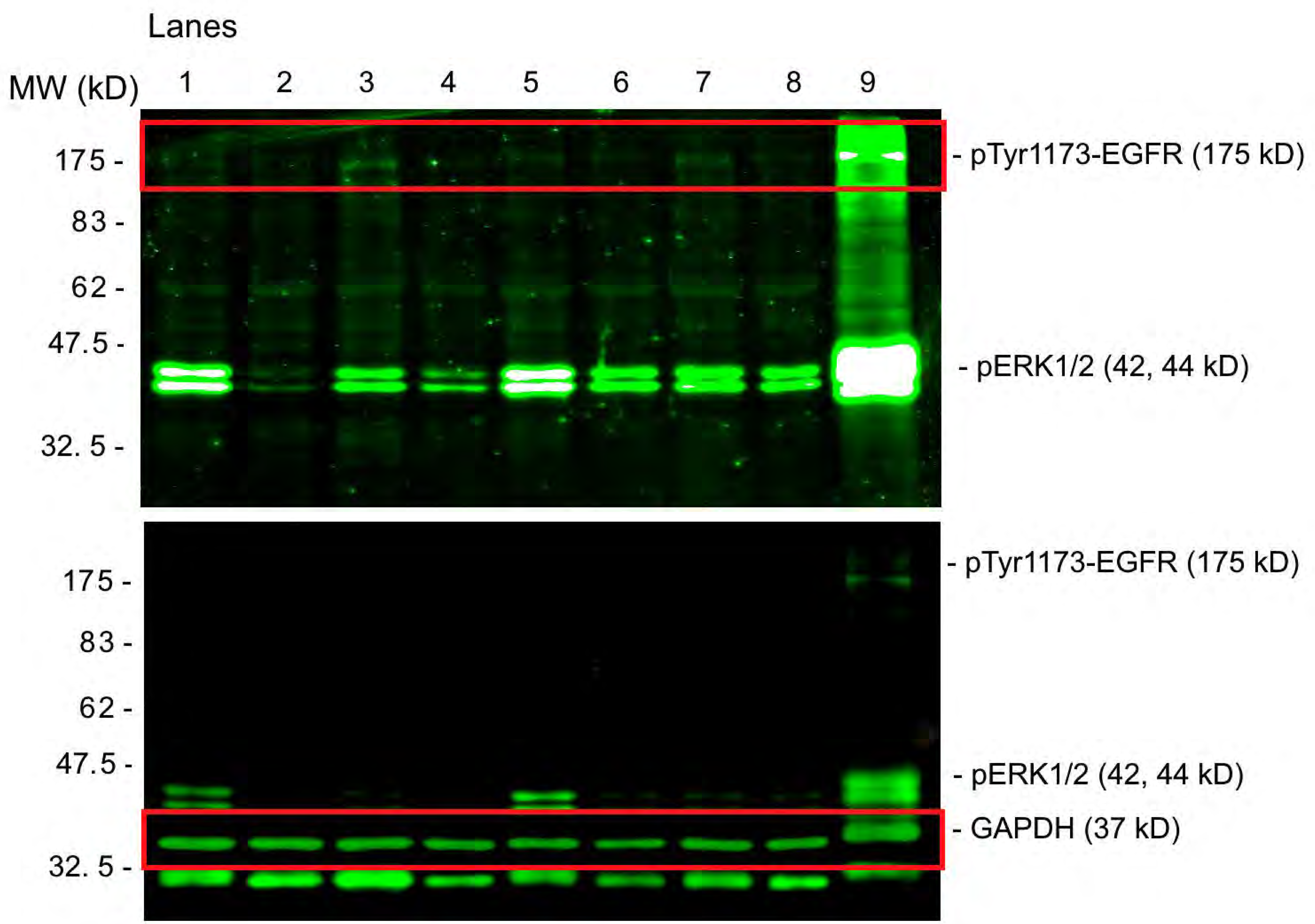

Cell type = Ben-Men-1 NF2-/- human grade 1 meningioma
Lane assignments:
1. 10% FBS 2h + DMSO
2. 10% FBS 2h + brigatinib
3. 10% FBS 24h + DMSO
4. 10% FBS 24h + brigatinib
5. 20% FBS 2h + DMSO
6. 20% FBS 2h + brigatinib
7. 20% FBS 24h + DMSO
8. 20% FBS 24h + brigatinib
9. 100 ng/mL EGF (10 mins)

Digital image files acquired using the LI-COR Odyssey CLx scanner and ImageStudio software
Manual scan settings: 84 micron resolution, me [illegible] - pERK1/2 (42, 44 kD)
Protein of interest is indicated by a red box with label + molecular weight to the right
Approximate MW ladder is indicated to the left
Other proteins probed on blots are indicated by label+MW to the right

The remaining images are for Figure S13

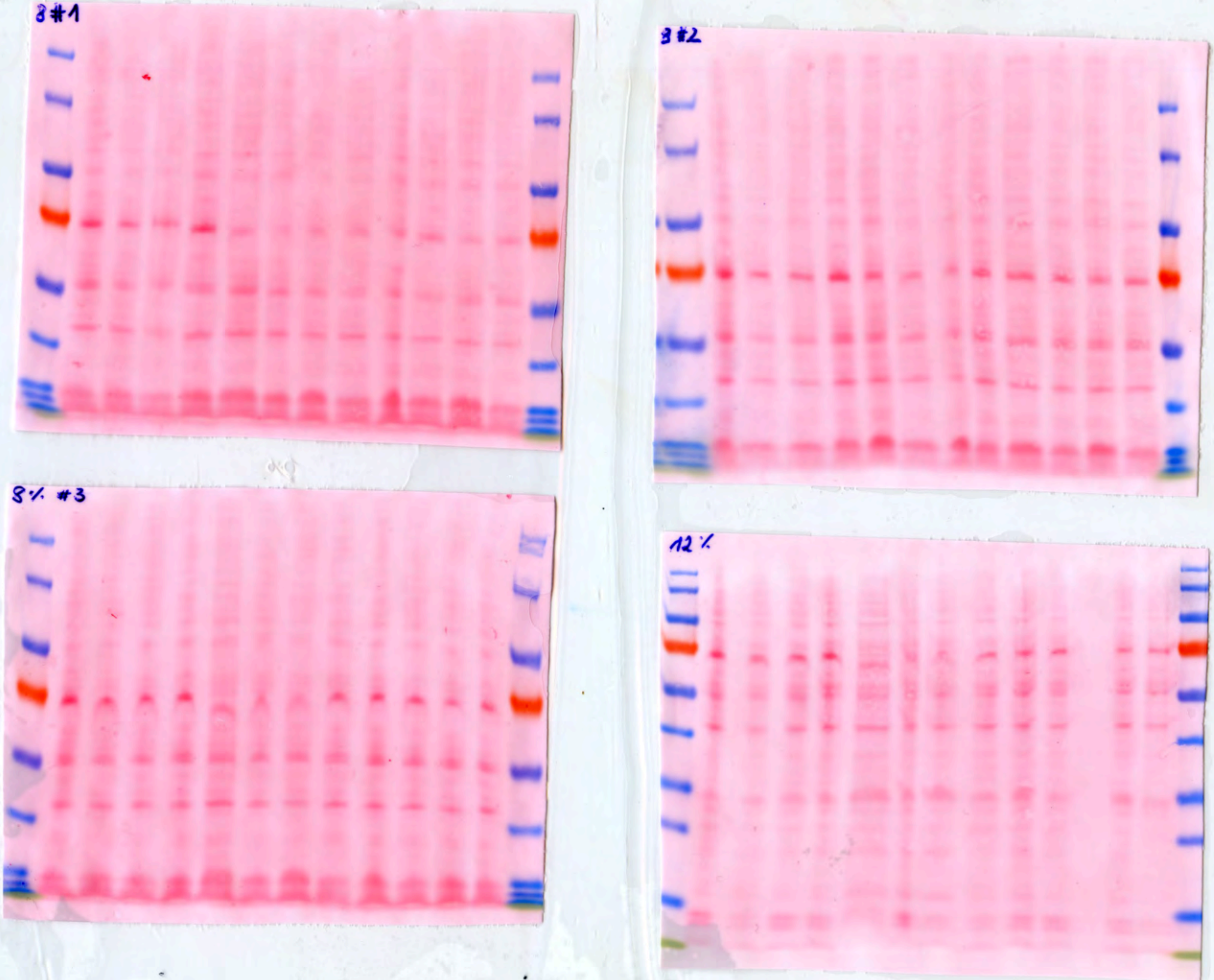
8 #1
8 #2
8% #3
12%

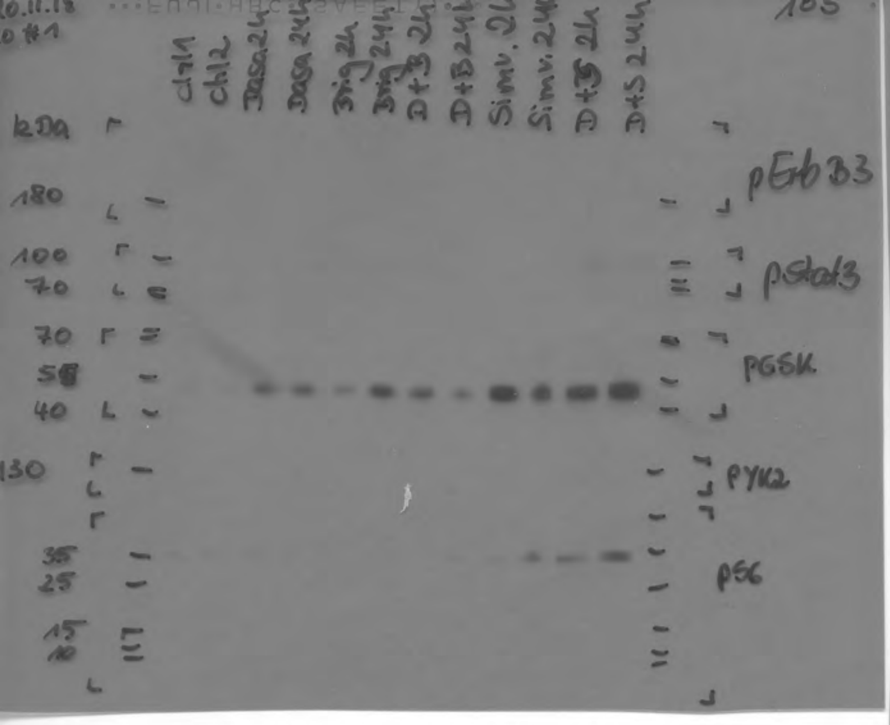
20.11.18
10#1
105
chl1 chl2 Dasa2h Dasa24h Brig2h Brig24h D+B 2h D+B 24h Simv.2h Simv.24h D+S 2h D+S 24h
kDa
180
100
70
70
55
40
130
35
25
15
10
pErbB3
pStat3
pGSK
PYK2
pS6

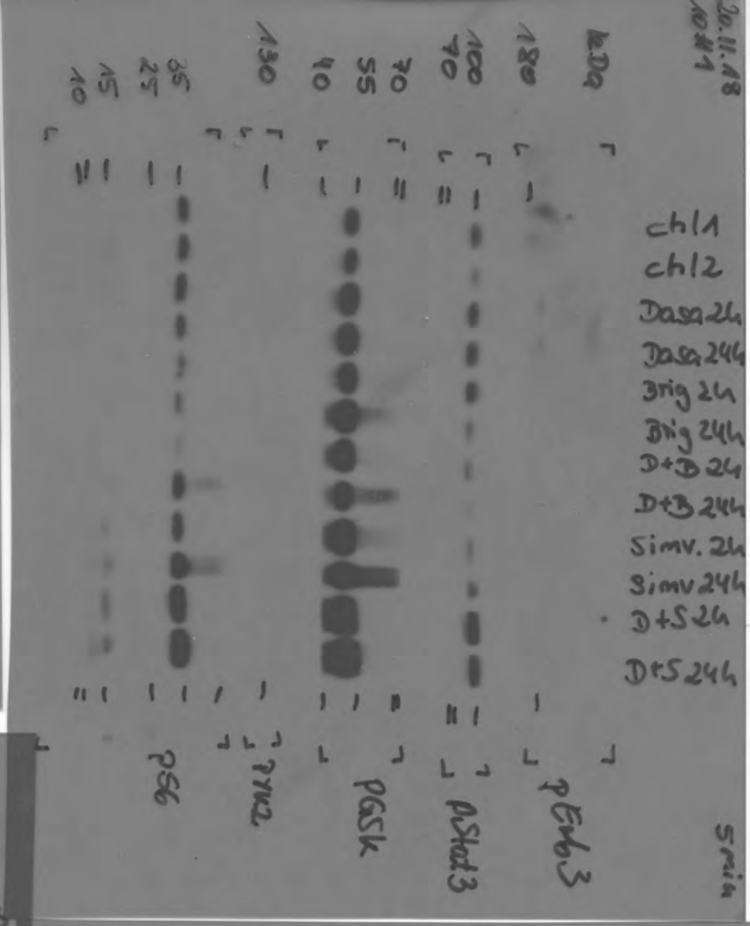
20.11.18
10#1
kDa
180
100
70
70
55
40
130
35
25
15
10
chl1
chl2
Dasa2h
Dasa24h
Brig2h
Brig24h
D+B 2h
D+B 24h
Simv.2h
Simv24h
D+S 2h
D+S 24h
pS6
PYK2
pGSK
pStat3
pErbB3
5min

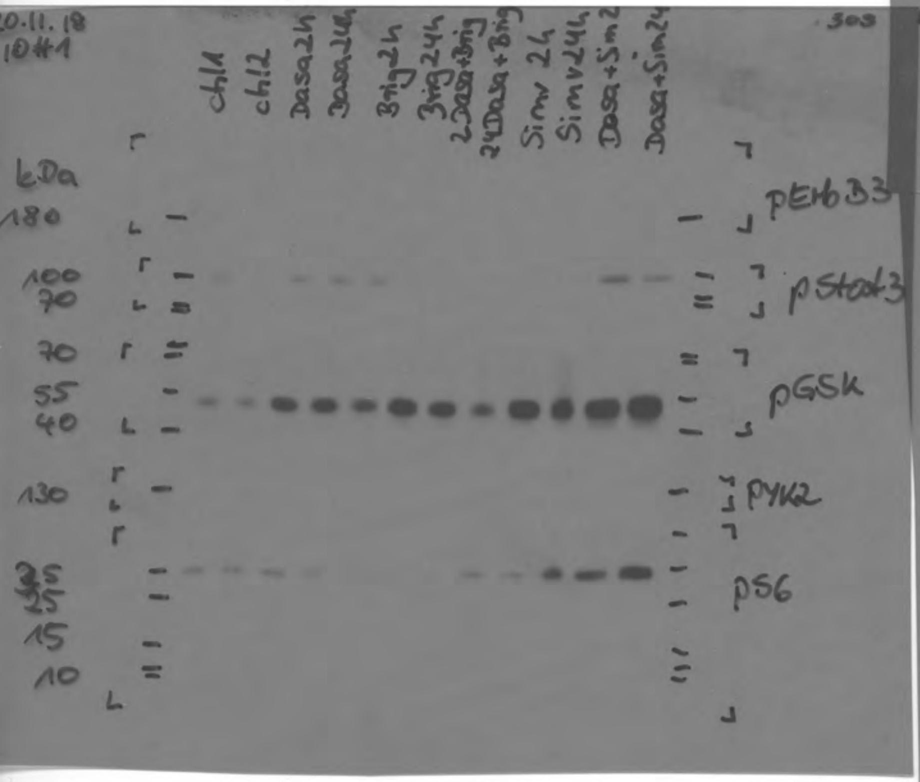
20.11.18
10#1
30s
chl1 chl2 Dasa2h Dasa24h Brig2h Brig24h 2hDasa+Brig 24hDasa+Brig Simv 2h Simv24h Dasa+Sim2 Dasa+Sim24
kDa
180
100
70
70
55
40
130
35
25
15
10
pErbB3
pStat3
pGSK
PYK2
pS6

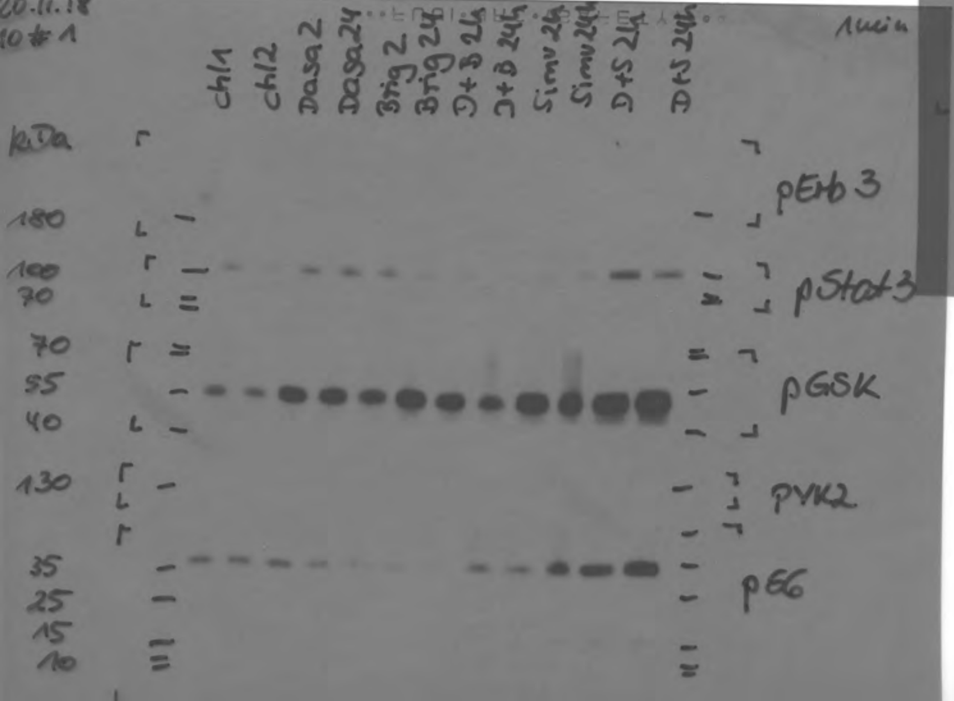
20.11.18
10#1
1min
chl1 chl2 Dasa2 Dasa24 Brig 2 Brig 24 D+B 2h D+B 24h Simv 2h Simv 24h D+S 2h D+S 24h
kDa
180
100
70
70
55
40
130
35
25
15
10
pErb3
pStat3
pGSK
PYK2
pS6

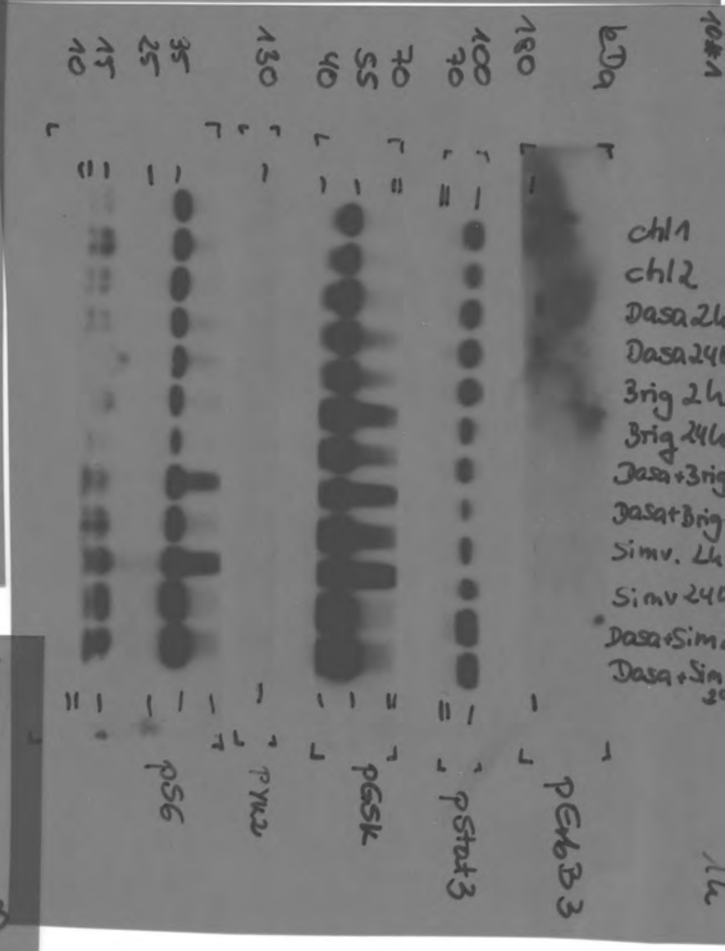
10#1
kDa
180
100
70
70
55
40
130
35
25
15
10
chl1
chl2
Dasa2h
Dasa24h
Brig 2h
Brig24h
Dasa+Brig
Dasa+Brig
Simv. 2h
Simv24h
Dasa+Sim
Dasa+Sim
pS6
PYK2
pGSK
pStat3
pErbB3

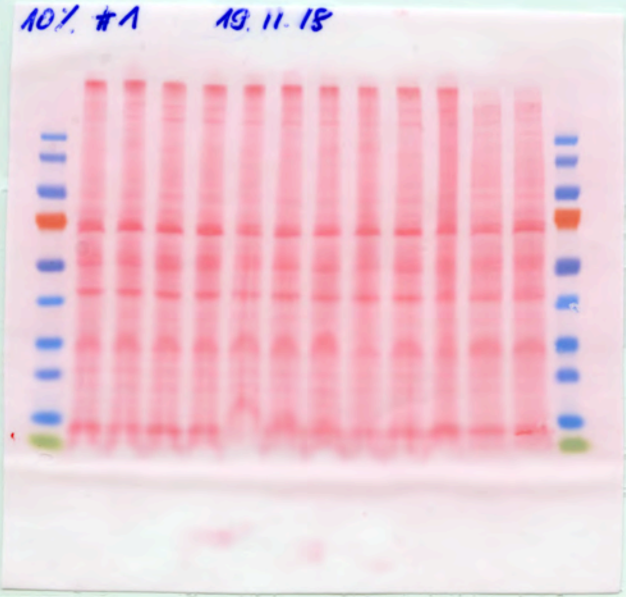
10% #1 19.11.18

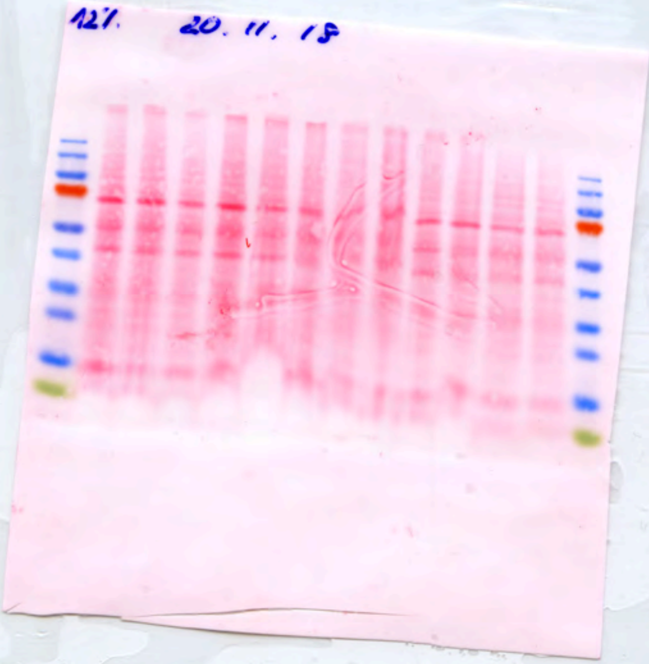
12% 20.11.18
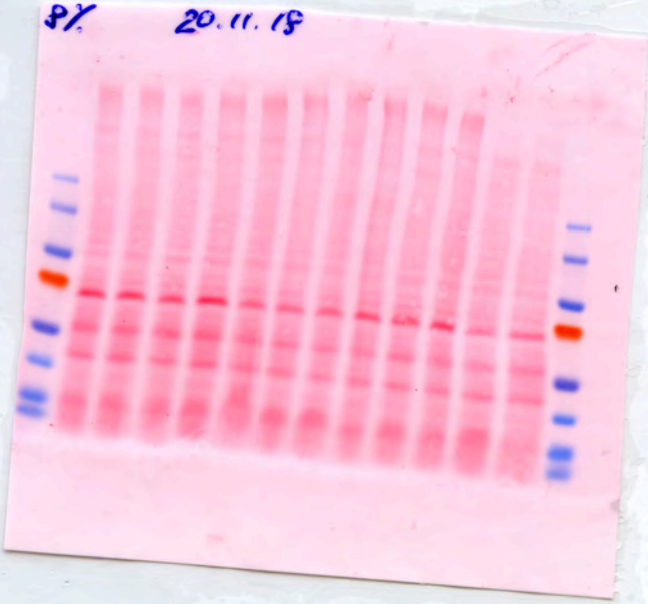
8% 20.11.18
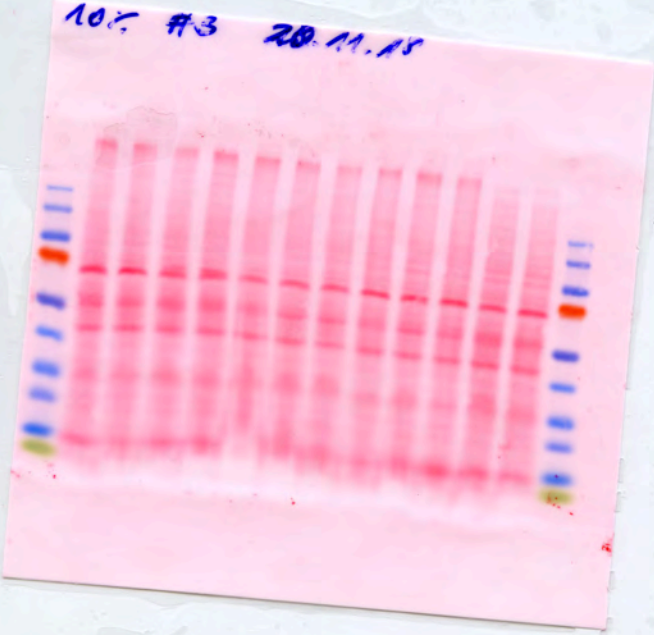
10% #3 20.11.18
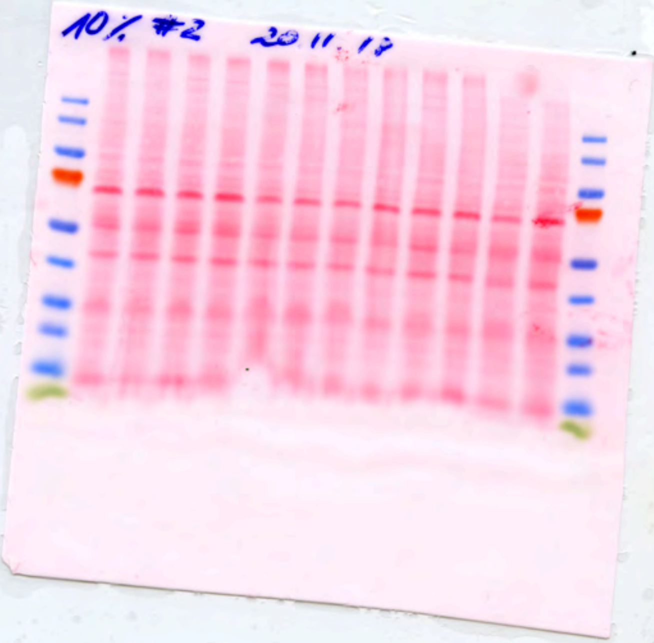
10% #2 20.11.18

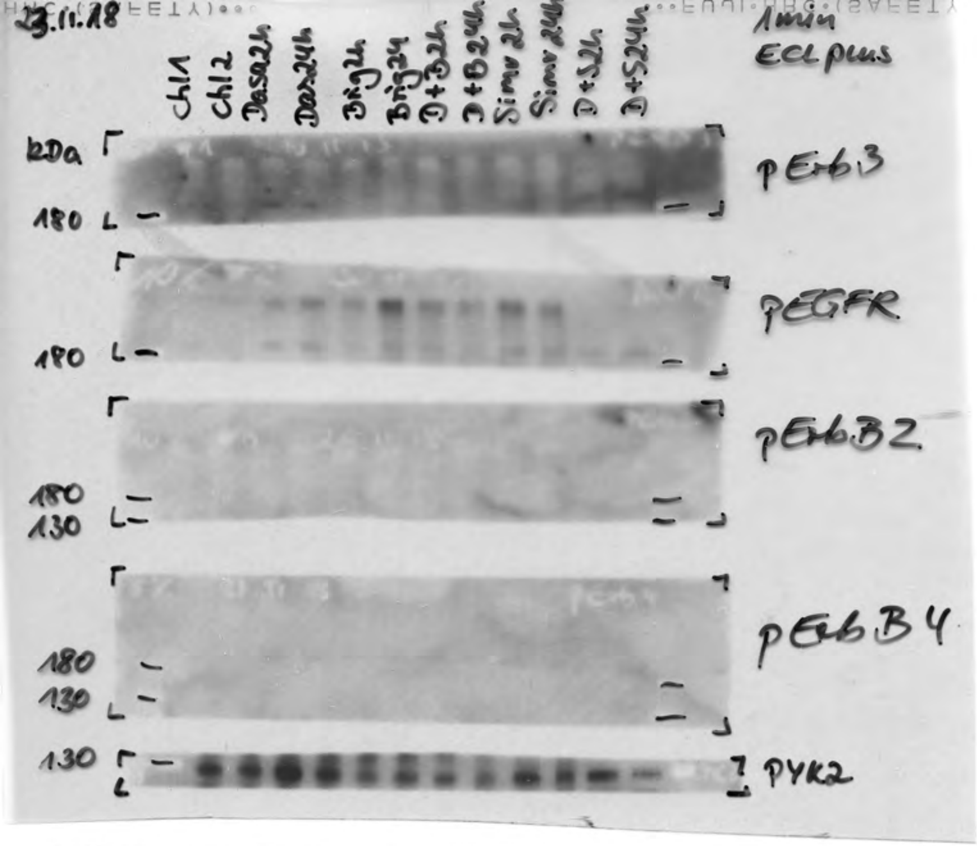
23.11.18
1min
ECL plus
Ctl1
Ctl2
Dasa2h
Dasa24h
Brig2h
Brig24
D+B2h
D+B24h
Simv2h
Simv24h
D+S2h
D+S24h
kDa
180
pErbB3
180
pEGFR
180
130
pErbB2
180
130
pErbB4
130
PYK2

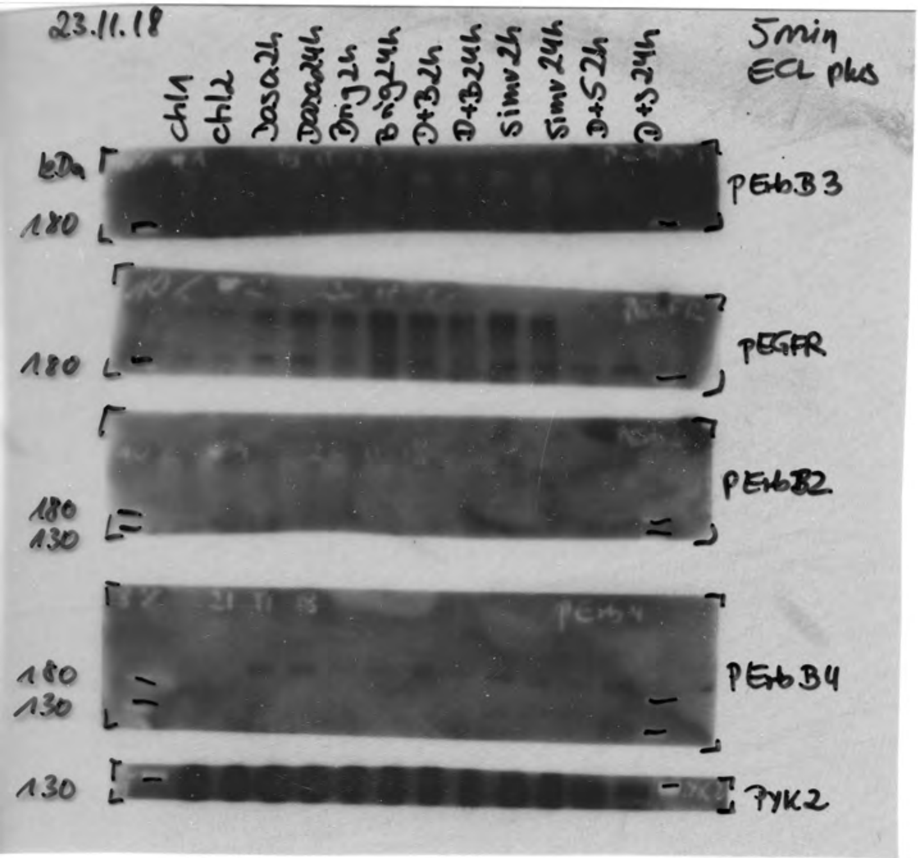
23.11.18
5min
ECL plus
Ctl1
Ctl2
Dasa2h
Dasa24h
Brig2h
Brig24h
D+B2h
D+B24h
Simv2h
Simv24h
D+S2h
D+S24h
kDa
180
pErbB3
180
pEGFR
180
130
pErbB2
180
130
pErbB4
130
PYK2

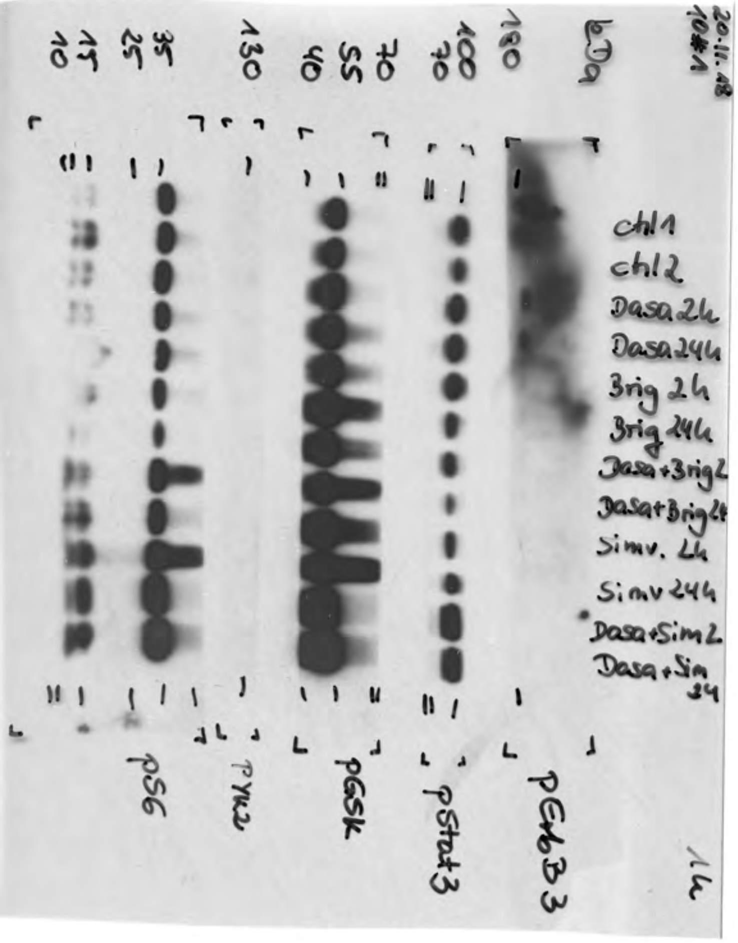
20.11.18
10#1
kDa
180
100
70
70
55
40
130
35
25
15
10
ctrl1
ctrl2
Dasa2h
Dasa24h
Brig 2h
Brig 24h
Dasa+Brig2
Dasa+Brig24
Simv. 2h
Simv24h
Dasa+Sim2
Dasa+Sim 24
pErbB3
pStat3
pGSK
PYK2
pS6
1h

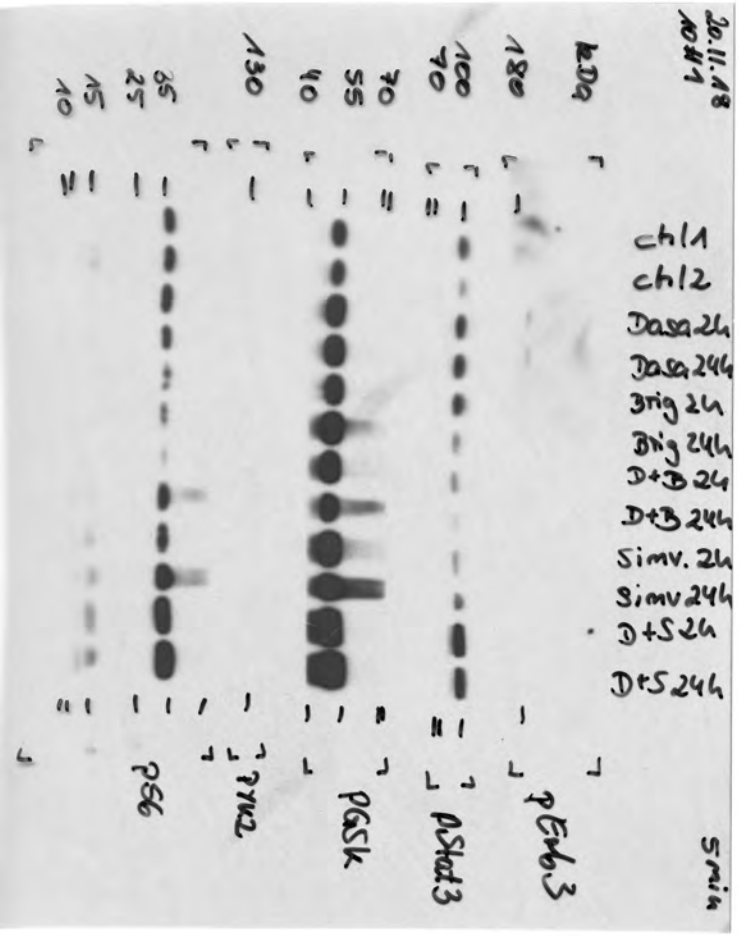
20.11.18
10#1
kDa
180
100
70
70
55
40
130
35
25
15
10
ctrl1
ctrl2
Dasa2h
Dasa24h
Brig 2h
Brig 24h
D+B 2h
D+B 24h
Simv. 2h
Simv24h
D+S2h
D+S24h
pErb3
pStat3
pGSK
PYK2
pS6
5min

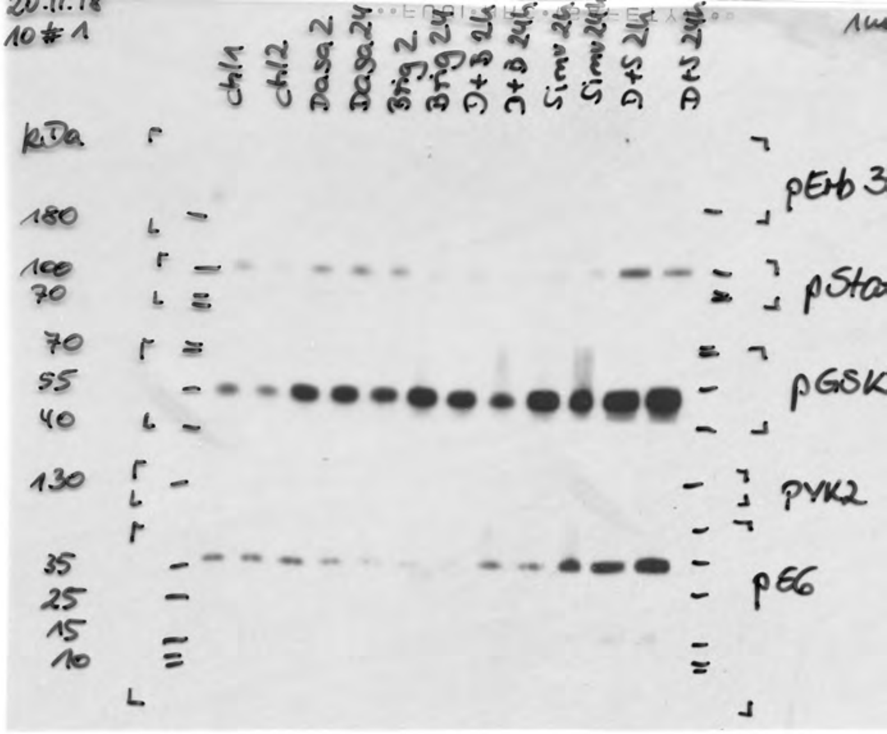
20.11.18
10#1
ctrl1
ctrl2
Dasa 2
Dasa 24
Brig 2
Brig 24
D+B 2h
D+B 24h
Simv 2h
Simv 24h
D+S 2h
D+S 24h
kDa
180
100
70
70
55
40
130
35
25
15
10
pErb3
pStat
pGSK
PYK2
pS6

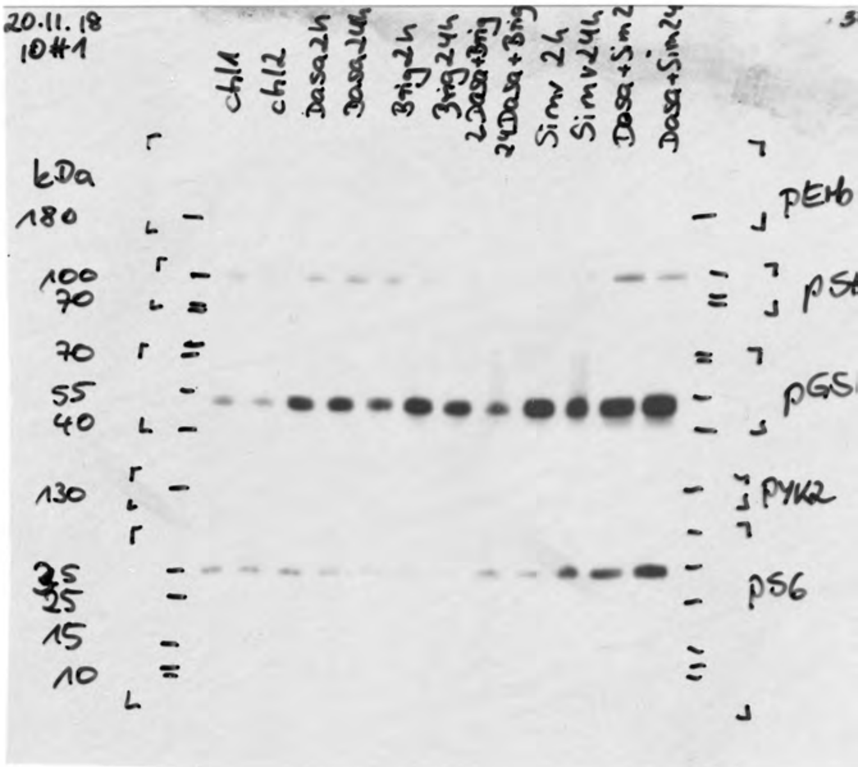
20.11.18
10#1
ctrl1
ctrl2
Dasa2h
Dasa24h
Brig2h
Brig24h
2Dasa+Brig
24Dasa+Brig
Simv 2h
Simv24h
Dasa+Sim2
Dasa+Sim24
kDa
180
100
70
70
55
40
130
35
25
15
10
pErb
PYK2
pS6

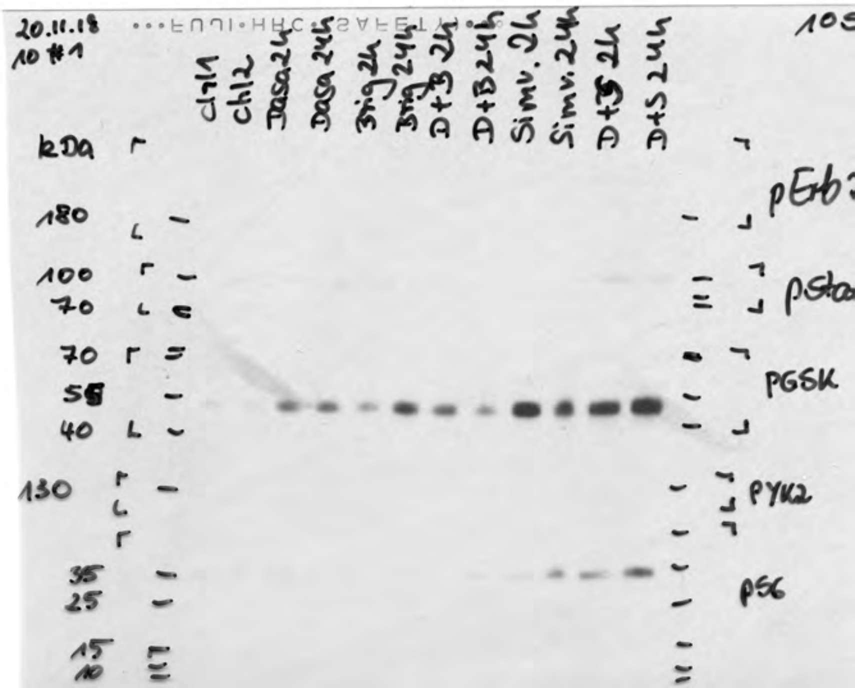
20.11.18
10#1
ctrl1
ctrl2
Dasa2h
Dasa 24h
Brig 2h
Brig 24h
D+B 2h
D+B 24h
Simv. 2h
Simv. 24h
D+S 2h
D+S 24h
kDa
180
100
70
70
55
40
130
35
25
15
10
pErb3
pStat
pGSK
PYK2
pS6

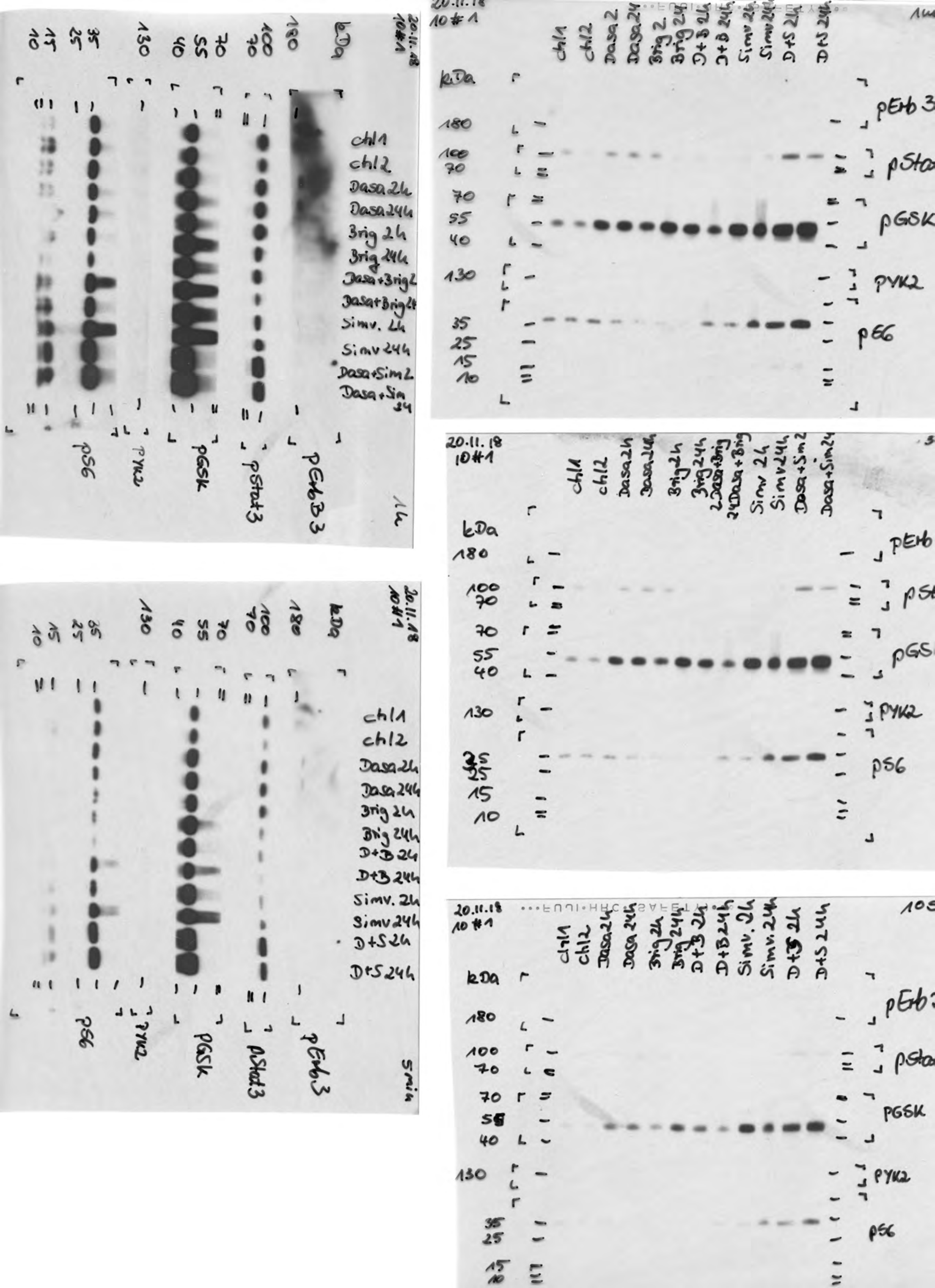

20.11.18
10#1
kDa
180
100
70
55
40
130
35
25
15
10
ctl1
ctl2
Dasa 2h
Dasa 24h
Brig 2h
Brig 24h
D+B 2h
D+B 24h
Simv. 2h
Simv 24h
D+S 2h
D+S 24h
Dasa+Brig 2
Dasa+Brig 24
Dasa+Sim 2
Dasa+Sim 24
pErb3
pStat3
pGSK
PYK2
pS6
1h
5min

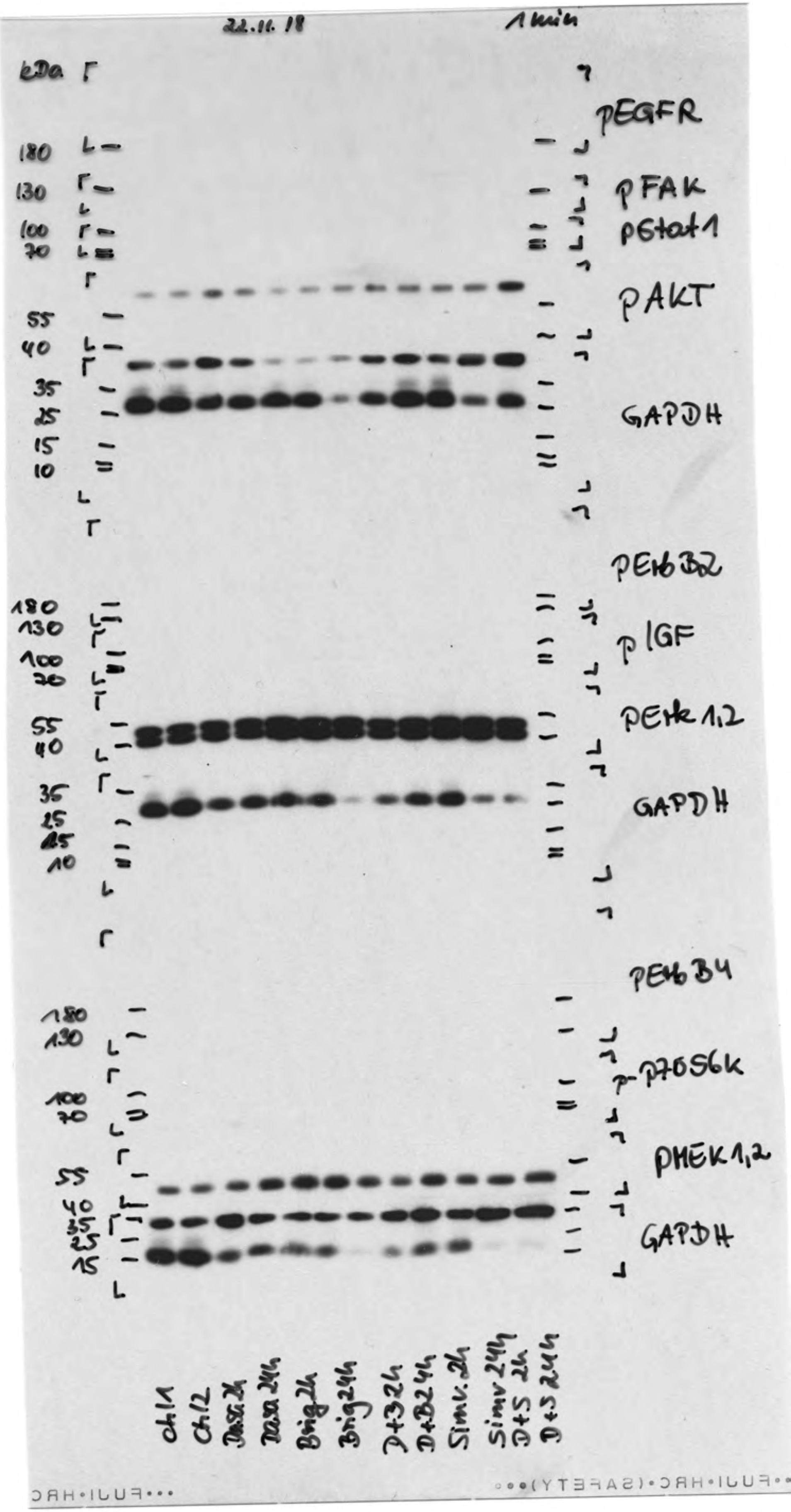
22.11.18
1 min
kDa
pEGFR
pFAK
pStat1
pAKT
GAPDH
pErbB2
pIGF
pErk1,2
GAPDH
pErbB4
p-p70S6K
pMEK1,2
GAPDH
Ctl 1
Ctl 2
Dasa 2h
Dasa 24h
Brig 2h
Brig 24h
D+B 2h
D+B 24h
Simv. 2h
Simv 24h
D+S 2h
D+S 24h

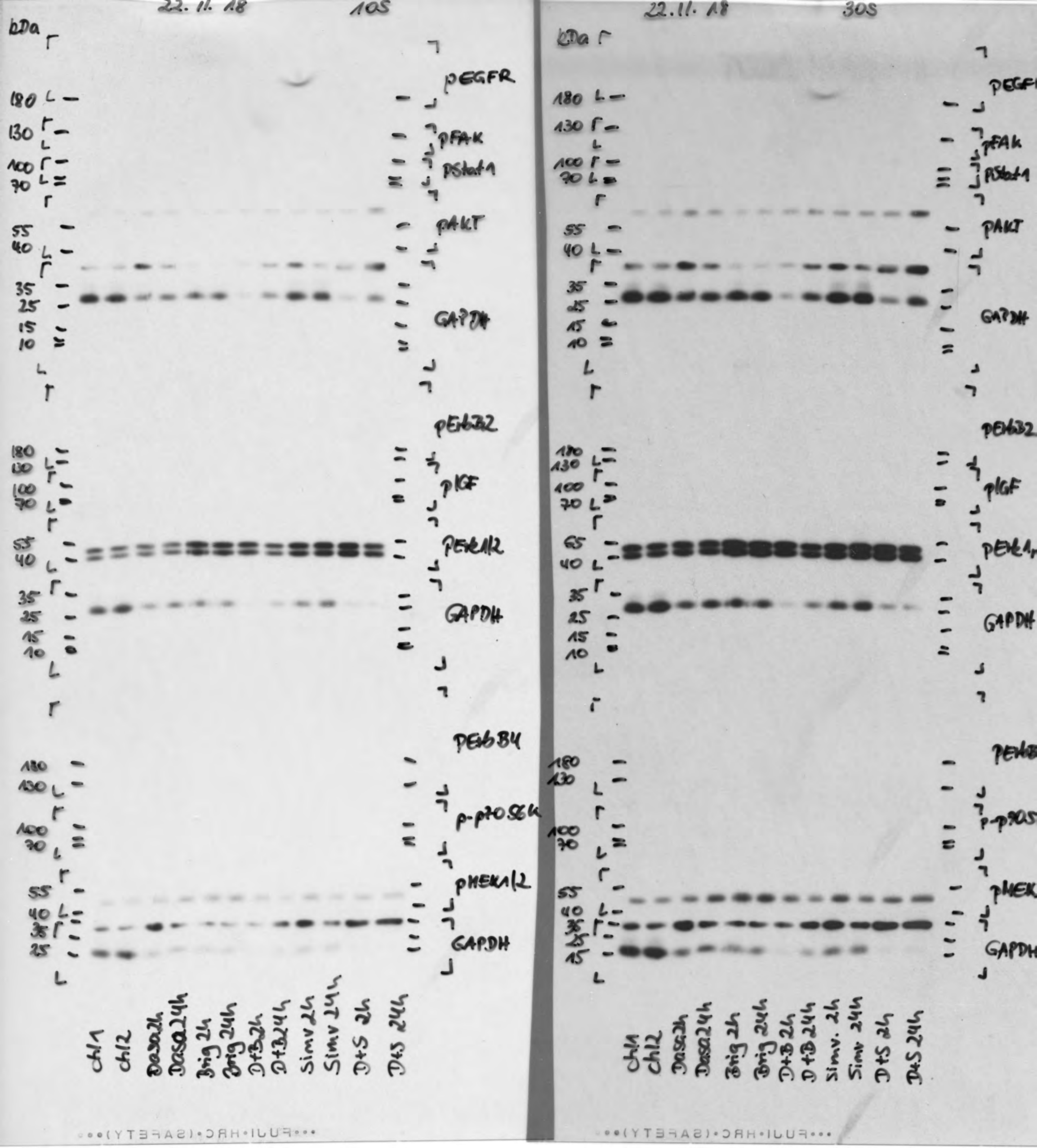

22.11.18
10s
kDa
pEGFR
pFAK
pStat1
pAKT
GAPDH
pErbB2
pIGF
pERK1/2
GAPDH
pErbB4
p-p70S6K
pMEK1/2
GAPDH
ctl1
ctl2
Dasa2h
Dasa24h
Brig 2h
Brig 24h
D+B 2h
D+B 24h
Simv 2h
Simv 24h
D+S 2h
D+S 24h
22.11.18
30s
kDa
ctl1
ctl2
Dasa2h
Dasa24h
Brig 2h
Brig 24h
D+B 2h
D+B 24h
Simv. 2h
Simv 24h
D+S 2h
D+S 24h

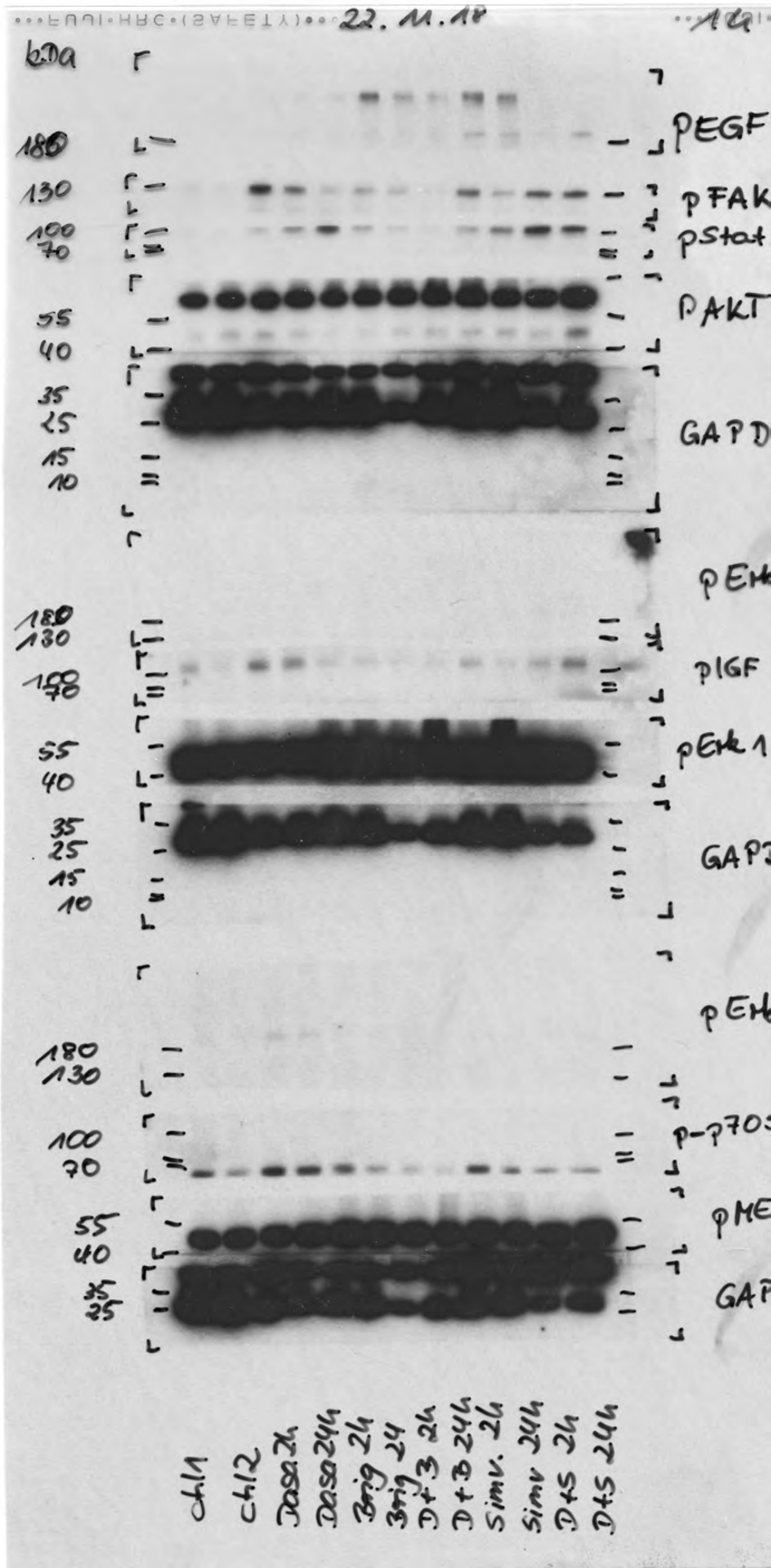

22.11.18
kDa
180
130
100
70
55
40
35
25
15
10
PEGF
pFAK
pStat
pAKT
GAPD
pErk
pIGF
pErk1
GAP
p-p70
pME
GAP
ctl1
ctl2
Dasa 2h
Dasa 24h
Brig 2h
Brig 24
D+B 2h
D+B 24h
Simv. 2h
Simv 24h
D+S 2h
D+S 24h

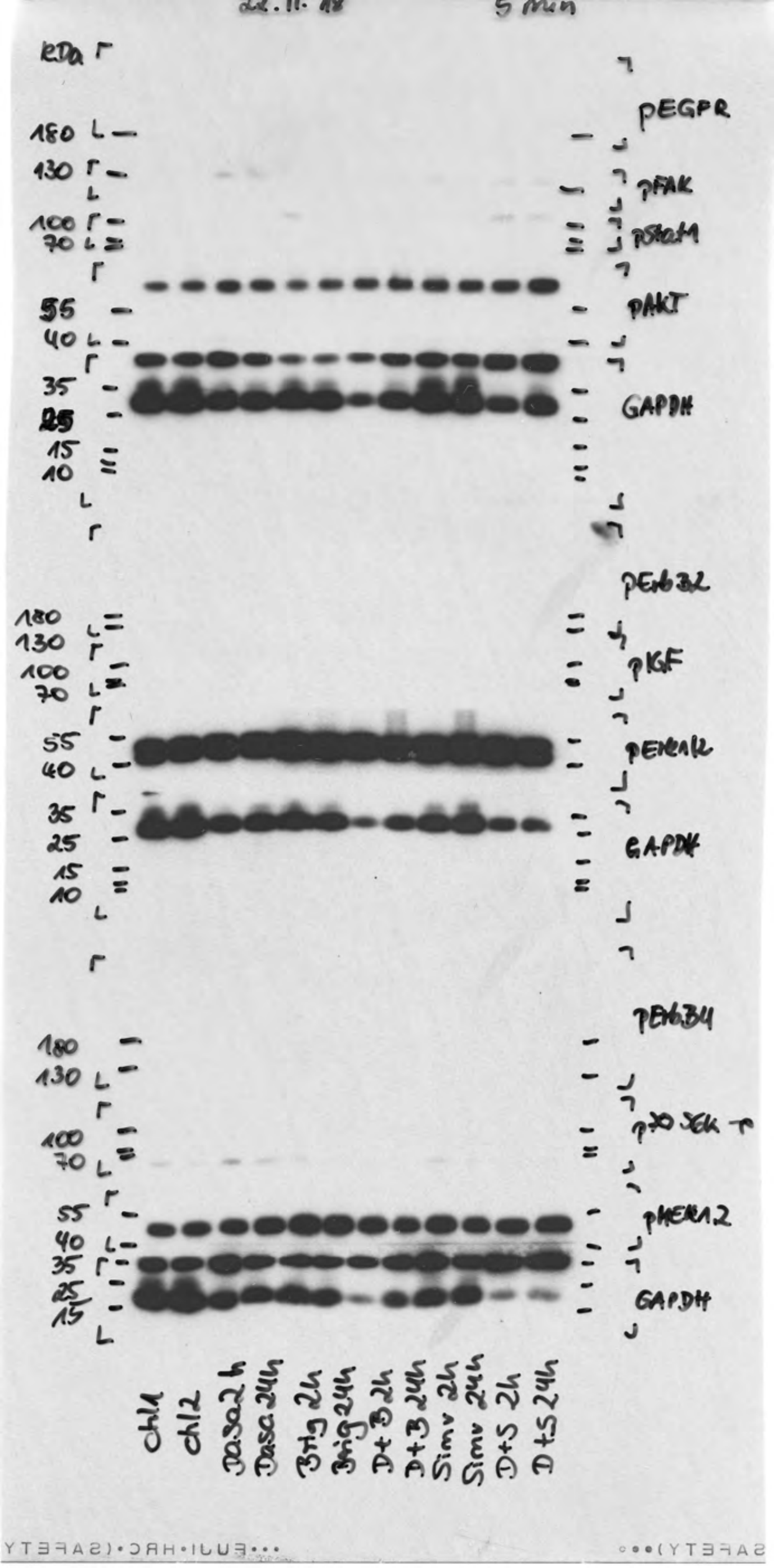
5 min
kDa
180
130
100
70
55
40
35
25
15
10
pEGFR
pFAK
pAKT
GAPDH
pIGF
GAPDH
180
130
100
70
55
40
35
25
15
10
GAPDH
ctrl1
ctrl2
Dasa 2h
Dasa 24h
D+B 2h
D+B 24h
Simv 2h
Simv 24h
D+S 2h
D+S 24h

15

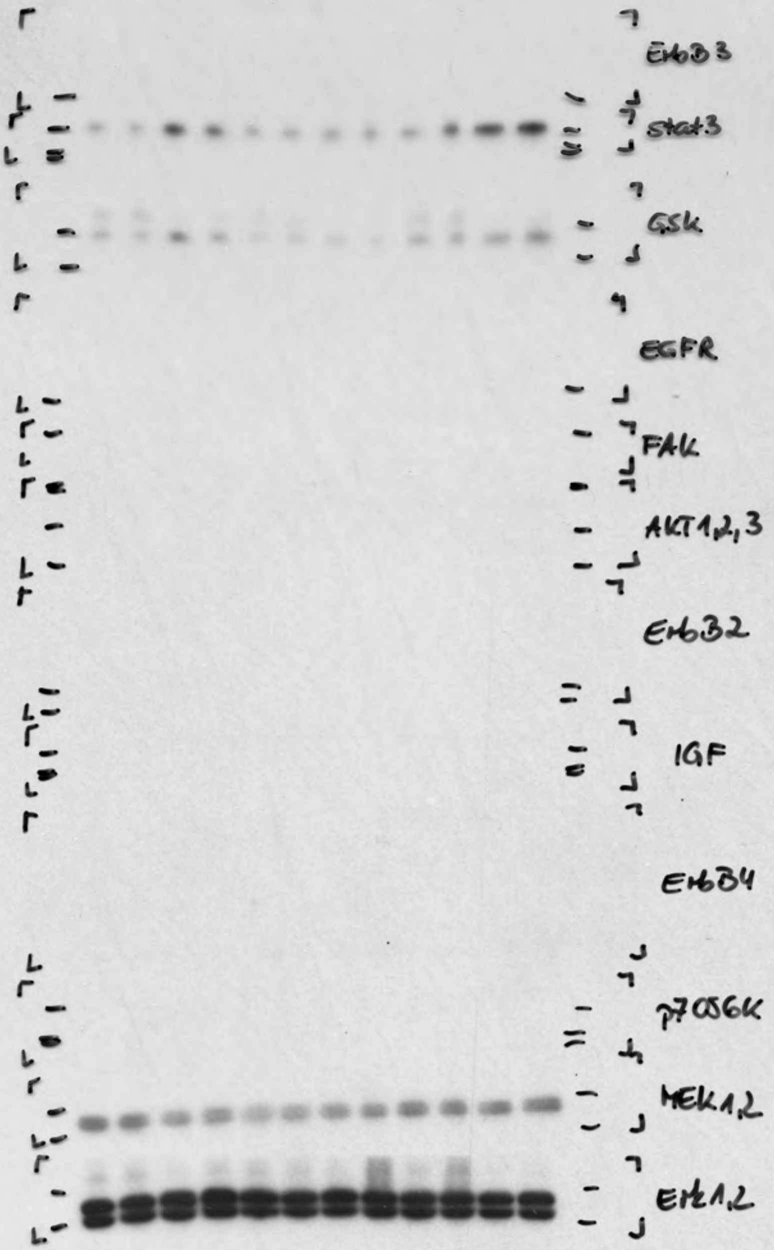

ErbB3
stat3
GSK
EGFR
FAK
AKT1,2,3
ErbB2
IGF
ErbB4
p70S6K
MEK1,2
Erk1,2

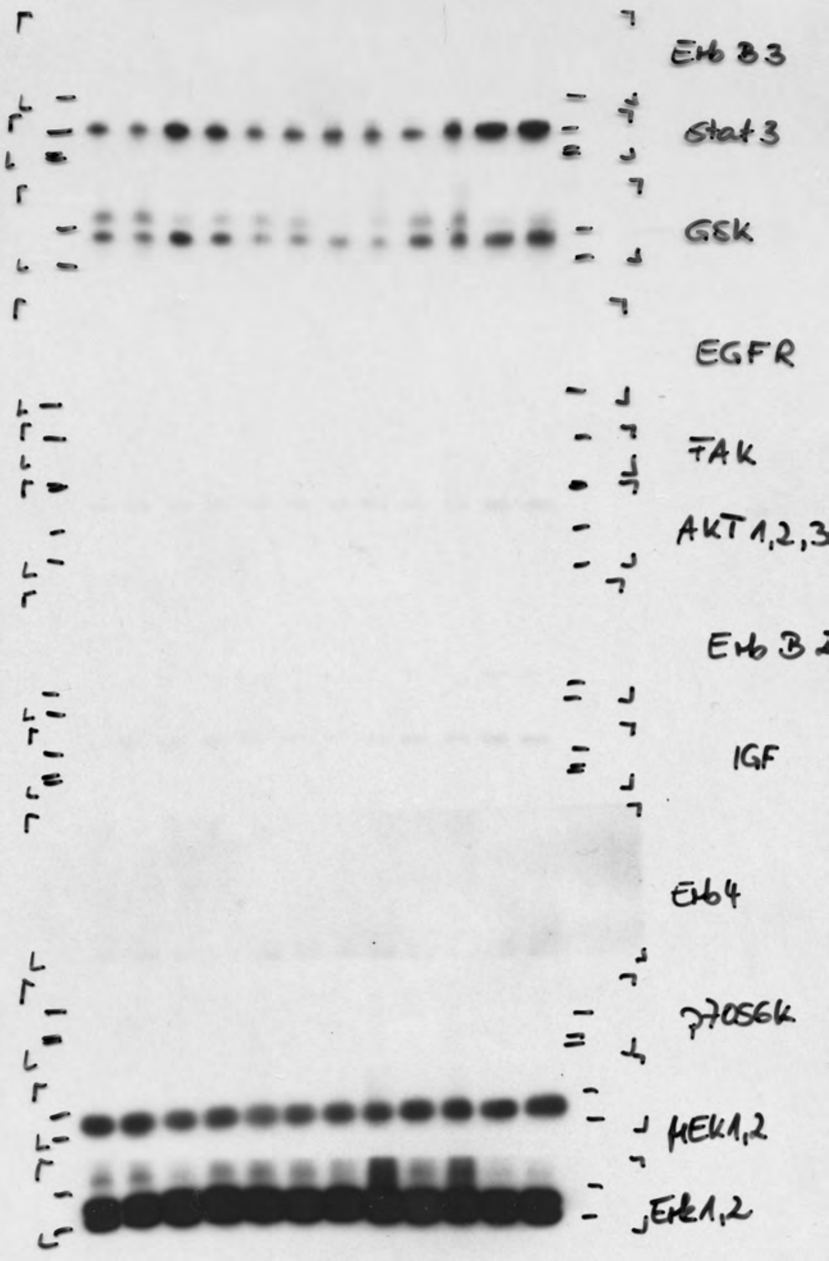

Erb B3
Stat3
GSK
EGFR
FAK
AKT1,2,3
ErbB2
IGF
Erb4
p70S6k
MEK1,2
Erk1,2

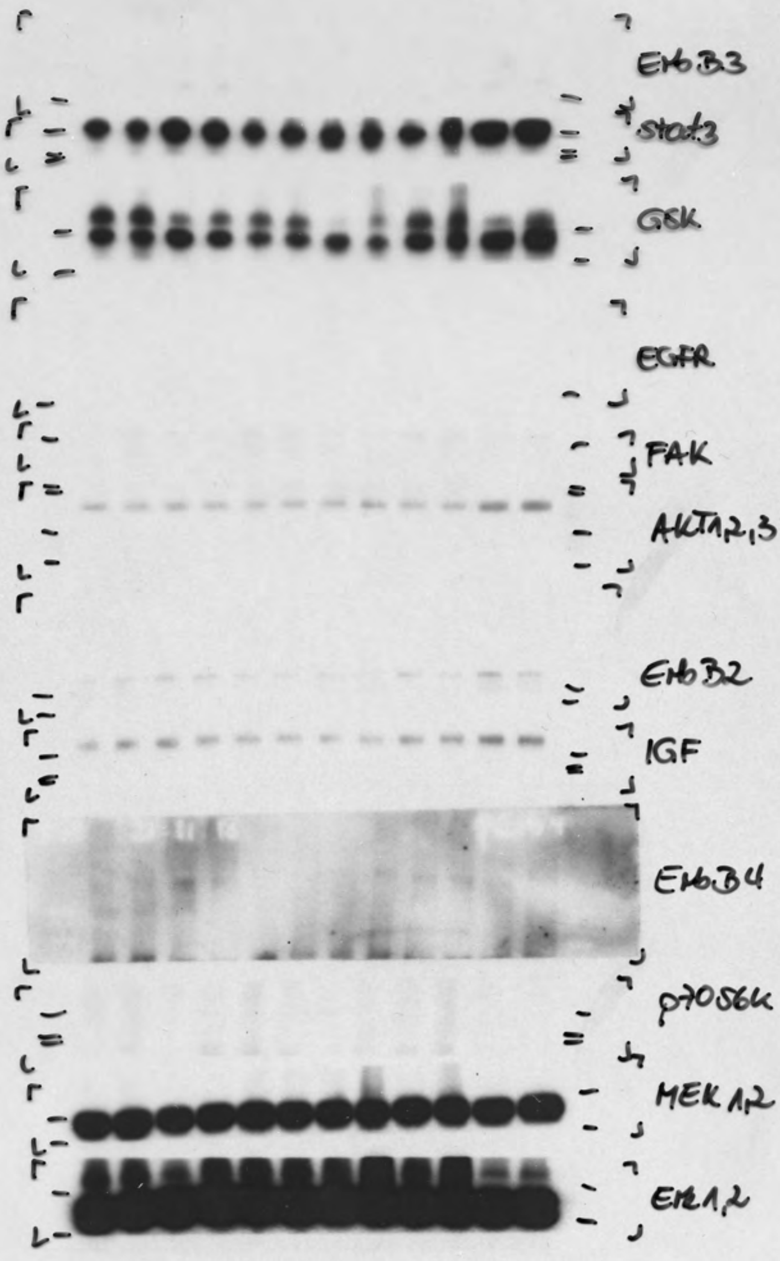
ErbB3
stat3
GSK
EGFR
FAK
AKT1,2,3
ErbB2
IGF
ErbB4
p70S6k
MEK1,2
Erk1,2

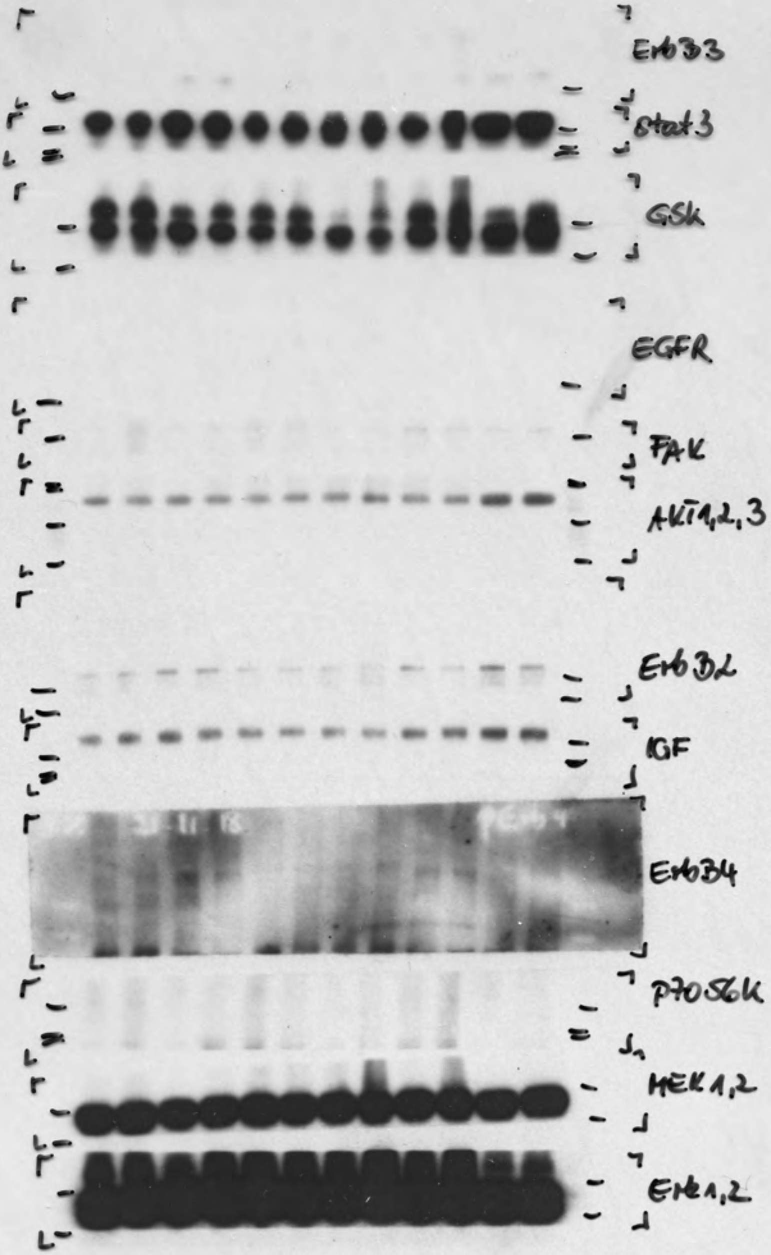

ErbB3
Stat3
GSK
EGFR
FAK
AKT1,2,3
ErbB2
IGF
ErbB4
p70S6K
MEK1,2
Erk1,2

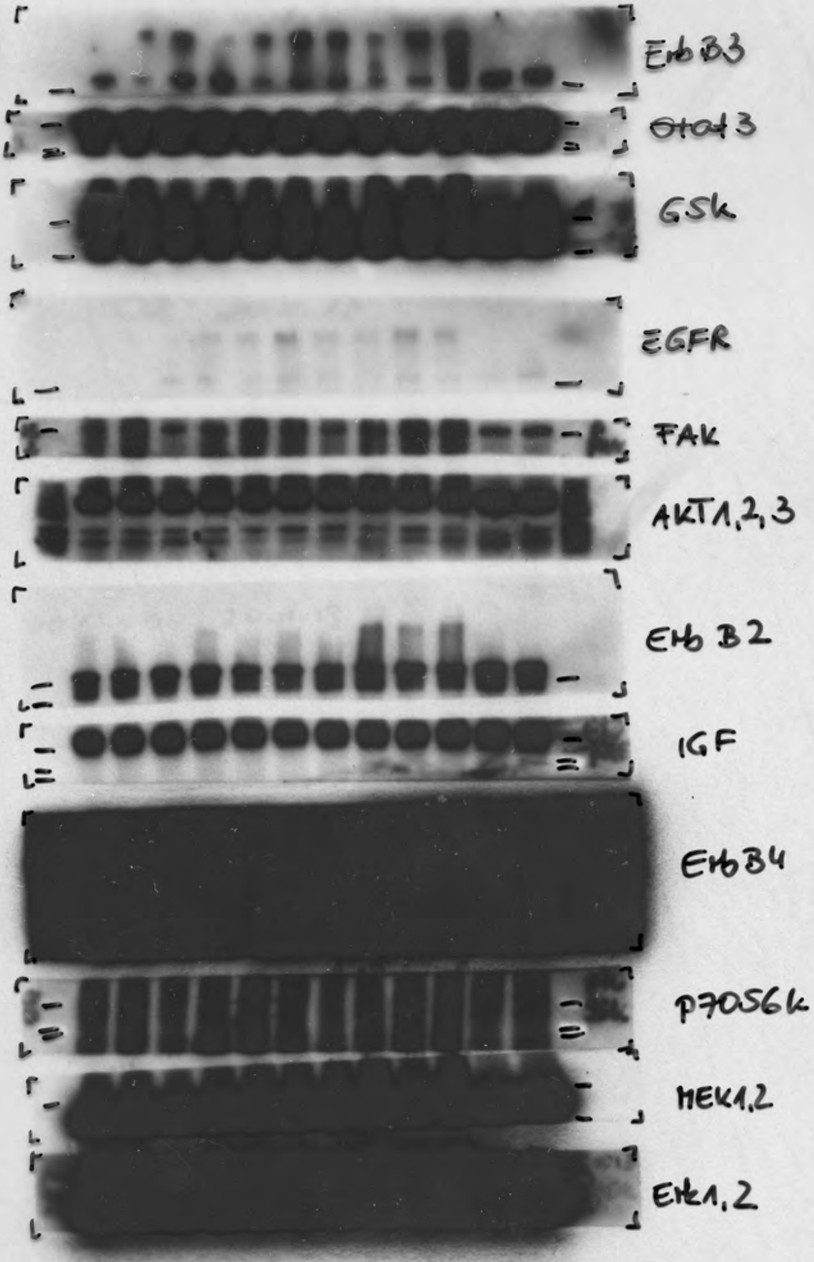
ErbB3
Stat3
GSK
EGFR
FAK
AKT1,2,3
ErbB2
IGF
ErbB4
p70S6k
MEK1,2
Erk1,2

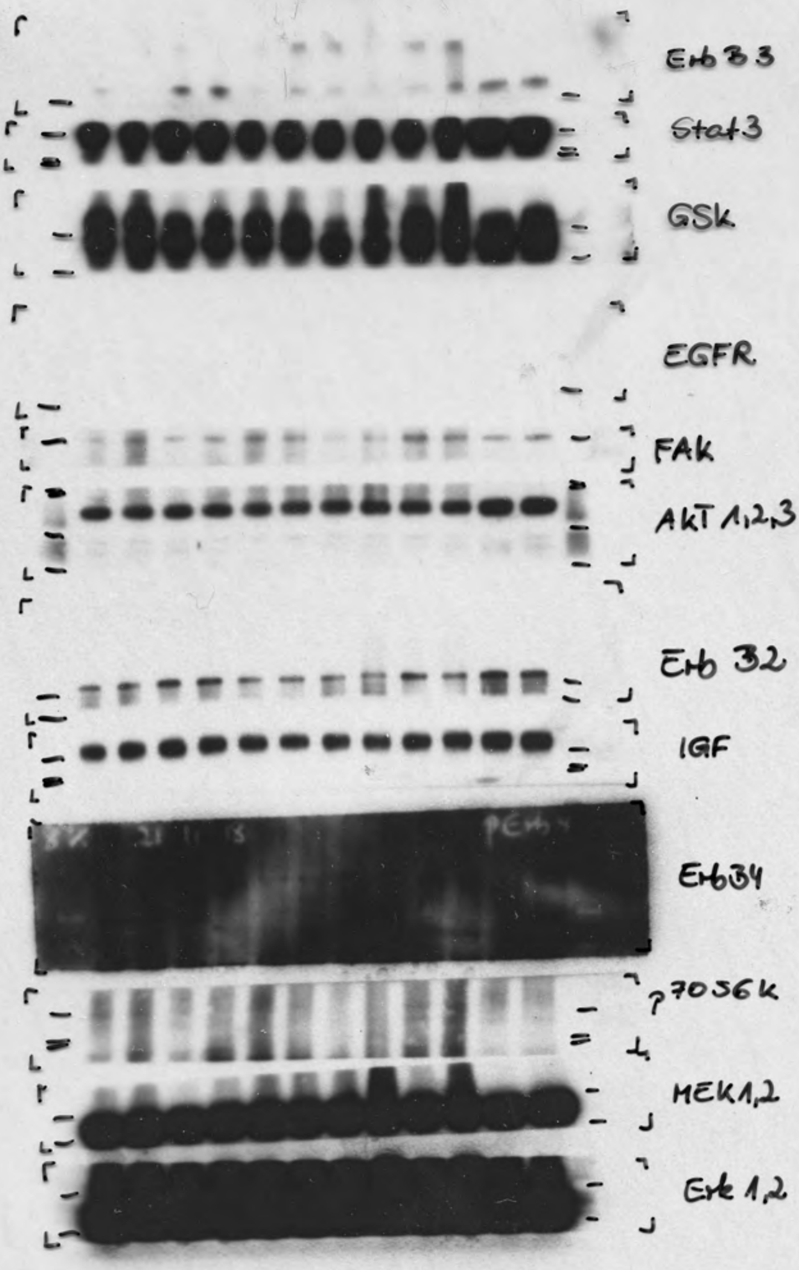

ErbB3
Stat3
GSk
EGFR
FAK
AKT 1,2,3
ErbB2
IGF
ErbB4
p70S6k
MEK1,2
Erk 1,2

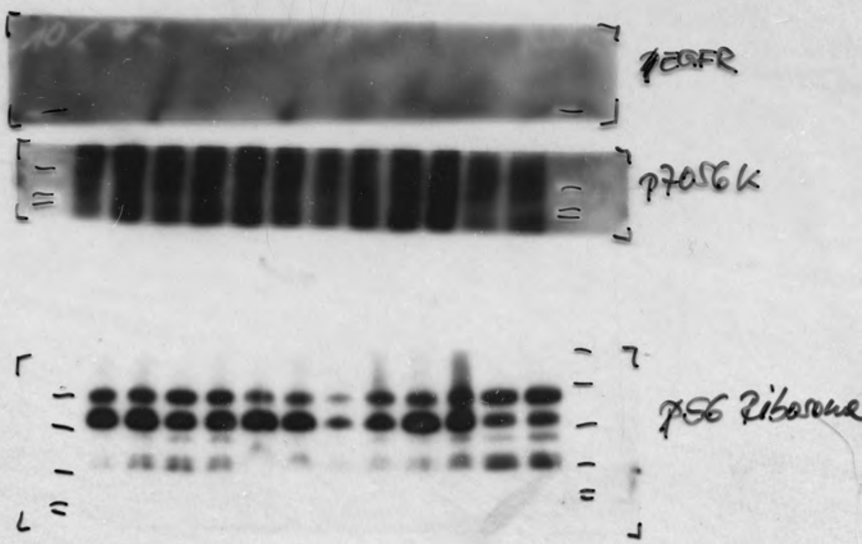
pEGFR
p70S6K
pS6 Ribosome

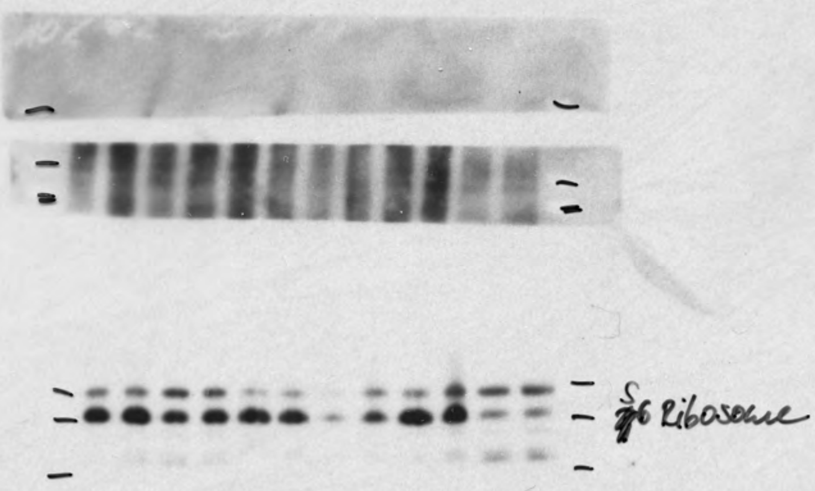
pS6 Ribosome

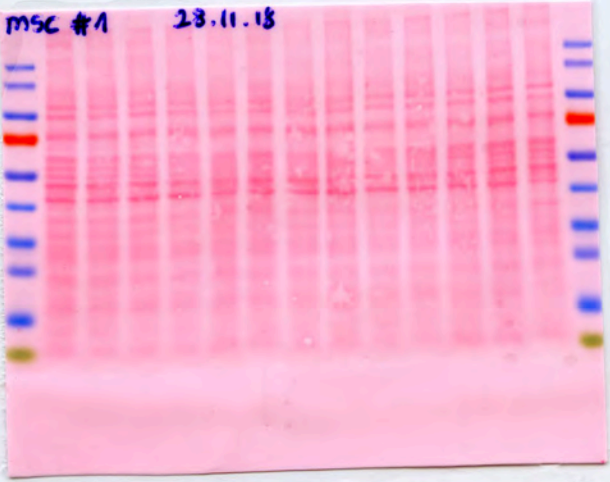
MSC #1
28.11.18

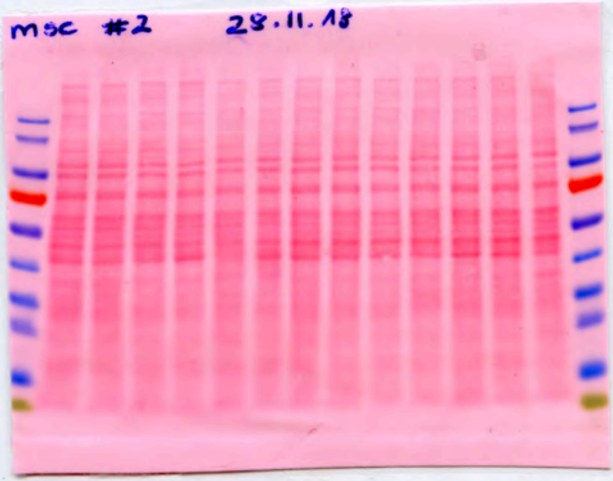
MSC #2
28.11.18

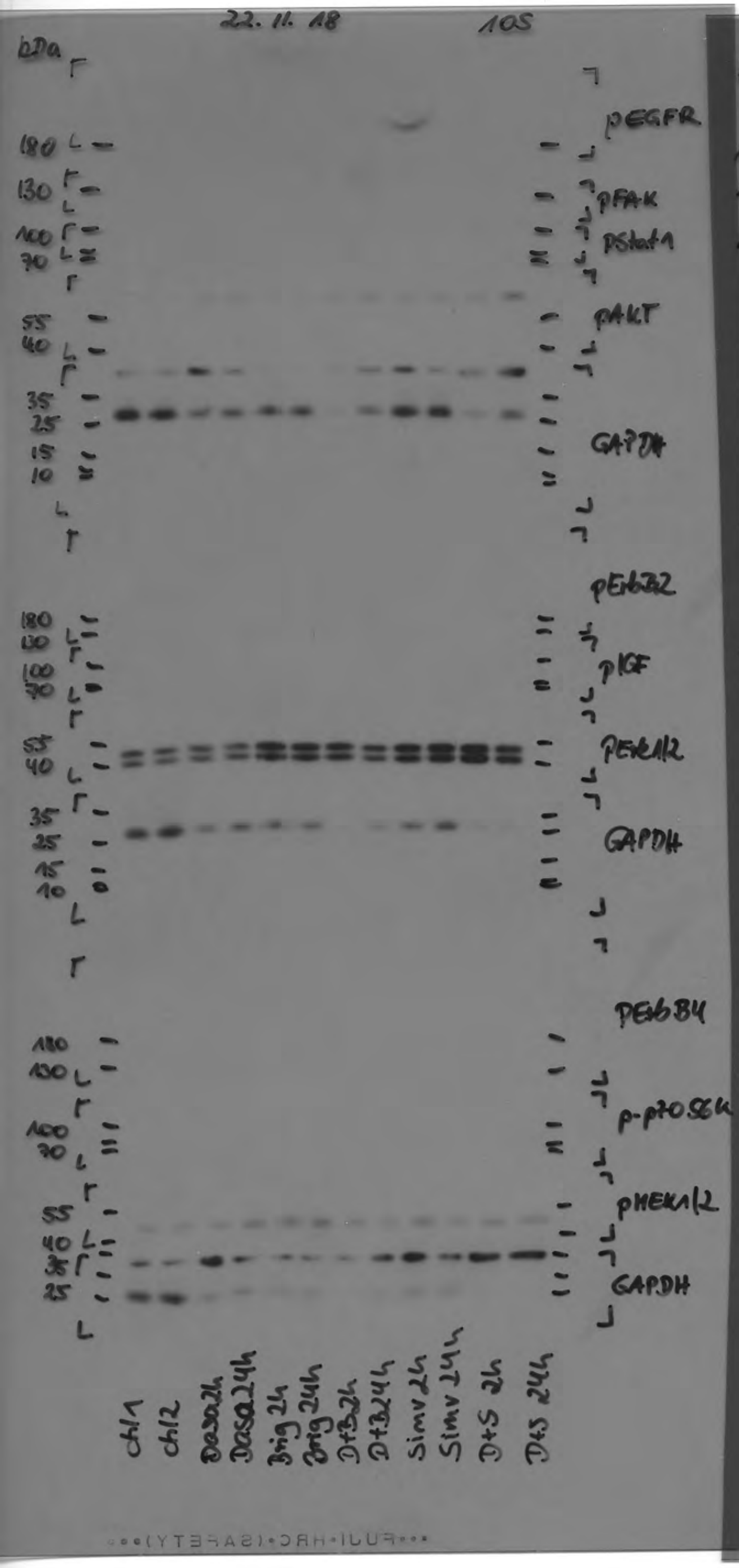
22.11.18
10s
kDa
pEGFR
pFAK
pStat1
pAKT
GAPDH
pErbB2
pIGF
pERK1/2
GAPDH
pErbB4
p-p70S6K
pMEK1/2
GAPDH
180
130
100
70
55
40
35
25
15
10
ctrl1
ctrl2
Dasa 2h
Dasa 24h
Brig 2h
Brig 24h
D+B 2h
D+B 24h
Simv 2h
Simv 24h
D+S 2h
D+S 24h

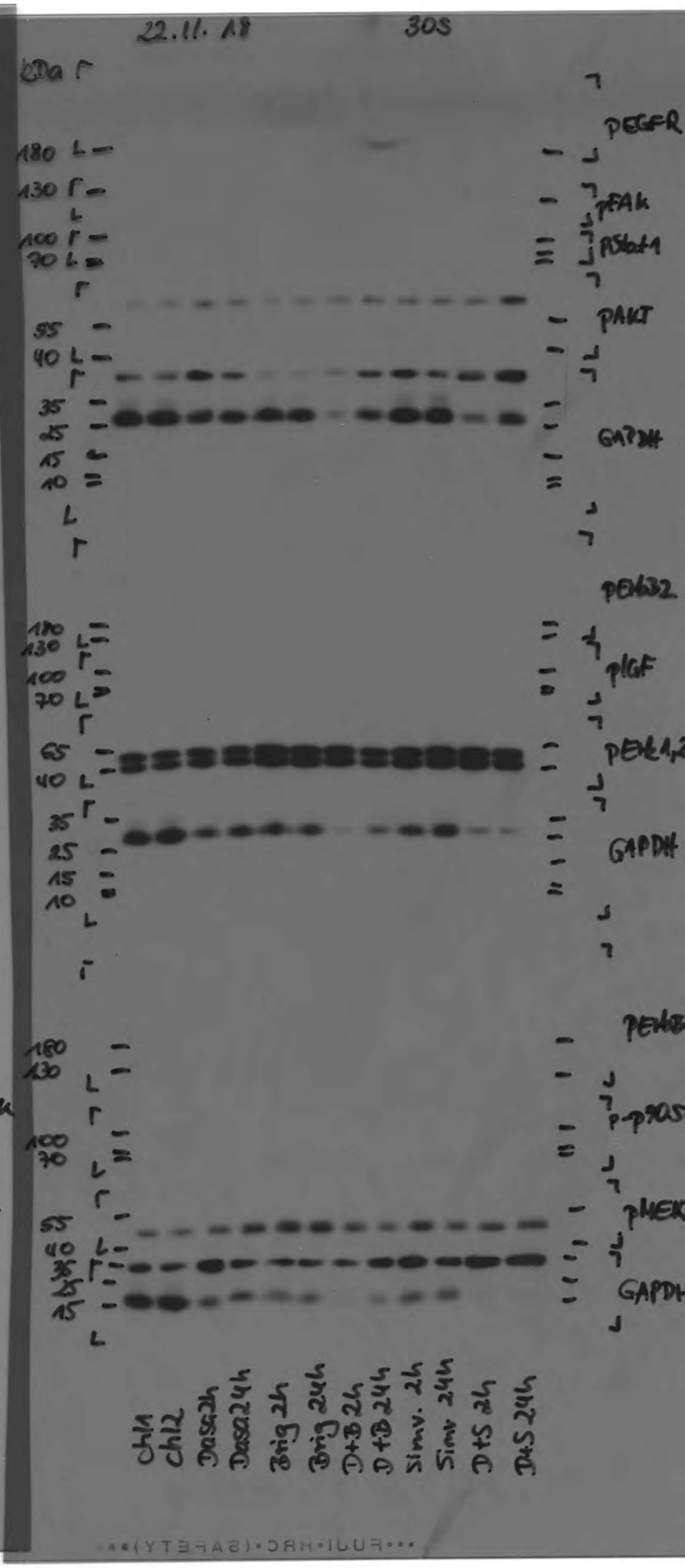
22.11.18
30s
kDa
pEGFR
pFAK
pStat1
pAKT
GAPDH
pErbB2
pIGF
GAPDH
180
130
100
70
55
40
35
25
15
10
ctrl1
ctrl2
Dasa 2h
Dasa 24h
Brig 2h
Brig 24h
D+B 2h
D+B 24h
Simv. 2h
Simv 24h
D+S 2h
D+S 24h

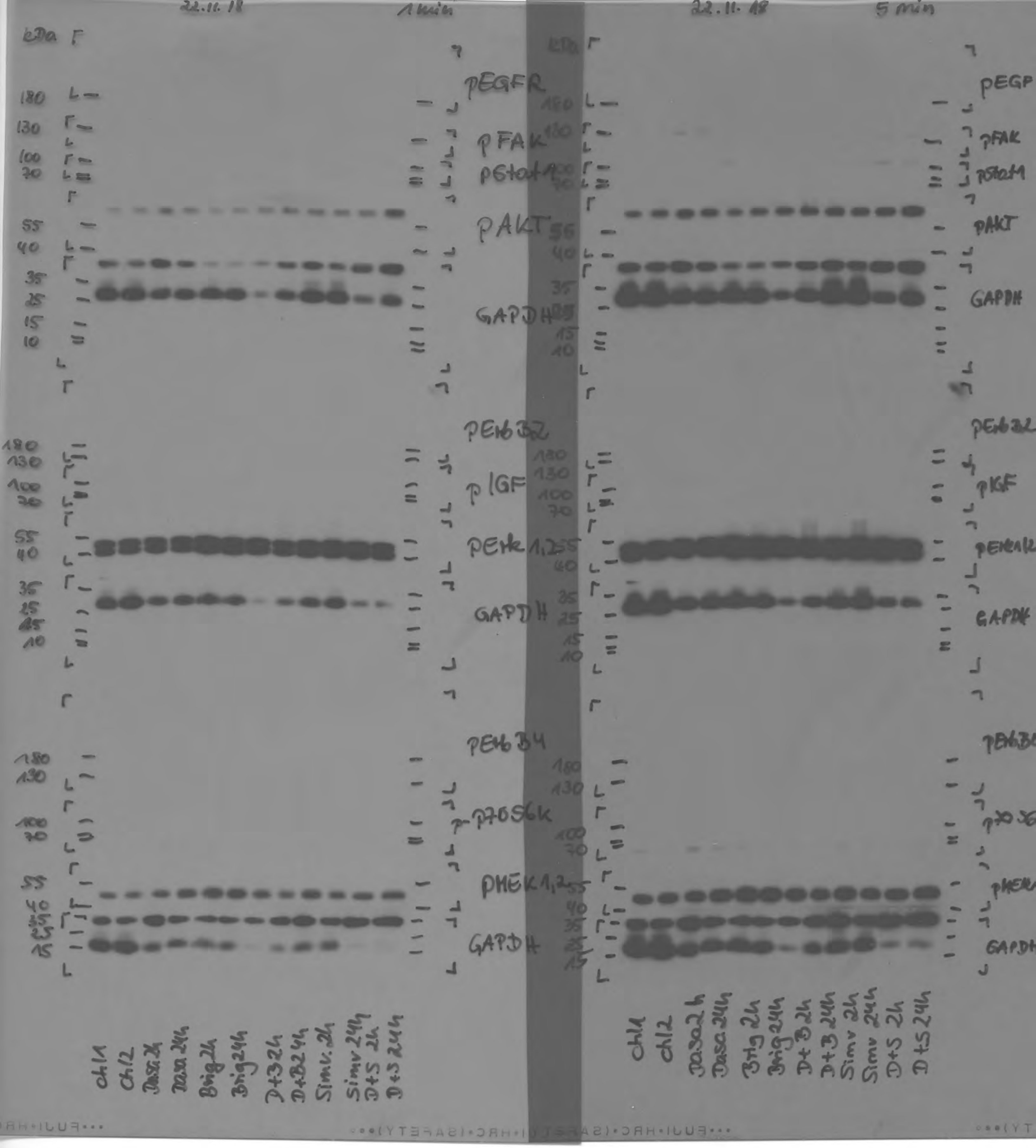
22.11.18
1 min
kDa
180
130
100
70
55
40
35
25
15
10
pEGFR
pFAK
pAKT
GAPDH
pErb B2
pIGF
pErk 1,2
GAPDH
pErb B4
p-P70S6K
pMEK1,2
GAPDH
Ctl 1
Ctl 2
Dasa 2h
Dasa 24h
Brig 2h
Brig 24h
D+B 2h
D+B 24h
Simv. 2h
Simv 24h
D+S 2h
D+S 24h
22.11.18
5 min
FUJI HRC (SAFETY)

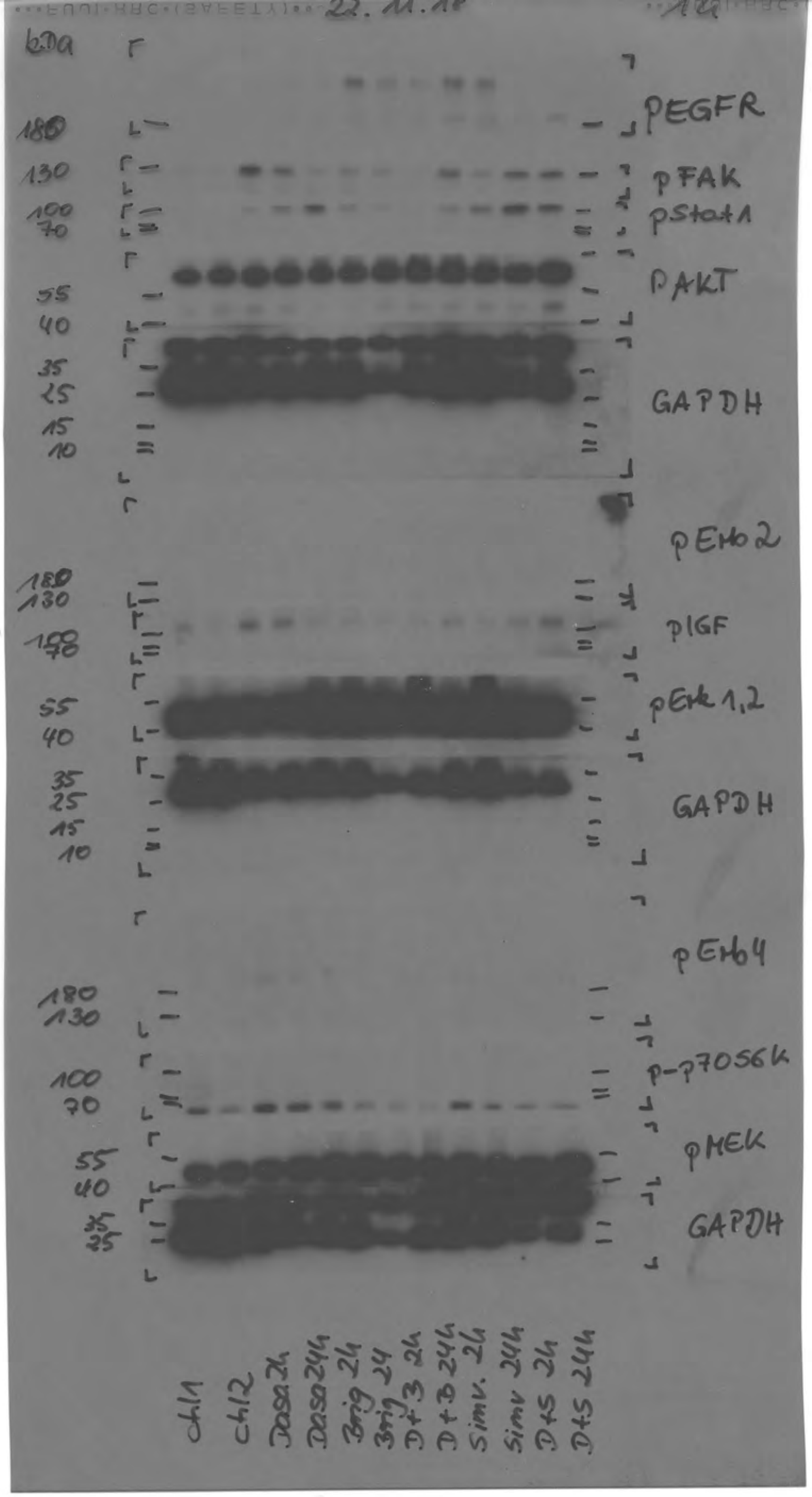
kDa
pEGFR
pFAK
pStat1
pAKT
GAPDH
pErb2
pIGF
pErk1,2
GAPDH
pErb4
p-p70S6K
pMEK
GAPDH
ctl1
ctl2
Dasa 2h
Dasa 24h
Brig 2h
Brig 24
D+B 2h
D+B 24h
Simv. 2h
Simv 24h
D+S 2h
D+S 24h

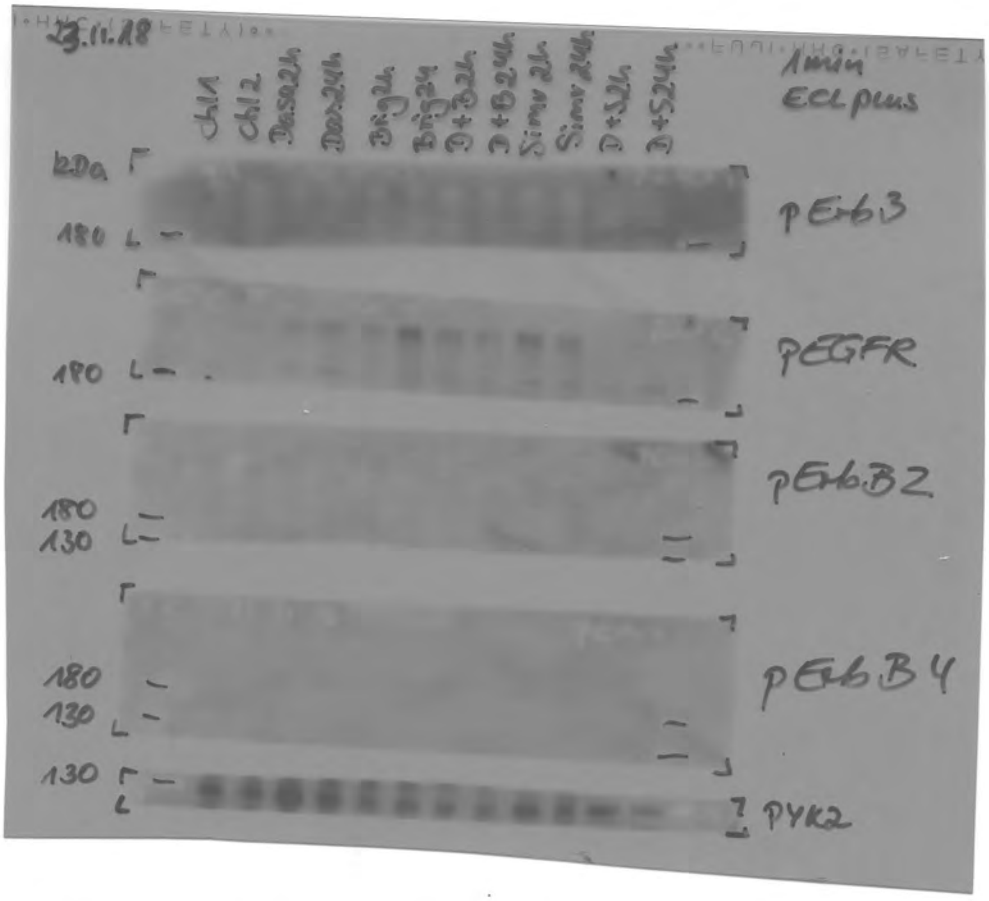
23.11.18
1 min
ECL plus
ctl1
ctl2
kDa
180
pErb3
pEGFR
pErbB2
130
pErbB4
PYK2

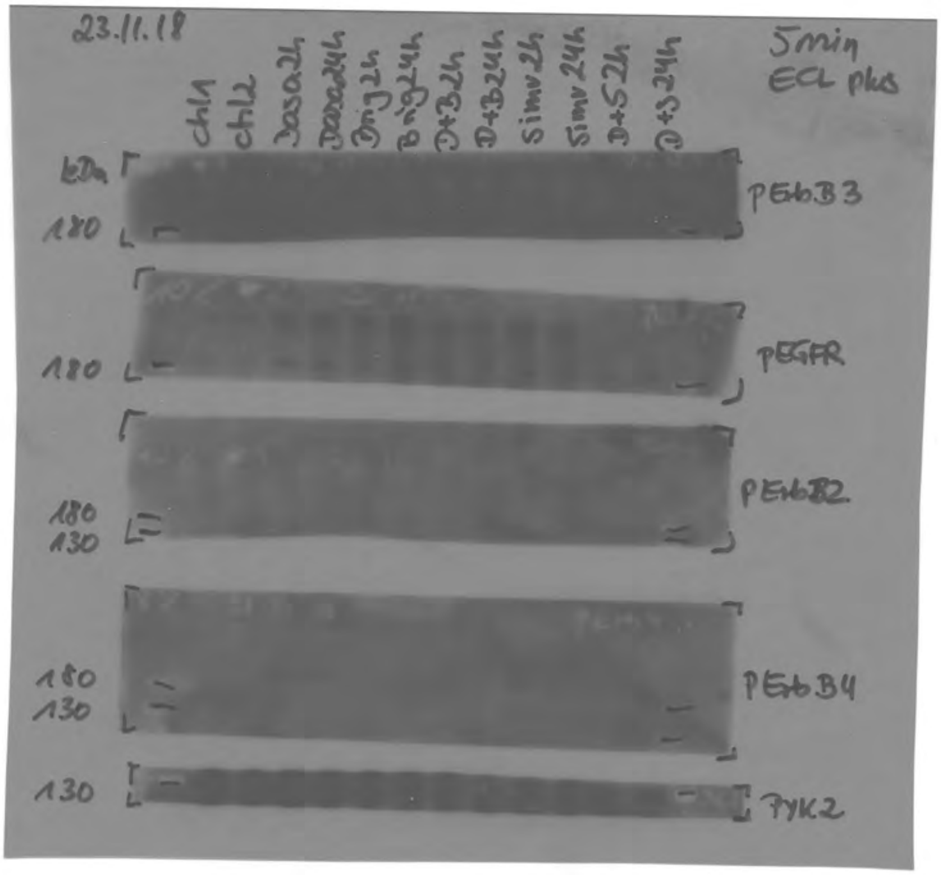
23.11.18
5 min
ECL plus
ctl1
ctl2
kDa
180
pErbB3
pEGFR
pErbB2
130
pErbB4
PYK2
